# Anesthesiologists' Preferences regarding Visitor Presence during Placement of Neuraxial Labor Analgesia

**DOI:** 10.1155/2018/3481975

**Published:** 2018-05-20

**Authors:** Sangeeta Kumaraswami, Suryanarayana Pothula, Mario Anthony Inchiosa, Keshar Paul Kubal, Micah Alexander Burns

**Affiliations:** ^1^Department of Anesthesiology, New York Medical College at Westchester Medical Center, 100 Woods Road, Macy Pavilion 2391, Valhalla, NY 10595, USA; ^2^Department of Pharmacology and Anesthesiology, New York Medical College at Westchester Medical Center, 100 Woods Road, Macy Pavilion 2391, Valhalla, NY 10595, USA

## Abstract

**Introduction:**

Neuraxial labor analgesia has become an integral part of modern obstetric anesthetic practice. Presence of a familiar person during its placement may be beneficial to the patient. A survey was sent to anesthesiologists practicing obstetric anesthesia in the USA to determine their views.

**Methods:**

The survey queried the following: existence of a written policy; would they allow a visitor; visitor's view, sitting or standing; reasons to allow or not allow a visitor; and influence by other staff on the decision. The responses were analyzed using multiple chi-square analyses.

**Results:**

Most practitioners supported allowing a visitor during placement. Reduction of patient anxiety and fulfillment of patient request were the major reasons for allowing a visitor. Sitting position and no view of the workspace were preferred. Visitor interference and safety were cited as the major reasons for precluding a visitor. Nonanesthesia providers rarely influenced the decision. Epidural analgesia was the preferred technique. Essentially no bias was found in the responses; there was statistical uniformity regardless of procedures done per week, years in practice, professional certification, geographic region (rural, urban, or suburban), or academic, private, or government responders.

**Conclusion:**

The practice of visitor presence during the placement of neuraxial labor analgesia is gaining acceptance.

## 1. Introduction

Over the last few decades, there have been increased in-hospital deliveries, and they account for 99% of all deliveries [1]. The need for psychological support of women in labor was recognized, and men were encouraged to provide support in the care of their partners during labor [2]. Though there was some initial opposition, this practice has become common now [3]. A pleasant birthing experience facilitates the creation of a strong bond between parents and the infant [4]. Continuous epidural anesthesia for pain management during childbirth has become a common practice in the United States of America and the rest of the world, with a significant number of women receiving neuraxial labor analgesia [5, 6].

With the involvement of anesthesiologists in vaginal deliveries in the form of labor pain management, responsibility for any visitor who is present during the procedure may belong to the anesthesiologist. No specific guidelines exist for the presence of a visitor in the room during placement of neuraxial labor analgesia. In an era where parental presence during pediatric anesthesia induction is encouraged [7], anesthesiologists may perhaps be divided in their opinions, regarding placement of neuraxial labor analgesia and presence of a visitor during the procedure.

The presence of a visitor during the placement of the neuraxial block may be desired and may benefit the parturient. It may increase patient satisfaction. There may be some disadvantages from the presence of a visitor during the procedure which may include interference and visitor safety. Anesthesiologists may have varying opinions regarding visitor presence during the placement of neuraxial labor analgesia. A survey among anesthesiologists may be useful in clarifying the concepts on this topic.

## 2. Methods

An obstetric anesthesia survey approved by the New York Medical College Institutional Review Board was designed on the SurveyMonkey® website and sent to anesthesiologists practicing obstetric anesthesia in the United States of America; no patients were involved in this study, which was strictly limited to a collection of personal responses from practicing physicians. The survey was distributed through the Society of Obstetric Anesthesia and Perinatology (SOAP), all anesthesiology residency programs, and several state anesthesia societies nonselectively. Resident physicians were not included in the study since they are unlikely to be practicing independently without supervision. To avoid duplication of responses, anesthesiologists were advised to refrain from answering the survey if they had taken it once. Participation in the survey was voluntary, and responses were completely anonymous.

SOAP is an organization of physicians who share an interest in the care of the pregnant patient and the newborn. The society sent the survey to all their members nationwide. Chairmen of the residency programs were contacted through e-mail with a request to forward the survey to physicians who practiced obstetric anesthesia in their department. State societies of all fifty states were contacted and thirty-three societies responded favorably. Those societies sent the survey to their members. The survey e-mail contained an Internet link in it, and responses were collected over a period of four months. Eighteen hundred completed responses were received. The survey first queried practitioners in relation to demographic categories. This was followed by questions related to actual clinical practice. The survey took about three to five minutes to complete.

### 2.1. Statistical analyses

We used multiple chi-square analyses (IBM-SPSS v.22 Statistical Software) to draw conclusions about the distribution of responses to the nine clinical practice-related questions of the survey. Bonferroni adjustments were applied to the critical thresholds for statistical significance (equivalent to *p* ≤ 0.05). In some instances, the two leading responses that were not statistically different from each other were combined for the statistical comparisons with the other responses (Scheffe's method).

## 3. Results

The distribution of the responses to the six questions related to the demographic categories of responding physicians are presented in Table 1.

**Figure 1:**
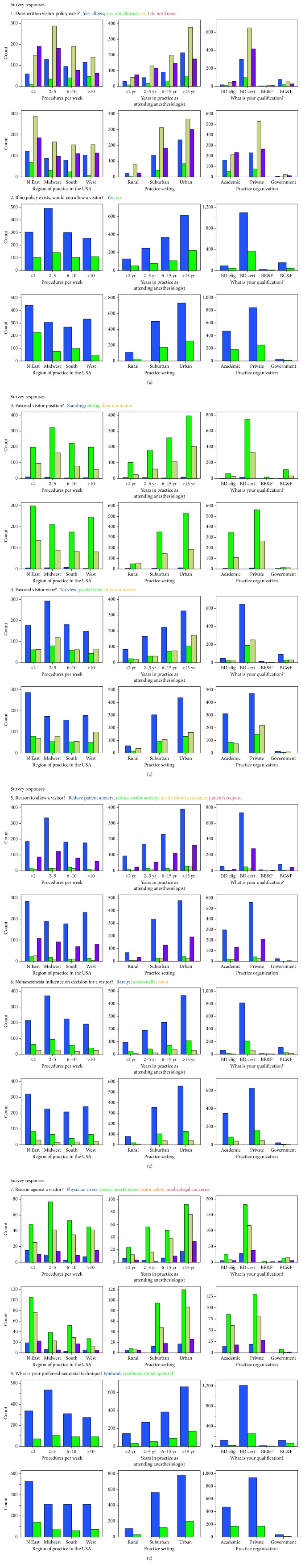
Detailed analysis of the distribution of responses to eight survey questions by the six categories of physician responders.

The detailed analysis of the responses to eight practice-related questions is presented in Figure 1.

A summary of the responses to the nine practice-related questions is as follows:

**1: Does your practice or hospital have a written policy regarding allowing a patient's visitor in the room during placement of neuraxial labor analgesia? Yes, a written policy exists—Allows visitors; Yes, a written policy exists—Does not allow visitors; No written policy exists; I do not know.**

*Physicians most knowledgeable of the policy by category were “>5 procedures/week,” “>15 years practice,” “Board certified and obstetric anesthesia fellowship trained (BC&F),” and “Rural practice.” These groups of physicians had, statistically, the smallest percentages who chose, “I do not know.”*

**2: If no policy existed, would you be open to allowing a patient's visitor in the room during placement of neuraxial labor analgesia? Yes; No.**

*All physician categories favored allowing a visitor by at least 2 : 1.*

**3: If you allowed a visitor in the room during placement of neuraxial labor analgesia, you would want the** visitor always standing; visitor always sitting; does not matter.

*For all categories, combined “sitting” and “does not matter” responses were >85%.*

**4: If you allowed a visitor in the room during placement of neuraxial labor analgesia, you would want the visitor to be positioned such that**, the visitor has no view of the procedure (the patient is between the anesthesiologist and visitor); visitor has partial view of work space (but cannot see the patient's back); does not matter.

*For all categories, “no view” was favored over either “partial view” or “does not matter” by 1.5 : 1 or greater.*

**5: What would be your single most important reason for allowing a patient's visitor in the room during placement of neuraxial labor analgesia?**

It would likely reduce patient anxiety; it would likely reduce visitor anxiety (e.g., if the visitor were very involved in patient care or if the patient were a teenager); visitor's assistance needed (e.g., if the visitor were a doula hired by the patient or if there was a language barrier); to fulfill patient request.

*For all categories, combined “reduce patient anxiety” and “to fulfill patient's request” responses were 85% or greater.*

**6: How often has another nonanesthesia member of the labor and delivery team (e.g., obstetrician or nurse) attempted to influence your decision to have a visitor present during placement of neuraxial labor analgesia?** Rarely (<5%); occasionally (5%–40%); often (>40%).

*All categories recorded “rarely” by 3 : 1 over other choices except for “Board eligible and obstetric anesthesia fellowship trained (BE&F)” which was 2 : 1.*

**7: (A similar question of frequency of a “nonanesthesia provider” to influence your decision to “not have a visitor” was also recorded as “rarely” over other choices.)**

**8: What would be your single most important reason for NOT allowing a patient's visitor in the room during placement of neuraxial labor analgesia?** Increase in anesthesiologist's stress; possible interference by the visitor (e.g., comments made if there is difficult epidural placement or if there is a medical emergency); concern about the visitor (e.g., passing out or not being able to maintain a sterile environment); medicolegal concerns.

*For all categories, combined visitor “interference” and “concern” responses were 80% or greater.*

**9: What is your preferred technique for neuraxial analgesia in an otherwise healthy parturient?** Epidural analgesia; combined spinal-epidural analgesia.

*All categories chose “Epidural analgesia” by 1.8 : 1 or greater except for “Qualification,” and the “Board eligible and obstetric anesthesia fellowship trained (BE&F)” chose both options equally.*

## 4. Discussion

Most of the anesthesiologists in our survey were agreeable to the presence of a visitor in the room while doing the neuraxial labor analgesia procedure. The preferences of patients and their expectations of physicians can change with times. The parturient may like to have a familiar person in the room to reassure her while undergoing the neuraxial labor analgesia procedure. This person can be the husband, a partner, a friend, a family member, or even a nonmedical semiprofessional person like a doula. This may reflect a changing social environment. The specialty of anesthesiology may be adapting positively to those changes.

Of our respondents, 29.3% reported that their institutions had written policies regarding either specifically allowing or not allowing a visitor during the procedure. Any policy regarding the presence of a visitor should be written in detail with input from the department of risk management. It should preferably include a statement to remind staff members that they must use their judgement and individualize each decision. The presence of the spouse or a family member during placement of the neuraxial block should be discussed as part of the anesthetic plan. While the institution can have a policy, the final decision should be that of the anesthesiologist [8]. It may be preferable to mention visitor presence and possible disadvantages in the informed consent.

If a visitor was allowed in the room, then visitor “sitting” or “does not matter” with “no view” of workspace was preferred by most anesthesiologists. Sitting on a chair would minimize the risk of fainting and falling on the floor. Visitors involved with the care of a parturient might be sleep-deprived or hypoglycemic. Vasovagal syncope might be an added factor. Standing would only increase their risk of falling. Having no view of the workspace while the procedure was being done was preferred by many practitioners. This would mean that the visitor's position offered no view of the procedural field. Looking at an epidural needle may likely scare a layperson. Moreover, a potentially difficult or bloody placement of the epidural needle may lead to a messy procedural tray. A view of this, though perhaps commonplace for a physician, might be upsetting to a visitor.

According to the survey findings, important reasons for having a visitor in the room appeared to be to “reduce patient's anxiety” and “fulfill patient's request.” Reduction in patient's anxiety may be thought of as being obvious if a familiar person is present in the room while the procedure is being performed. Contrary to expectations, a partner's presence during epidural catheter insertion for labor analgesia did not always decrease anxiety levels, instead anxiety and pain of epidural catheter placement were greater if the partner remained in the room [9]. Partner's presence can be associated with a decrease in the mother's pain memory with no reduction in her anxiety [10]. Similar results have been reported in the pediatric literature. Contrary to popular belief, parental presence did not always alleviate parents' or children's anxiety [11]. Not all patients may want a visitor in the room during epidural placement. There can be cultural influences and variations involved in deciding whether the partner is present in the delivery room during childbirth [12–14]. The partner's presence in the operating room during neuraxial anesthesia for cesarean delivery was associated with no significant difference in patient anxiety but was associated with decreased partner anxiety [15]. Epidural analgesia reduced paternal anxiety and stress and increased paternal involvement, participation, and satisfaction with the experience of childbirth [16]. An expectant mother may be helped emotionally by the presence of the partner, especially if there is a language barrier between the delivery team and the patient. This can be one of the prominent reasons for allowing a visitor during the placement of the epidural block. In view of these variations, it is scientifically prudent to conduct a new prospective study to clarify confusing observations regarding patient anxiety.

The influence by the nonanesthesiology members of the labor and delivery team in our decision making was found to be predominantly “rarely.” This would be consistent with the statement endorsed by the American Congress of Obstetricians and Gynecologists and the Association of Women's Health, Obstetric and Neonatal Nurses, which states that optimal maternal and fetal outcomes can best be achieved in an atmosphere of multidisciplinary collaboration [17]. This indicates confidence in the decision-making capabilities of the anesthesiologists among the labor and delivery team.

“Interference by the visitor” and “concern about the visitor” were the leading causes for not wanting to have a visitor in the room. It is possible that one may encounter a disruptive visitor, which would be counterproductive to the care of the patient, especially if the placement of the block is difficult. Visitors in an operating room during a cesarean section made it more difficult to care for the patient with risks outweighing benefits [18]. Concerns about visitors include potential for fainting and compromise of sterility [19, 20]. Injuries to the visitor can occur, and the visitor may need to go to the emergency room for further medical care. Such injuries may lead to medicolegal complications [21]. A safety concern has been raised about nurses leaving the room during placement when there is an increase in patient census in the labor and delivery suite, which may result in the visitor helping with patient positioning [22]. Contamination of a sterile field is a concern. Respiratory droplets are known to travel three to four feet. It is recommended that visitors within four feet of the patient or procedural tray wear a mask during a neuraxial procedure. Wearing surgical masks would decrease sterile field contamination with nasal and oropharyngeal commensal bacteria [23, 24]. Allowing a visitor in the room while placing an epidural block may encourage the visitor to photograph or videotape the procedure. This may interfere with the medical procedure. The hospital policies regarding photography and videography should be ascertained and followed. This may involve medicolegal and privacy concerns.

Most anesthesiologists chose epidural over combined spinal-epidural analgesia as a preferred technique for labor analgesia. There are varied opinions about the practice of combined spinal-epidural versus epidural analgesia in labor [25]. Combined spinal-epidural analgesia (CSE) is considered superior and faster than epidural in the first but not in the second stage of labor [26]. It was of interest that most anesthesiologists preferred epidural rather than the combined technique. However, the “board eligible and obstetric anesthesia fellowship trained” anesthesiologists chose both techniques equally. It may be that the older generation of obstetric anesthesiologists were following the technique they were trained in during their residency, and younger recently trained members of the community are willing to explore both newer and traditional methods. If this assumption is true, this choice may change in the future.

One limitation of our survey is that we do not know the response rate among those who were invited to participate. We sacrificed the ability to determine the exact number of individuals who were successfully contacted by SOAP, departmental chairmen, or professional anesthesia state societies in favor of obtaining as large a response as possible.

The results of the survey strongly suggest that there is essentially no bias in the responses to the questions among the several categories of physicians; there is a marked statistical uniformity of responses from physicians regardless of procedures done per week, years in practice, professional certification, geographic region, practice setting (rural, urban, or suburban), or organization (academic, private, or government) (Figure 1).

## 5. Conclusion

The ASA task force on obstetric anesthesia guidelines supports a multidisciplinary approach to create favorable maternal and fetal outcomes [27]. Our research study was done to determine attitudes and opinions of obstetric anesthesiologists towards visitor presence. The practice of visitor presence during placement of neuraxial labor analgesia appears to be gaining acceptance with anesthesiologists. Patient's rights, visitor concerns, benefits, and risks should be weighed before a decision is made. Limited literature exists on this subject in obstetric anesthesia. Further studies need to be done to ascertain the advantages and disadvantages of this practice.

## Figures and Tables

**Table 1:** Survey demographics (categories of physicians).

Questions	Physician category	Number (%)
Neuraxial procedures/week	<2	405 (22.50)
	2–5	632 (35.11)
	6–10	400 (22.22)
	>10	363 (20.16)

Years in practice	<2	175 (9.72)
	2–5	323 (17.94)
	6–15	472 (26.22)
	>15	830 (46.11)

Qualification	Board eligible	130 (7.22)
	Board certified	1463 (81.27)
	BE&F	25 (1.38)
	BC&F	182 (10.11)

Region of practice	Northeast	666 (37.0)
	Midwest	384 (21.33)
	South	369 (20.50)
	West	381 (21.16)

Practice setting	Rural	134 (7.44)
	Suburban	679 (37.72)
	Urban	987 (54.83)

Nature of practice	Academic	658 (36.55)
	Private	1100 (61.11)
	Government	42 (2.33)

BE&F, board eligible and obstetric anesthesia fellowship trained; BC&F, board certified and obstetric anesthesia fellowship trained.

## Supplementary Materials

Supplementary Materials This supplement provides the detailed summary of the data from the survey that were filtered and collated to provide the responses to each practice question by each of the six categories of physicians.
