# Supplementary material for: Anesthesiologists' Preferences regarding Visitor Presence during Placement of Neuraxial Labor Analgesia
Source: Anesthesiol Res Pract. 2018 May 20;2018:3481975. doi: 10.1155/2018/3481975 (PMC5985117; doi:10.1155/2018/3481975)
Supplement: Supplementary Materials — This supplement provides the detailed summary of the data from the survey that were filtered and collated to provide the responses to each practice question by each of the six categories of physicians. [file 3481975.f1.pdf]

Notes

Output Created		20-JAN-2016 21:40:42
Comments		
Input	Data	/Users/Keshar/Desktop/THUMBDRIVE/SURVEY MONKEY/TotalData.sav
	Active Dataset	DataSet1
	Filter	<none>
	Weight	<none>
	Split File	<none>
	N of Rows in Working Data File	1302
Missing Value Handling	Definition of Missing	User-defined missing values are treated as missing.
	Cases Used	Statistics for each table are based on all the cases with valid data in the specified range(s) for all variables in each table.
Syntax		CROSSTABS /TABLES=NeuraxialProcPerWeek AttendingYears Qualification Region Setting Nature BY PrefTechnique PtInRoomPolicy NoPolicyAllowVisitor VisitorStandingSitting VisitorNoViewView ReasonForVisitor OutsideInfluenceFOR OutsideInfluenceAGAINST ReasonForNOTAllowingVisitor /FORMAT=AVALUE TABLES /STATISTICS=CHISQ /CELLS=COUNT ROW ASRESID /COUNT ROUND CELL /BARCHART.
Resources	Processor Time	00:00:08.12
	Elapsed Time	00:00:08.00
	Dimensions Requested	2
	Cells Available	131029

On an average, how many neuraxial procedures for labor analgesia do you perform per week? * What is your preferred technique for neuraxial labor analgesia in an otherwise healthy parturient?

Crosstab

			What is your preferred technique for neuraxial labor analgesia in an otherwise healthy parturient?		
			Epidural analgesia	Combined spinal epidural analgesia	Total
On an average, how many neuraxial procedures for labor analgesia do you perform per week?	< 2	Count	244	60	304
		% within On an average, how many neuraxial procedures for labor analgesia do you perform per week?	80.3%	19.7%	100.0%
		Adjusted Residual	1.1	-1.1	
	2- 5	Count	356	76	432
		% within On an average, how many neuraxial procedures for labor analgesia do you perform per week?	82.4%	17.6%	100.0%
		Adjusted Residual	2.7	-2.7	
	6-10	Count	207	71	278
		% within On an average, how many neuraxial procedures for labor analgesia do you perform per week?	74.5%	25.5%	100.0%
		Adjusted Residual	-1.6	1.6	

Crosstab

		What is your preferred technique for neuraxial labor analgesia in an otherwise healthy parturient?		Total
		Epidural analgesia	Combined spinal epidural analgesia	
>10	Count	208	79	287
	% within On an average, how many neuraxial procedures for labor analgesia do you perform per week?	72.5%	27.5%	100.0%
	Adjusted Residual	-2.6	2.6	
Total	Count	1015	286	1301
	% within On an average, how many neuraxial procedures for labor analgesia do you perform per week?	78.0%	22.0%	100.0%

Chi-Square Tests

	Value	df	Asymp. Sig. (2-sided)
Pearson Chi-Square	12.942[a]	3	.005
Likelihood Ratio	12.881	3	.005
Linear-by-Linear Association	9.154	1	.002
N of Valid Cases	1301		

a. 0 cells (0.0%) have expected count less than 5. The minimum expected count is 61.11.

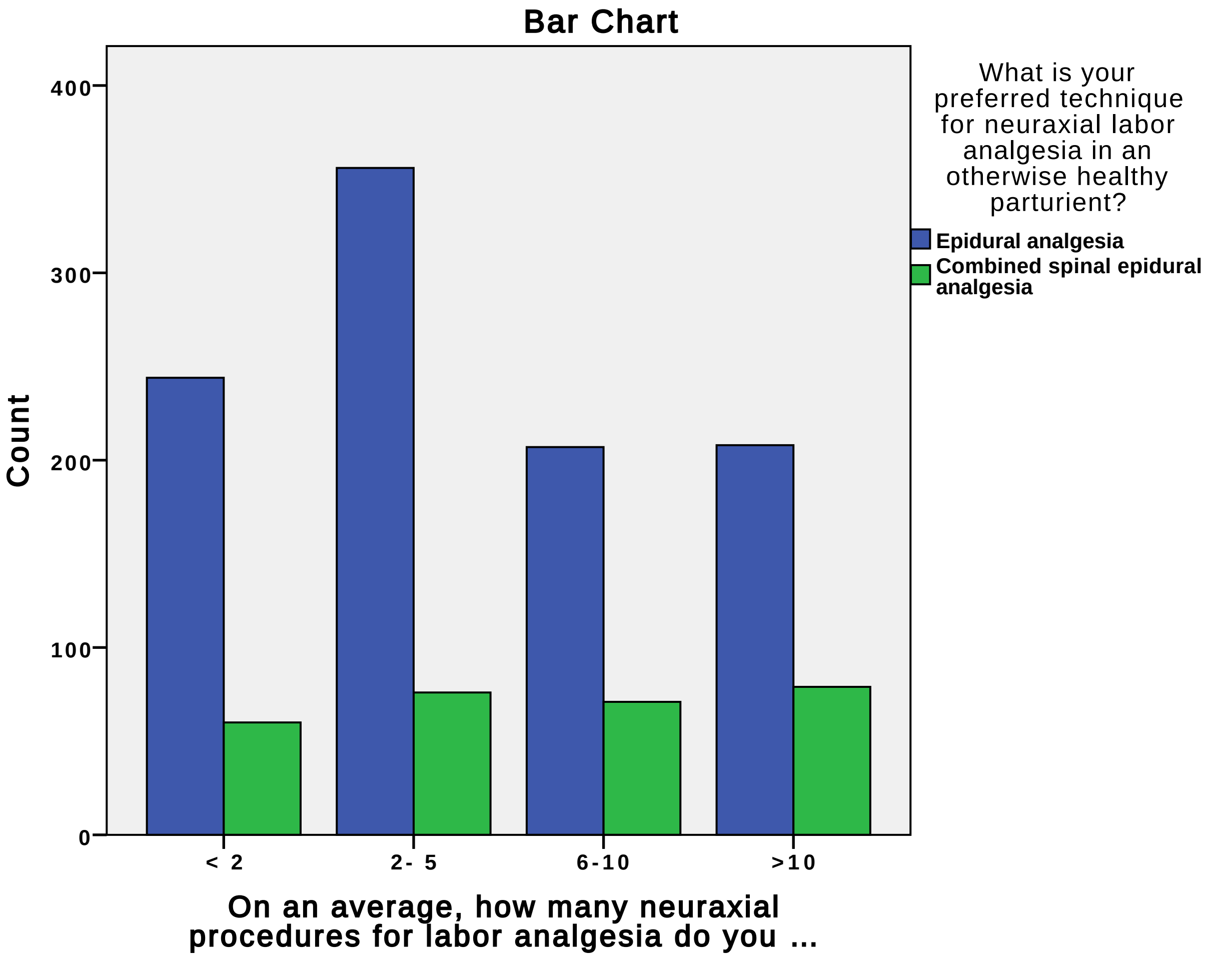

On an average, how many neuraxial procedures for labor analgesia do you perform per week? * Does your practice or hospital have a written policy regarding allowing a patient’s visitor in the room during placement of neuraxial labor analgesia?

Crosstab

			Does your practice or hospital have a written policy regarding allowing a patient's visitor in the room during placement of neuraxial labor ..		
			Yes, a written policy exists - Allows visitors	Yes, a written policy exists - Does not allow visitors	No written policy exists
On an average, how many neuraxial procedures for labor analgesia do you perform per week?	< 2	Count	35	9	108
		% within On an average, how many neuraxial procedures for labor analgesia do you perform per week?	11.5%	3.0%	35.5%
		Adjusted Residual	-5.2	-3.7	-1.6
	2- 5	Count	93	26	176
		% within On an average, how many neuraxial procedures for labor analgesia do you perform per week?	21.5%	6.0%	40.7%
		Adjusted Residual	-.6	-1.9	.7
	6-10	Count	70	29	126
		% within On an average, how many neuraxial procedures for labor analgesia do you perform per week?	25.2%	10.4%	45.3%
		Adjusted Residual	1.2	1.6	2.3

Crosstab

			Does your practice or hospital have a ...	
			I do not know	Total
On an average, how many neuraxial procedures for labor analgesia do you perform per week?	< 2	Count	152	304
		% within On an average, how many neuraxial procedures for labor analgesia do you perform per week?	50.0%	100.0%
		Adjusted Residual	8.7	
	2- 5	Count	137	432
		% within On an average, how many neuraxial procedures for labor analgesia do you perform per week?	31.7%	100.0%
		Adjusted Residual	1.0	
	6-10	Count	53	278
		% within On an average, how many neuraxial procedures for labor analgesia do you perform per week?	19.1%	100.0%
		Adjusted Residual	-4.5	

Crosstab

		Does your practice or hospital have a written policy regarding allowing a patient's visitor in the room during placement of neuraxial labor ...		
		Yes, a written policy exists - Allows visitors	Yes, a written policy exists - Does not allow visitors	No written policy exists
>10	Count	95	41	103
	% within On an average, how many neuraxial procedures for labor analgesia do you perform per week?	33.1%	14.3%	35.9%
	Adjusted Residual	4.9	4.4	-1.4
Total	Count	293	105	513
	% within On an average, how many neuraxial procedures for labor analgesia do you perform per week?	22.5%	8.1%	39.4%

Crosstab

		Does your practice or hospital have a ...	
		I do not know	Total
>10	Count	48	287
	% within On an average, how many neuraxial procedures for labor analgesia do you perform per week?	16.7%	100.0%
	Adjusted Residual	-5.6	
Total	Count	390	1301
	% within On an average, how many neuraxial procedures for labor analgesia do you perform per week?	30.0%	100.0%

Chi-Square Tests

	Value	df	Asymp. Sig. (2-sided)
Pearson Chi-Square	133.104[a]	9	.000
Likelihood Ratio	133.468	9	.000
Linear-by-Linear Association	99.543	1	.000
N of Valid Cases	1301		

a. 0 cells (0.0%) have expected count less than 5. The minimum expected count is 22.44.

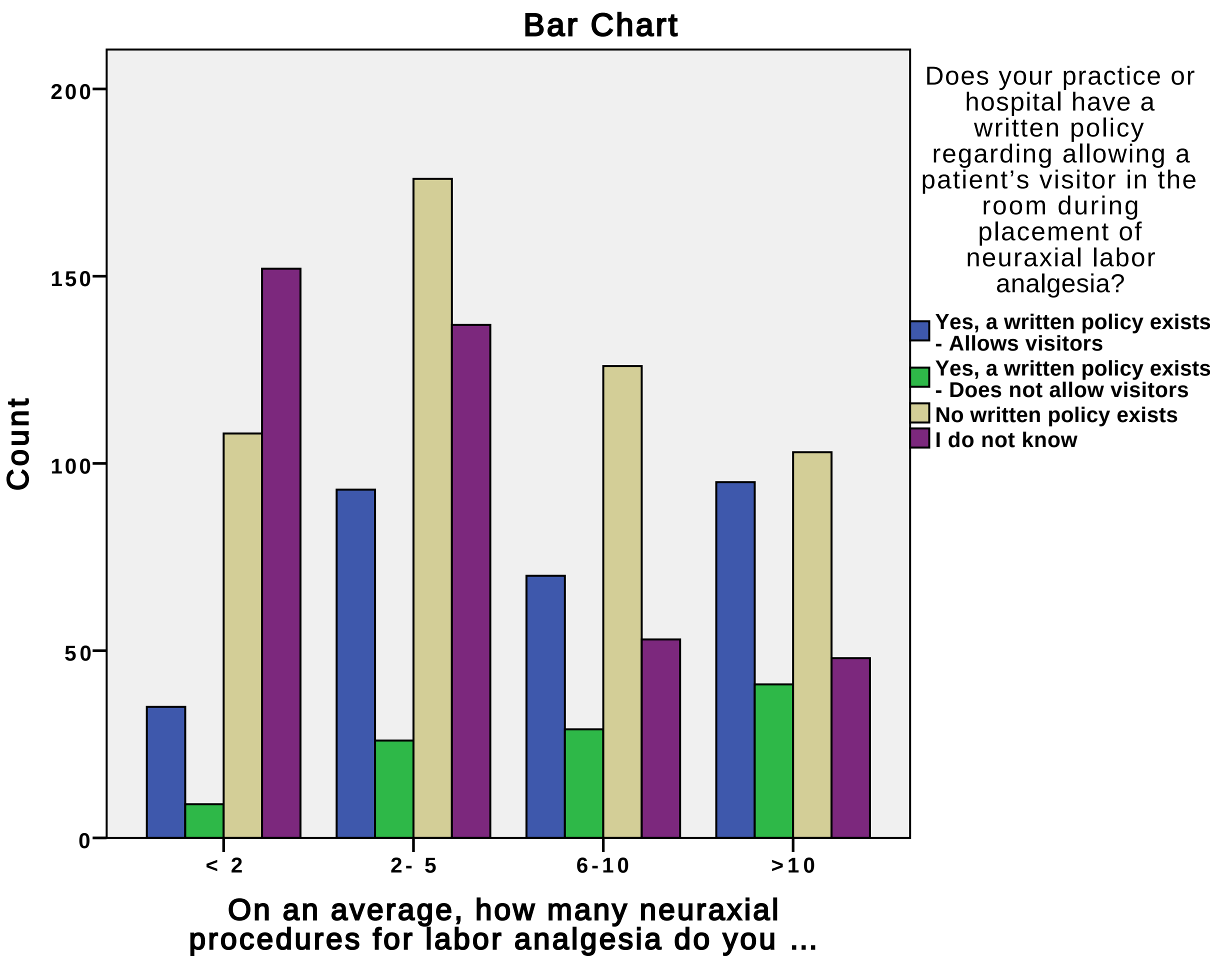

On an average, how many neuraxial procedures for labor analges ia do you perform per week? * If no policy existed, would you be open to allowing a patient’s visitor in the room during placement of neuraxial labor analgesia?

Crosstab

			If no policy existed, would you be open to allowing a patient's visitor in the room during placement of neuraxial labor analgesia?		
			Yes	No	Total
On an average, how many neuraxial procedures for labor analgesia do you perform per week?	< 2	Count	225	79	304
		% within On an average, how many neuraxial procedures for labor analgesia do you perform per week?	74.0%	26.0%	100.0%
		Adjusted Residual	-.5	.5	
	2- 5	Count	345	87	432
		% within On an average, how many neuraxial procedures for labor analgesia do you perform per week?	79.9%	20.1%	100.0%
		Adjusted Residual	2.8	-2.8	
	6-10	Count	207	71	278
		% within On an average, how many neuraxial procedures for labor analgesia do you perform per week?	74.5%	25.5%	100.0%
		Adjusted Residual	-.3	.3	

Crosstab

		If no policy existed, would you be open to allowing a patient's visitor in the room during placement of neuraxial labor analgesia?		Total
		Yes	No	
>10	Count	200	87	287
	% within On an average, how many neuraxial procedures for labor analgesia do you perform per week?	69.7%	30.3%	100.0%
	Adjusted Residual	-2.4	2.4	
Total	Count	977	324	1301
	% within On an average, how many neuraxial procedures for labor analgesia do you perform per week?	75.1%	24.9%	100.0%

Chi-Square Tests

	Value	df	Asymp. Sig. (2-sided)
Pearson Chi-Square	9.986[a]	3	.019
Likelihood Ratio	10.043	3	.018
Linear-by-Linear Association	3.107	1	.078
N of Valid Cases	1301		

a. 0 cells (0.0%) have expected count less than 5. The minimum expected count is 69.23.

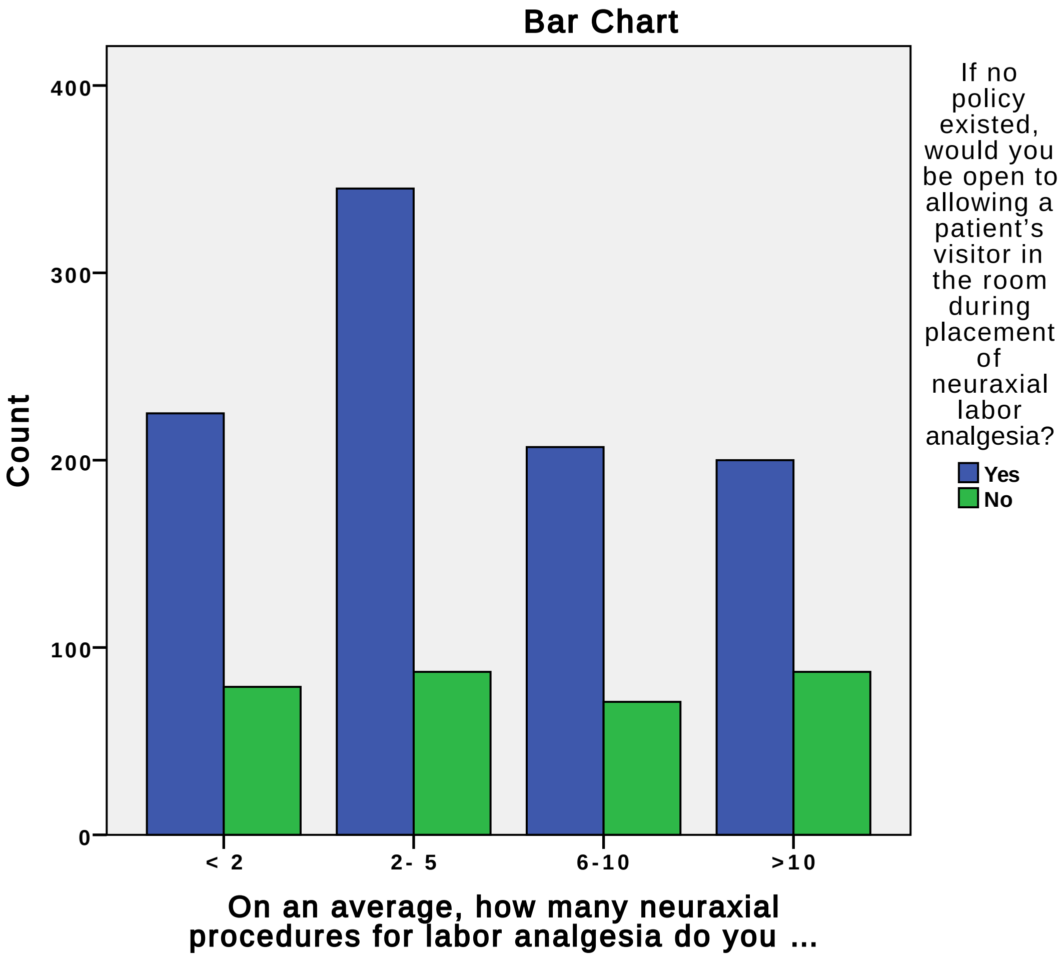

On an average, how many neuraxial procedures for labor analgesia do you perform per week? * If you allowed a visitor in the room during placement of neuraxial labor analgesia, you would want the

Crosstab

			If you allowed a visitor in the room during placement of neuraxial labor analgesia, you would want the		
			Visitor always standing	Visitor always sitting	Does not matter
On an average, how many neuraxial procedures for labor analgesia do you perform per week?	< 2	Count	7	149	68
		% within On an average, how many neuraxial procedures for labor analgesia do you perform per week?	3.1%	66.5%	30.4%
		Adjusted Residual	2.4	-2.1	1.5
	2- 5	Count	2	241	99
		% within On an average, how many neuraxial procedures for labor analgesia do you perform per week?	0.6%	70.5%	28.9%
		Adjusted Residual	-1.6	-.8	1.3
	6-10	Count	2	155	49
		% within On an average, how many neuraxial procedures for labor analgesia do you perform per week?	1.0%	75.2%	23.8%
		Adjusted Residual	-.6	1.2	-1.0

Crosstab

			Total
On an average, how many neuraxial procedures for labor analgesia do you perform per week?	< 2	Count	224
		% within On an average, how many neuraxial procedures for labor analgesia do you perform per week?	100.0%
		Adjusted Residual	
	2- 5	Count	342
		% within On an average, how many neuraxial procedures for labor analgesia do you perform per week?	100.0%
		Adjusted Residual	
	6-10	Count	206
		% within On an average, how many neuraxial procedures for labor analgesia do you perform per week?	100.0%
		Adjusted Residual	

Crosstab

		If you allowed a visitor in the room during placement of neuraxial labor analgesia, you would want the		
		Visitor always standing	Visitor always sitting	Does not matter
>10	Count	3	156	42
	% within On an average, how many neuraxial procedures for labor analgesia do you perform per week?	1.5%	77.6%	20.9%
	Adjusted Residual	.1	2.0	-2.0
Total	Count	14	701	258
	% within On an average, how many neuraxial procedures for labor analgesia do you perform per week?	1.4%	72.0%	26.5%

Crosstab

		Total
>10	Count	201
	% within On an average, how many neuraxial procedures for labor analgesia do you perform per week?	100.0%
	Adjusted Residual	
Total	Count	973
	% within On an average, how many neuraxial procedures for labor analgesia do you perform per week?	100.0%

Chi-Square Tests

	Value	df	Asymp. Sig. (2-sided)
Pearson Chi-Square	13.685[a]	6	.033
Likelihood Ratio	13.243	6	.039
Linear-by-Linear Association	4.443	1	.035
N of Valid Cases	973		

a. 4 cells (33.3%) have expected count less than 5. The minimum expected count is 2.89.

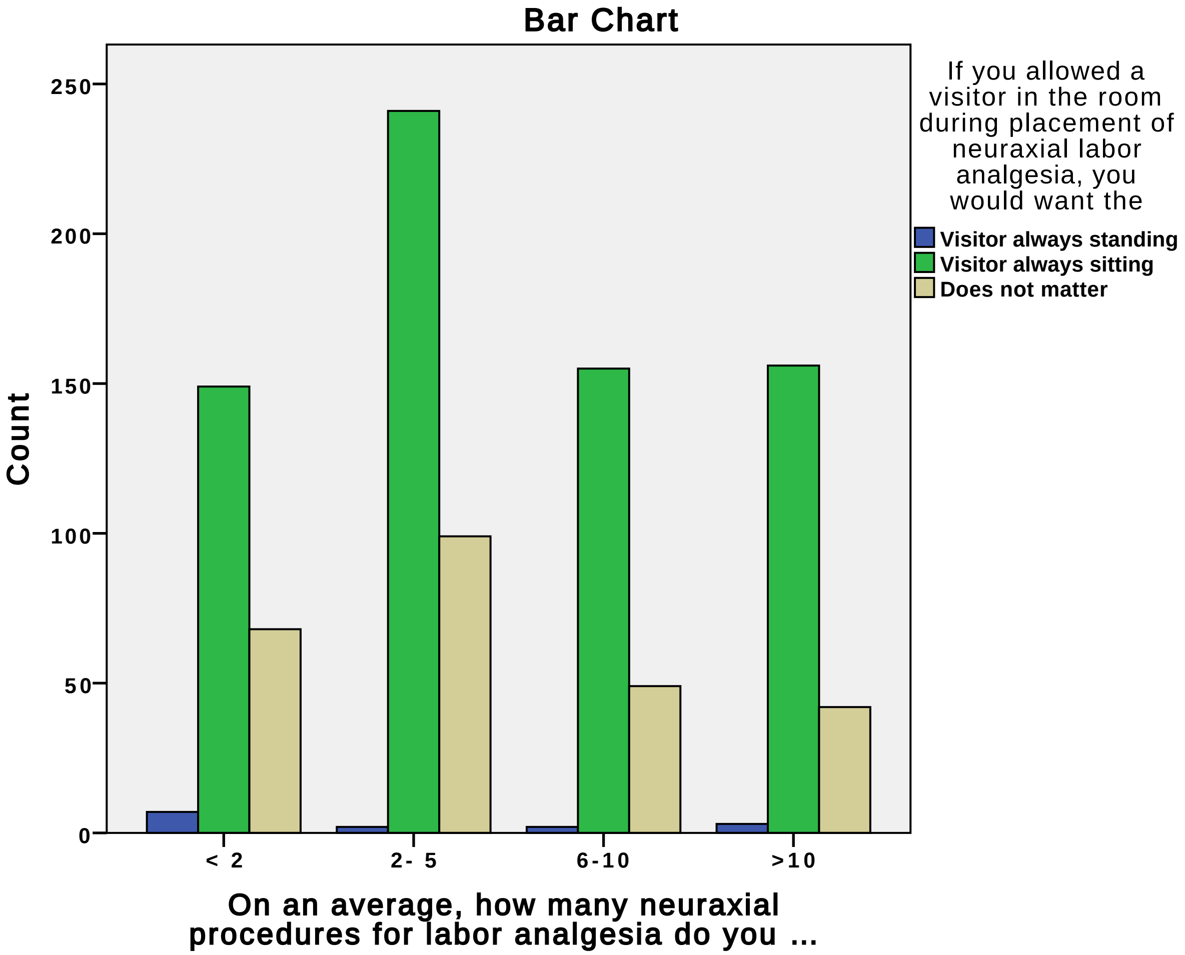

On an average, how many neuraxial procedures for labor analgesia do you perform per week? * If you allowed a visitor in the room during placement of neuraxial labor analgesia, you would want the visitor to be positioned such that,

Crosstab

			If you allowed a visitor in the room during placement of neuraxial labor analgesia, you would want the visitor to be positioned such that,		
			Visitor has no view of procedure (patient is in between anes	Visitor has partial view of workspace (but cannot see patien	Does not matter
On an average, how many neuraxial procedures for labor analgesia do you perform per week?	< 2	Count	132	47	45
		% within On an average, how many neuraxial procedures for labor analgesia do you perform per week?	58.9%	21.0%	20.1%
		Adjusted Residual	-.7	.9	.1
	2- 5	Count	211	53	78
		% within On an average, how many neuraxial procedures for labor analgesia do you perform per week?	61.7%	15.5%	22.8%
		Adjusted Residual	.3	-2.1	1.6
	6-10	Count	129	47	30
		% within On an average, how many neuraxial procedures for labor analgesia do you perform per week?	62.6%	22.8%	14.6%
		Adjusted Residual	.5	1.6	-2.2

Crosstab

			Total
On an average, how many neuraxial procedures for labor analgesia do you perform per week?	< 2	Count	224
		% within On an average, how many neuraxial procedures for labor analgesia do you perform per week?	100.0%
		Adjusted Residual	
	2- 5	Count	342
		% within On an average, how many neuraxial procedures for labor analgesia do you perform per week?	100.0%
		Adjusted Residual	
	6-10	Count	206
		% within On an average, how many neuraxial procedures for labor analgesia do you perform per week?	100.0%
		Adjusted Residual	

Crosstab

		If you allowed a visitor in the room during placement of neuraxial labor analgesia, you would want the visitor to be positioned such that,		
		Visitor has no view of procedure (patient is in between anes	Visitor has partial view of workspace (but cannot see patien	Does not matter
>10	Count	122	38	41
	% within On an average, how many neuraxial procedures for labor analgesia do you perform per week?	60.7%	18.9%	20.4%
	Adjusted Residual	-.1	.0	.2
Total	Count	594	185	194
	% within On an average, how many neuraxial procedures for labor analgesia do you perform per week?	61.0%	19.0%	19.9%

Crosstab

			Total
	>10	Count	201
		% within On an average, how many neuraxial procedures for labor analgesia do you perform per week?	100.0%
		Adjusted Residual	
Total		Count	973
		% within On an average, how many neuraxial procedures for labor analgesia do you perform per week?	100.0%

Chi-Square Tests

	Value	df	Asymp. Sig. (2-sided)
Pearson Chi-Square	8.945[a]	6	.177
Likelihood Ratio	9.235	6	.161
Linear-by-Linear Association	.375	1	.540
N of Valid Cases	973		

a. 0 cells (0.0%) have expected count less than 5. The minimum expected count is 38.22.

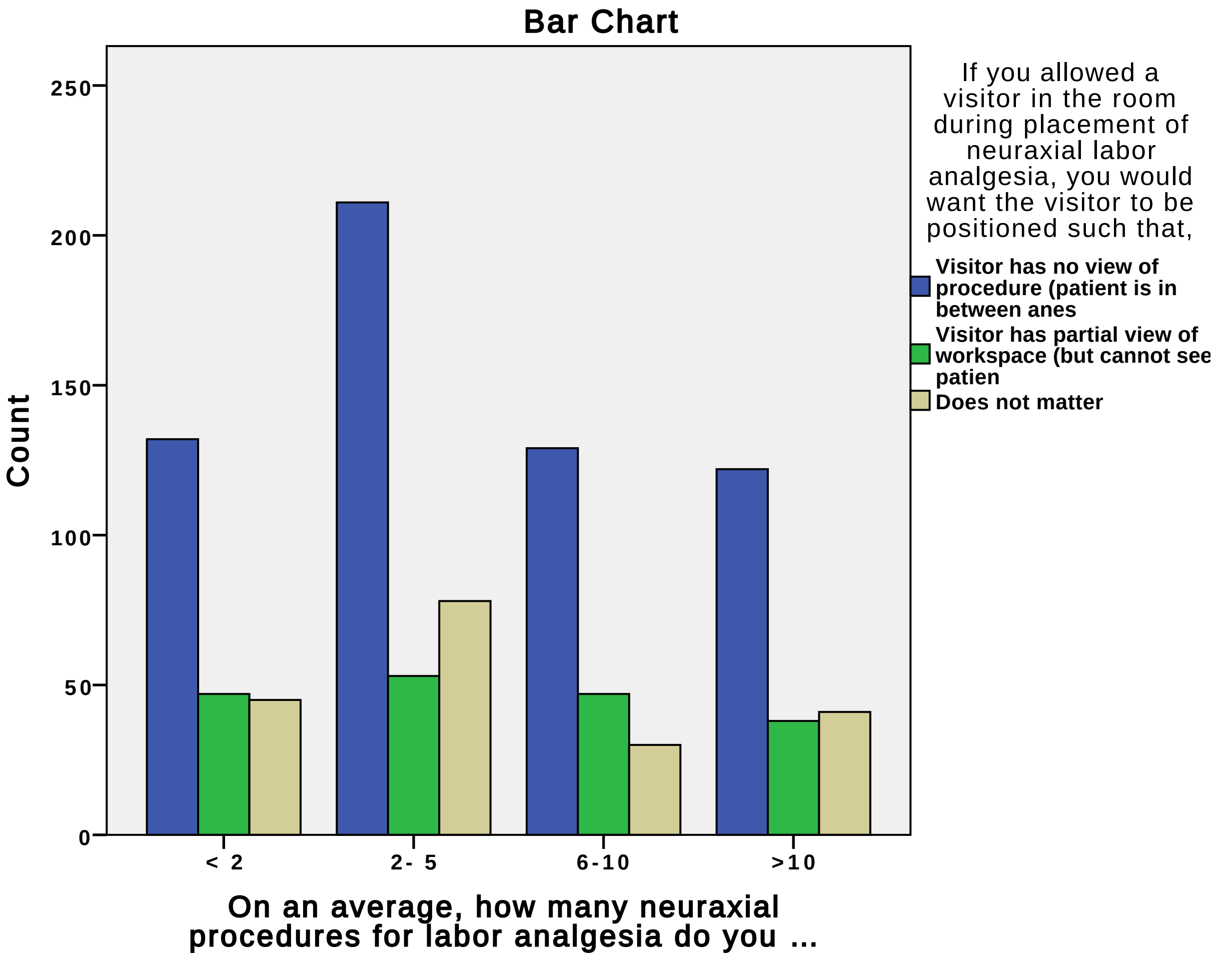

On an average, how many neuraxial procedures for labor analges ia do you perform per week? * What would be your single most i mportant reason for allowing a patient’s visitor in the room durin g placement of neuraxial labor analgesia?

Crosstab

			What would be your single most important reason for allowing a patient's visitor in the room during placement of neuraxial labor ...		
			It would likely reduce patient's anxiety	It would likely reduce visitor's anxiety (for e. g. if visito	Visitor's assistance needed (for e. g. if visitor were a doul
On an average, how many neuraxial procedures for labor analgesia do you perform per week?	< 2	Count	141	14	8
		% within On an average, how many neuraxial procedures for labor analgesia do you perform per week?	62.9%	6.3%	3.6%
		Adjusted Residual	-.6	.9	-.2
	2- 5	Count	232	11	10
		% within On an average, how many neuraxial procedures for labor analgesia do you perform per week?	67.8%	3.2%	2.9%
		Adjusted Residual	1.5	-1.9	-1.1
	6-10	Count	121	19	9
		% within On an average, how many neuraxial procedures for labor analgesia do you perform per week?	58.7%	9.2%	4.4%
		Adjusted Residual	-2.0	3.1	.5

Crosstab

			What would be your single most important ...	
			To fulfill patient's request	Total
On an average, how many neuraxial procedures for labor analgesia do you perform per week?	< 2	Count	61	224
		% within On an average, how many neuraxial procedures for labor analgesia do you perform per week?	27.2%	100.0%
		Adjusted Residual	.3	
	2- 5	Count	89	342
		% within On an average, how many neuraxial procedures for labor analgesia do you perform per week?	26.0%	100.0%
		Adjusted Residual	-.3	
	6-10	Count	57	206
		% within On an average, how many neuraxial procedures for labor analgesia do you perform per week?	27.7%	100.0%
		Adjusted Residual	.4	

Crosstab

		What would be your single most important reason for allowing a patient’s visitor in the room during placement of neuraxial labor …		
		It would likely reduce patient's anxiety	It would likely reduce visitor's anxiety (for e. g. if visito	Visitor's assistance needed (for e. g. if visitor were a doul
>10	Count	135	5	10
	% within On an average, how many neuraxial procedures for labor analgesia do you perform per week?	67.2%	2.5%	5.0%
	Adjusted Residual	.8	-1.9	1.0
Total	Count	629	49	37
	% within On an average, how many neuraxial procedures for labor analgesia do you perform per week?	64.6%	5.0%	3.8%

Crosstab

		What would be your single most important …	
		To fulfill patient's request	Total
>10	Count	51	201
	% within On an average, how many neuraxial procedures for labor analgesia do you perform per week?	25.4%	100.0%
	Adjusted Residual	-.4	
Total	Count	258	973
	% within On an average, how many neuraxial procedures for labor analgesia do you perform per week?	26.5%	100.0%

Chi-Square Tests

	Value	df	Asymp. Sig. (2-sided)
Pearson Chi-Square	16.520[a]	9	.057
Likelihood Ratio	15.990	9	.067
Linear-by-Linear Association	.005	1	.945
N of Valid Cases	973		

a. 0 cells (0.0%) have expected count less than 5. The minimum expected count is 7.64.

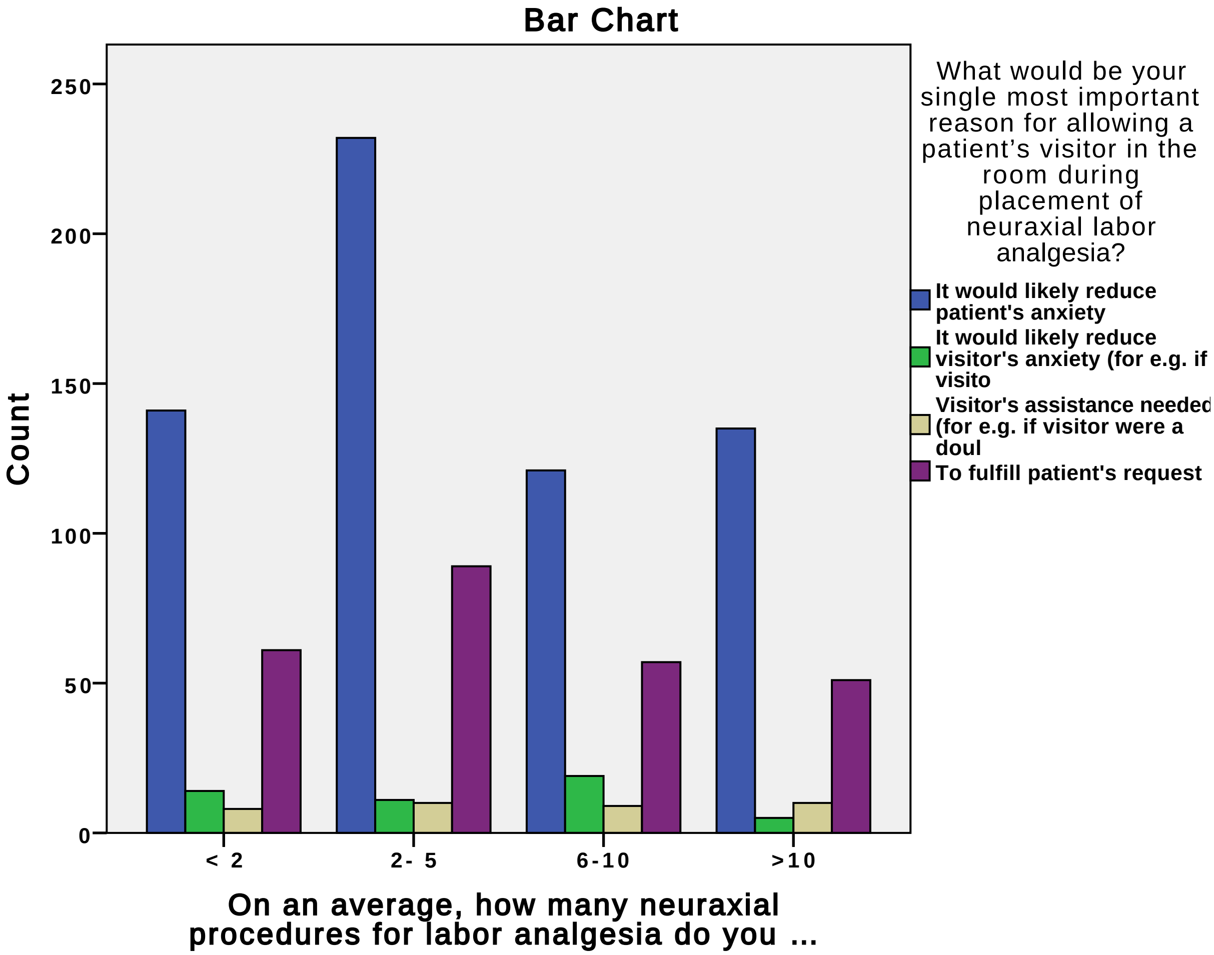

On an average, how many neuraxial procedures for labor analgesia do you perform per week? * How often has another non anesthesia member of the labor and delivery team (for example obstetrician or nurse) attempted to influence your decision to have a visitor present during placement of neuraxial labor analgesia?

Crosstab

			How often has another non anesthesia member of the labor and delivery team (for example obstetrician or nurse) attempted to influence your decision to have a visitor present during placement of neuraxial labor analgesia?		
			Rarely (< 5 %)	Occasionally (5 % - 40 %)	Often > 40 %
On an average, how many neuraxial procedures for labor analgesia do you perform per week?	< 2	Count	160	43	21
		% within On an average, how many neuraxial procedures for labor analgesia do you perform per week?	71.4%	19.2%	9.4%
		Adjusted Residual	-.9	.5	.7
	2- 5	Count	263	57	22
		% within On an average, how many neuraxial procedures for labor analgesia do you perform per week?	76.9%	16.7%	6.4%
		Adjusted Residual	1.7	-.8	-1.5
	6-10	Count	149	42	15
		% within On an average, how many neuraxial procedures for labor analgesia do you perform per week?	72.3%	20.4%	7.3%
		Adjusted Residual	-.5	1.0	-.6

Crosstab

			Total
On an average, how many neuraxial procedures for labor analgesia do you perform per week?	< 2	Count	224
		% within On an average, how many neuraxial procedures for labor analgesia do you perform per week?	100.0%
		Adjusted Residual	
	2- 5	Count	342
		% within On an average, how many neuraxial procedures for labor analgesia do you perform per week?	100.0%
		Adjusted Residual	
	6-10	Count	206
		% within On an average, how many neuraxial procedures for labor analgesia do you perform per week?	100.0%
		Adjusted Residual	

Crosstab

		How often has another non anesthesia member of the labor and delivery team (for example obstetrician or nurse) attempted to influence your decision to have a visitor present during placement of neuraxial labor analgesia?		
		Rarely (< 5 %)	Occasionally (5 % - 40 %)	Often > 40 %
>10	Count	145	34	22
	% within On an average, how many neuraxial procedures for labor analgesia do you perform per week?	72.1%	16.9%	10.9%
	Adjusted Residual	-.6	-.5	1.6
Total	Count	717	176	80
	% within On an average, how many neuraxial procedures for labor analgesia do you perform per week?	73.7%	18.1%	8.2%

Crosstab

		Total
>10	Count	201
	% within On an average, how many neuraxial procedures for labor analgesia do you perform per week?	100.0%
	Adjusted Residual	
Total	Count	973
	% within On an average, how many neuraxial procedures for labor analgesia do you perform per week?	100.0%

Chi-Square Tests

	Value	df	Asymp. Sig. (2-sided)
Pearson Chi-Square	5.769[a]	6	.450
Likelihood Ratio	5.681	6	.460
Linear-by-Linear Association	.255	1	.613
N of Valid Cases	973		

a. 0 cells (0.0%) have expected count less than 5. The minimum expected count is 16.53.

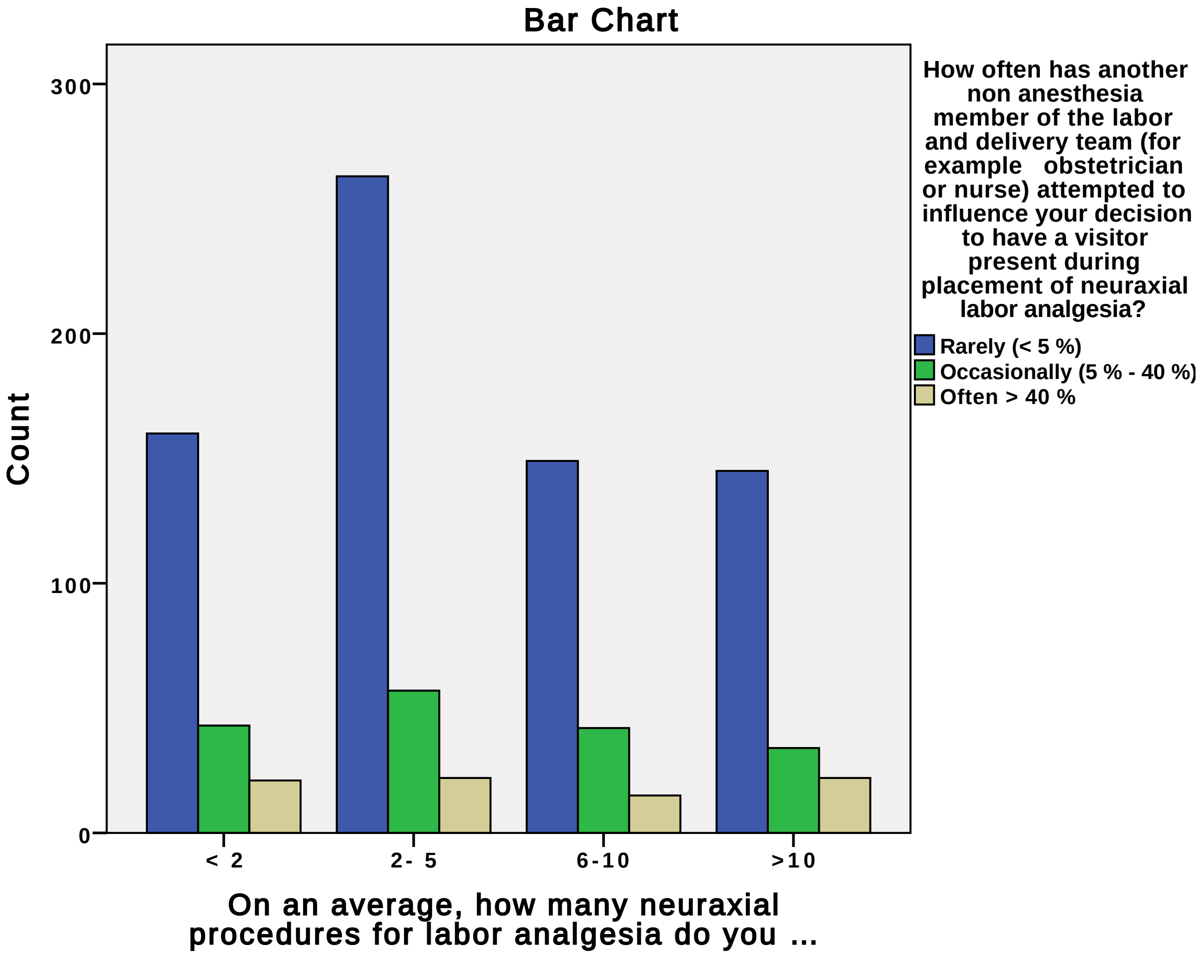

On an average, how many neuraxial procedures for labor analges ia do you perform per week? * How often has another non anesth esia member of the labor and delivery team (for example obstetri cian or nurse) attempted to influence your decision to NOT have a visitor present during placement of neuraxial labor analgesia?

Crosstab

			How often has another non anesthesia member of the labor and delivery team (for example obstetrician or nurse) attempted to influence your decision to NOT have a visitor present during placement of neuraxial labor analgesia?		
			Rarely < 5 %	Occasionally 5 - 40 %	Often > 40 %
On an average, how many neuraxial procedures for labor analgesia do you perform per week?	< 2	Count	65	9	3
		% within On an average, how many neuraxial procedures for labor analgesia do you perform per week?	84.4%	11.7%	3.9%
		Adjusted Residual	1.4	-1.1	-.7
	2- 5	Count	72	9	7
		% within On an average, how many neuraxial procedures for labor analgesia do you perform per week?	81.8%	10.2%	8.0%
		Adjusted Residual	.9	-1.7	1.1
	6-10	Count	49	16	5
		% within On an average, how many neuraxial procedures for labor analgesia do you perform per week?	70.0%	22.9%	7.1%
		Adjusted Residual	-2.0	1.8	.6

Crosstab

			Total
On an average, how many neuraxial procedures for labor analgesia do you perform per week?	< 2	Count	77
		% within On an average, how many neuraxial procedures for labor analgesia do you perform per week?	100.0%
		Adjusted Residual	
	2- 5	Count	88
		% within On an average, how many neuraxial procedures for labor analgesia do you perform per week?	100.0%
		Adjusted Residual	
	6-10	Count	70
		% within On an average, how many neuraxial procedures for labor analgesia do you perform per week?	100.0%
		Adjusted Residual	

Crosstab

		How often has another non anesthesia member of the labor and delivery team (for example obstetrician or nurse) attempted to influence your decision to NOT have a visitor present during placement of neuraxial labor analgesia?		
		Rarely < 5 %	Occasionally 5 - 40 %	Often > 40 %
>10	Count	67	17	3
	% within On an average, how many neuraxial procedures for labor analgesia do you perform per week?	77.0%	19.5%	3.4%
	Adjusted Residual	-.4	1.1	-1.0
Total	Count	253	51	18
	% within On an average, how many neuraxial procedures for labor analgesia do you perform per week?	78.6%	15.8%	5.6%

Crosstab

		Total
>10	Count	87
	% within On an average, how many neuraxial procedures for labor analgesia do you perform per week?	100.0%
	Adjusted Residual	
Total	Count	322
	% within On an average, how many neuraxial procedures for labor analgesia do you perform per week?	100.0%

Chi-Square Tests

	Value	df	Asymp. Sig. (2-sided)
Pearson Chi-Square	8.942[a]	6	.177
Likelihood Ratio	9.012	6	.173
Linear-by-Linear Association	1.115	1	.291
N of Valid Cases	322		

a. 4 cells (33.3%) have expected count less than 5. The minimum expected count is 3.91.

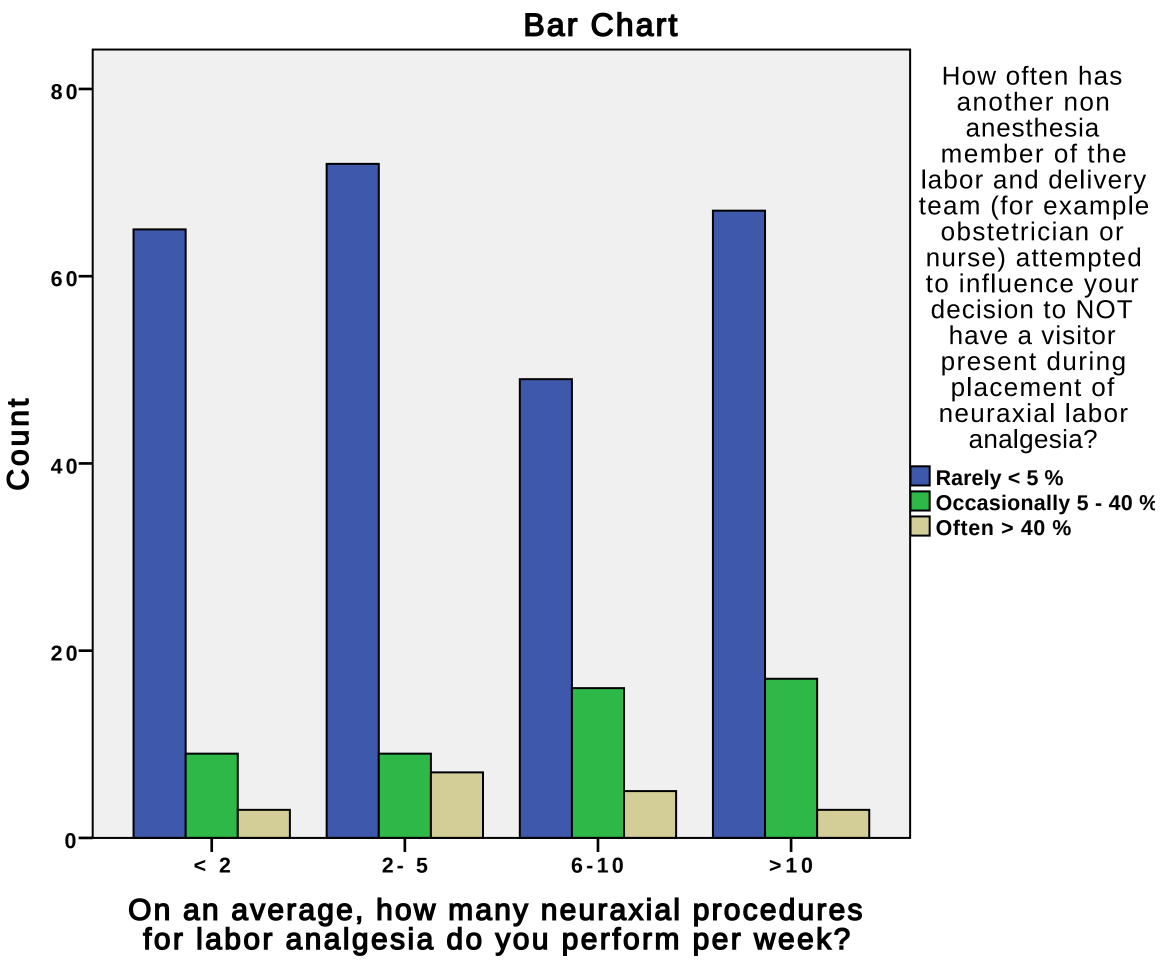

On an average, how many neuraxial procedures for labor analgesia do you perform per week? * What would be your single most important reason for NOT allowing a patient's visitor in the room during placement of neuraxial labor analgesia?

Crosstab

			What would be your single most important reason for NOT allowing a patient's visitor in the room during placement of neuraxial labor ..		
			Increase in anesthesiol ogist's stress	Possible interference by visitor(for e.g. comments made if d	Concern about visitor (for e.g. passing out or nc being ab
On an average, how many neuraxial procedures for labor analgesia do you perform per week?	< 2	Count	13	37	20
		% within On an average, how many neuraxial procedures for labor analgesia do you perform per week?	16.9%	48.1%	26.0%
		Adjusted Residual	3.8	-.1	-1.5
	2- 5	Count	3	46	28
		% within On an average, how many neuraxial procedures for labor analgesia do you perform per week?	3.4%	52.3%	31.8%
		Adjusted Residual	-1.6	.8	-.3
	6-10	Count	2	35	26
		% within On an average, how many neuraxial procedures for labor analgesia do you perform per week?	2.9%	50.0%	37.1%
		Adjusted Residual	-1.6	.3	.9

Crosstab

			What would be your single most important ...	
			Medico-legal concerns	Total
On an average, how many neuraxial procedures for labor analgesia do you perform per week?	< 2	Count	7	77
		% within On an average, how many neuraxial procedures for labor analgesia do you perform per week?	9.1%	100.0%
		Adjusted Residual	-.8	
	2- 5	Count	11	88
		% within On an average, how many neuraxial procedures for labor analgesia do you perform per week?	12.5%	100.0%
		Adjusted Residual	.3	
	6-10	Count	7	70
		% within On an average, how many neuraxial procedures for labor analgesia do you perform per week?	10.0%	100.0%
		Adjusted Residual	-.4	

Crosstab

		What would be your single most important reason for NOT allowing a patient’s visitor in the room during placement of neuraxial labor ..		
		Increase in anesthesiol ogist's stress	Possible interference by visitor(for e.g. comments made if d	Concern about visitor (for e.g. passing out or no being ab
>10	Count	5	38	32
	% within On an average, how many neuraxial procedures for labor analgesia do you perform per week?	5.7%	43.7%	36.8%
	Adjusted Residual	-.6	-1.0	.9
Total	Count	23	156	106
	% within On an average, how many neuraxial procedures for labor analgesia do you perform per week?	7.1%	48.4%	32.9%

Crosstab

		What would be your single most important ...	
		Medico-legal concerns	Total
>10	Count	12	87
	% within On an average, how many neuraxial procedures for labor analgesia do you perform per week?	13.8%	100.0%
	Adjusted Residual	.8	
Total	Count	37	322
	% within On an average, how many neuraxial procedures for labor analgesia do you perform per week?	11.5%	100.0%

Chi-Square Tests

	Value	df	Asymp. Sig. (2-sided)
Pearson Chi-Square	17.629[a]	9	.040
Likelihood Ratio	15.811	9	.071
Linear-by-Linear Association	5.536	1	.019
N of Valid Cases	322		

a. 0 cells (0.0%) have expected count less than 5. The minimum expected count is 5.00.

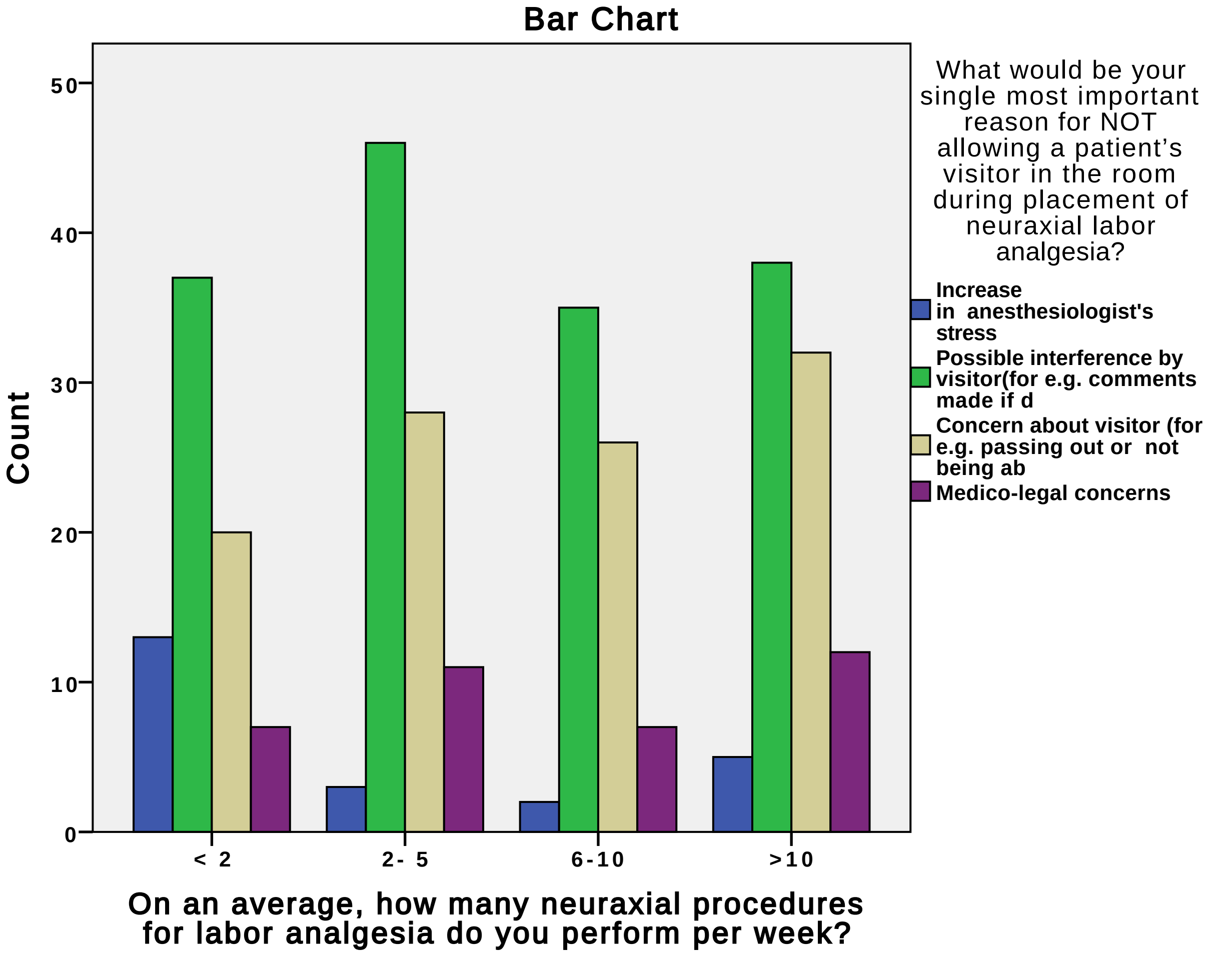

How many years have you been practicing as an attending anesthesiologist? * What is your preferred technique for neuraxial labor analgesia in an otherwise healthy parturient?

Crosstab

			What is your preferred technique for neuraxial labor analgesia in an otherwise healthy parturient?		
			Epidural analgesia	Combined spinal epidural analgesia	Total
How many years have you been practicing as an attending anesthesiologist?	< 2 years	Count	107	30	137
		% within How many years have you been practicing as an attending anesthesiologist?	78.1%	21.9%	100.0%
		Adjusted Residual	.0	.0	
	2-5 years	Count	192	49	241
		% within How many years have you been practicing as an attending anesthesiologist?	79.7%	20.3%	100.0%
		Adjusted Residual	.7	-.7	
	6-15 years	Count	264	74	338
		% within How many years have you been practicing as an attending anesthesiologist?	78.1%	21.9%	100.0%
		Adjusted Residual	.0	.0	
	>15 years	Count	452	133	585
		% within How many years have you been practicing as an attending anesthesiologist?	77.3%	22.7%	100.0%
		Adjusted Residual	-.6	.6	
Total		Count	1015	286	1301
		% within How many years have you been practicing as an attending anesthesiologist?	78.0%	22.0%	100.0%

Chi-Square Tests

	Value	df	Asymp. Sig. (2-sided)
Pearson Chi-Square	.578[a]	3	.901
Likelihood Ratio	.584	3	.900
Linear-by-Linear Association	.315	1	.574
N of Valid Cases	1301		

a. 0 cells (0.0%) have expected count less than 5. The minimum expected count is 30.12.

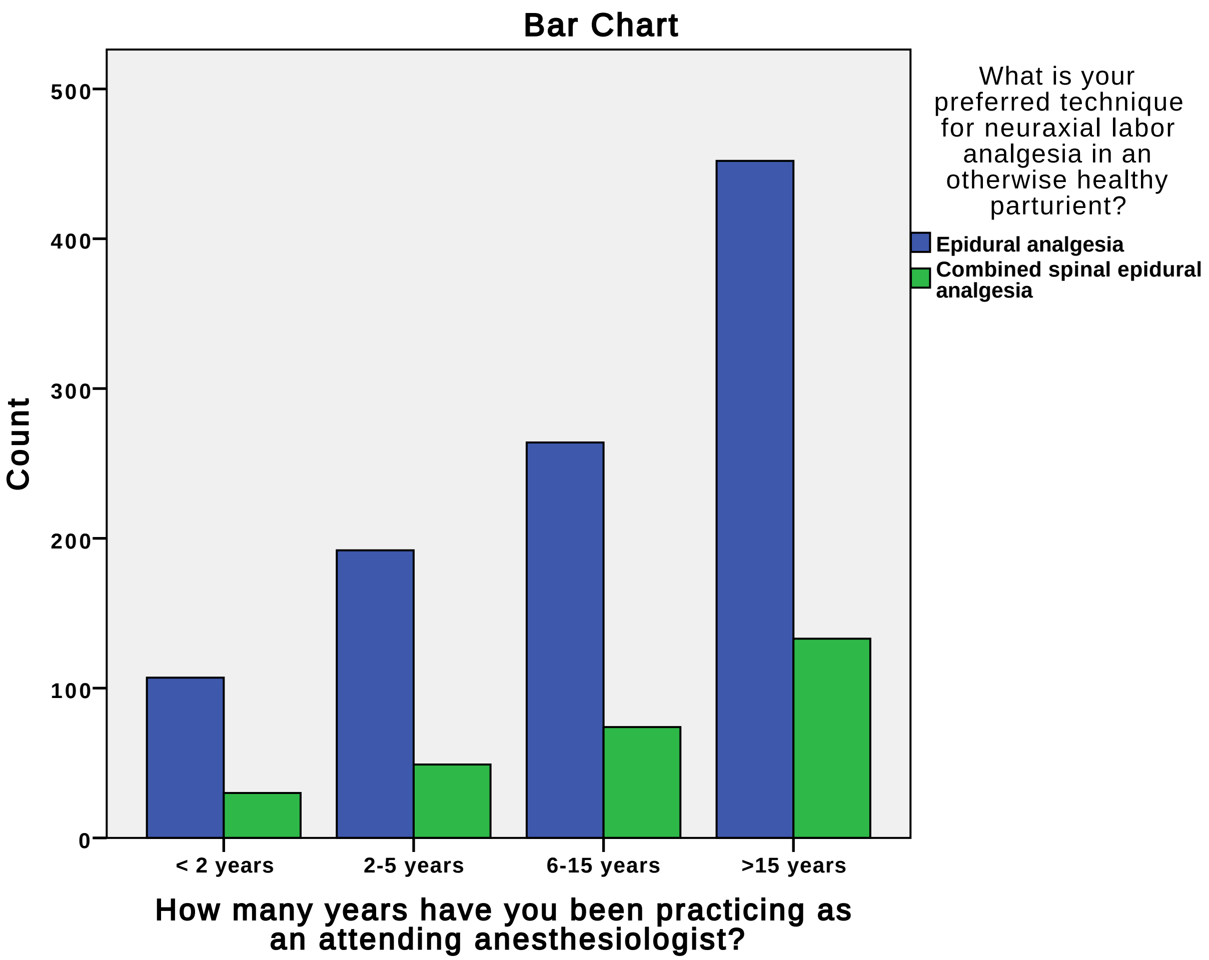

How many years have you been practicing as an attending anesthesiologist? * Does your practice or hospital have a written policy regarding allowing a patient's visitor in the room during placement of neuraxial labor analgesia?

Crosstab

			Does your practice or hospital have a written policy regarding allowing a patient's visitor in the room during placement of neuraxial labor ..		
			Yes, a written policy exists - Allows visitors	Yes, a written policy exists - Does not allow visitors	No written policy exists
How many years have you been practicing as an attending anesthesiologist?	< 2 years	Count	29	8	44
		% within How many years have you been practicing as an attending anesthesiologist?	21.2%	5.8%	32.1%
		Adjusted Residual	-.4	-1.0	-1.9
	2-5 years	Count	41	17	84
		% within How many years have you been practicing as an attending anesthesiologist?	17.0%	7.1%	34.9%
		Adjusted Residual	-2.3	-.6	-1.6
	6-15 years	Count	68	28	140
		% within How many years have you been practicing as an attending anesthesiologist?	20.1%	8.3%	41.4%
		Adjusted Residual	-1.2	.2	.9
	>15 years	Count	155	52	245
		% within How many years have you been practicing as an attending anesthesiologist?	26.5%	8.9%	41.9%
		Adjusted Residual	3.1	1.0	1.6
Total		Count	293	105	513
		% within How many years have you been practicing as an attending anesthesiologist?	22.5%	8.1%	39.4%

Crosstab

			Does your practice or hospital have a ...	
			I do not know	Total
How many years have you been practicing as an attending anesthesiologist?	< 2 years	Count	56	137
		% within How many years have you been practicing as an attending anesthesiologist?	40.9%	100.0%
		Adjusted Residual	2.9	
	2-5 years	Count	99	241
		% within How many years have you been practicing as an attending anesthesiologist?	41.1%	100.0%
		Adjusted Residual	4.2	
	6-15 years	Count	102	338
		% within How many years have you been practicing as an attending anesthesiologist?	30.2%	100.0%
		Adjusted Residual	.1	
	>15 years	Count	133	585
		% within How many years have you been practicing as an attending anesthesiologist?	22.7%	100.0%
		Adjusted Residual	-5.2	
Total		Count	390	1301
		% within How many years have you been practicing as an attending anesthesiologist?	30.0%	100.0%

Chi-Square Tests

	Value	df	Asymp. Sig. (2-sided)
Pearson Chi-Square	39.932[a]	9	.000
Likelihood Ratio	39.637	9	.000
Linear-by-Linear Association	22.079	1	.000
N of Valid Cases	1301		

a. 0 cells (0.0%) have expected count less than 5. The minimum expected count is 11.06.

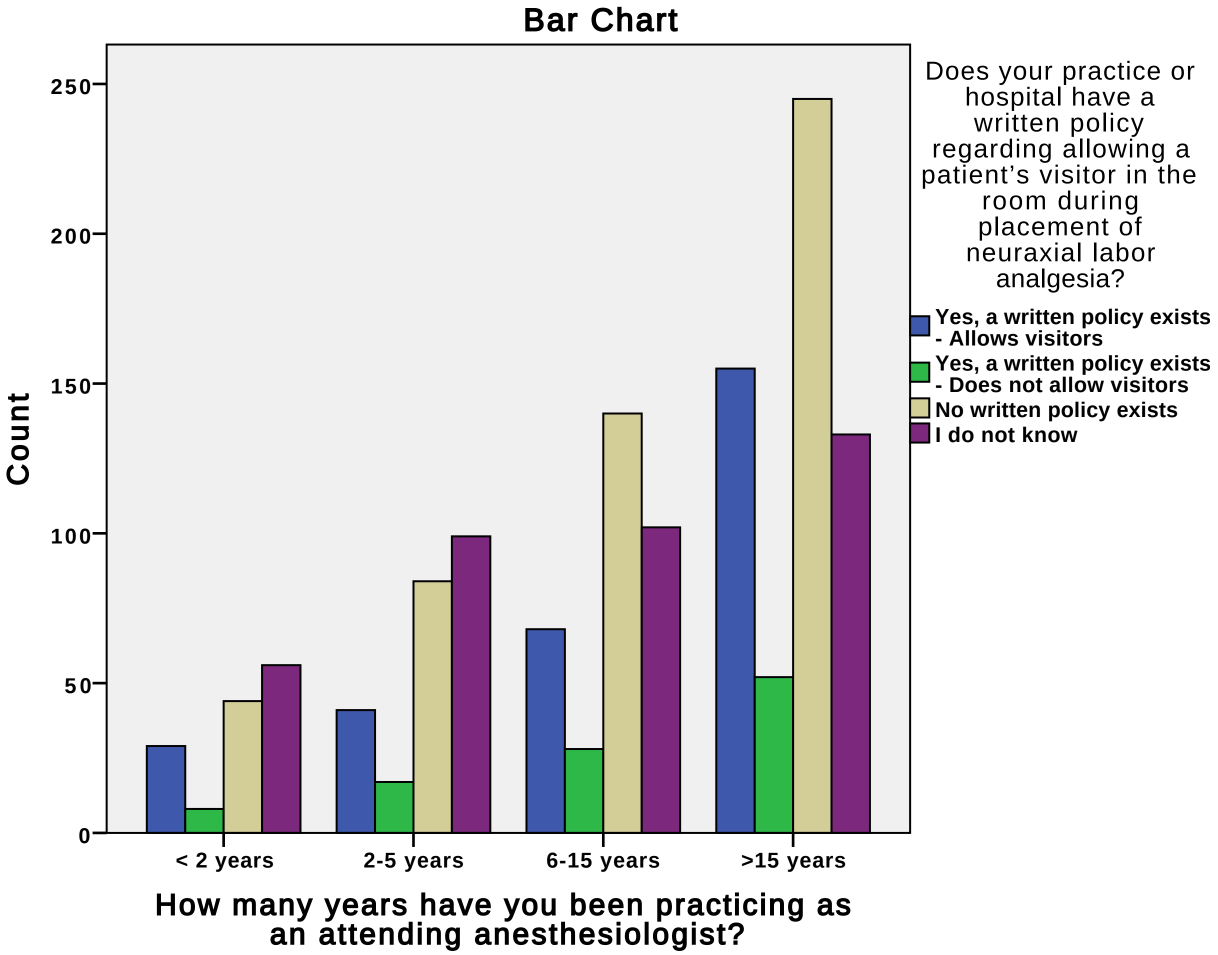

How many years have you been practicing as an attending anesthesiologist? * If no policy existed, would you be open to allowing a patient's visitor in the room during placement of neuraxial labor analgesia?

Crosstab

			If no policy existed, would you be open to allowing a patient's visitor in the room during placement of neuraxial labor analgesia?		
			Yes	No	Total
How many years have you been practicing as an attending anesthesiologist?	< 2 years	Count	99	38	137
		% within How many years have you been practicing as an attending anesthesiologist?	72.3%	27.7%	100.0%
		Adjusted Residual	-.8	.8	
	2-5 years	Count	193	48	241
		% within How many years have you been practicing as an attending anesthesiologist?	80.1%	19.9%	100.0%
		Adjusted Residual	2.0	-2.0	
	6-15 years	Count	259	79	338
		% within How many years have you been practicing as an attending anesthesiologist?	76.6%	23.4%	100.0%
		Adjusted Residual	.8	-.8	
	>15 years	Count	426	159	585
		% within How many years have you been practicing as an attending anesthesiologist?	72.8%	27.2%	100.0%
		Adjusted Residual	-1.7	1.7	
Total		Count	977	324	1301
		% within How many years have you been practicing as an attending anesthesiologist?	75.1%	24.9%	100.0%

Chi-Square Tests

	Value	df	Asymp. Sig. (2-sided)
Pearson Chi-Square	5.836[a]	3	.120
Likelihood Ratio	5.959	3	.114
Linear-by-Linear Association	1.203	1	.273
N of Valid Cases	1301		

a. 0 cells (0.0%) have expected count less than 5. The minimum expected count is 34.12.

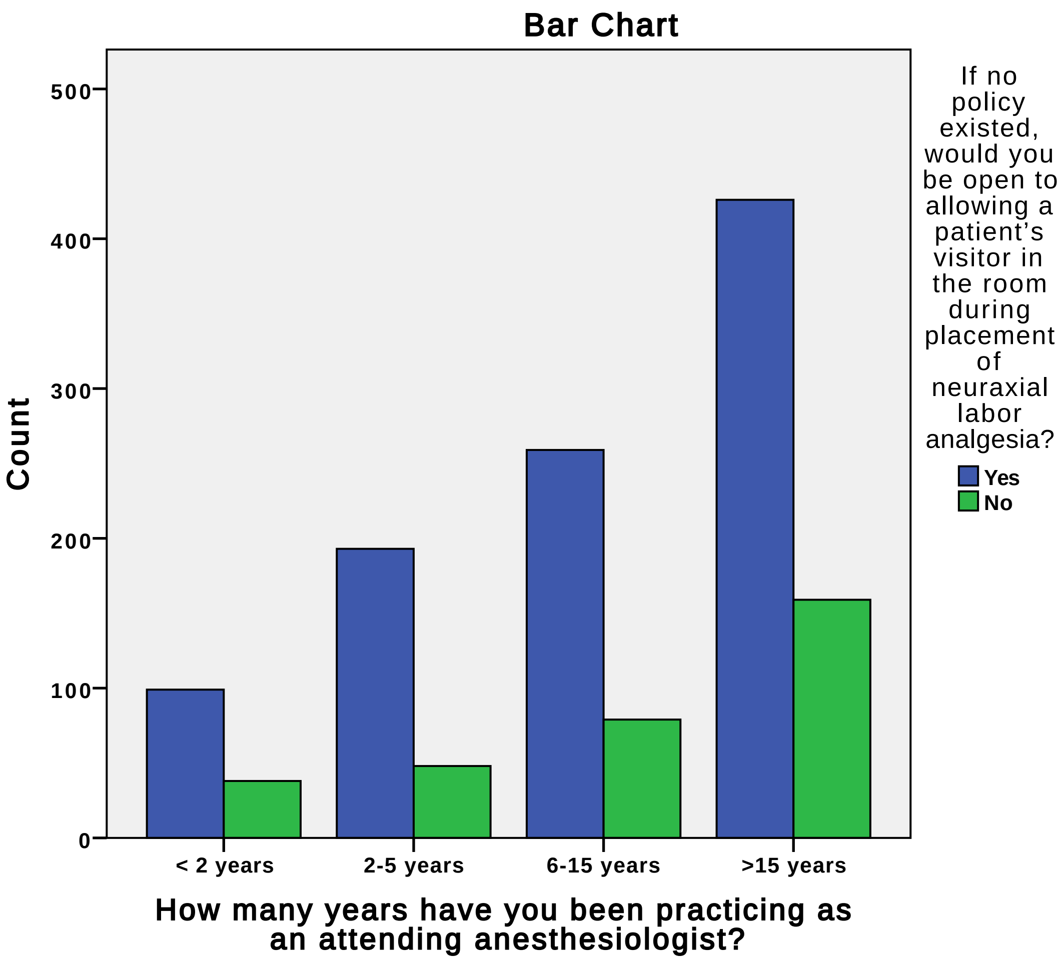

How many years have you been practicing as an attending anesthesiologist? * If you allowed a visitor in the room during placement of neuraxial labor analgesia, you would want the

Crosstab

			If you allowed a visitor in the room during placement of neuraxial labor analgesia, you would want the		
			Visitor always standing	Visitor always sitting	Does not matter
How many years have you been practicing as an attending anesthesiologist?	< 2 years	Count	4	78	19
		% within How many years have you been practicing as an attending anesthesiologist?	4.0%	77.2%	18.8%
		Adjusted Residual	2.2	1.2	-1.9
	2-5 years	Count	3	146	42
		% within How many years have you been practicing as an attending anesthesiologist?	1.6%	76.4%	22.0%
		Adjusted Residual	.2	1.5	-1.6
	6-15 years	Count	1	191	66
		% within How many years have you been practicing as an attending anesthesiologist?	0.4%	74.0%	25.6%
		Adjusted Residual	-1.7	.8	-.4
	>15 years	Count	6	286	131
		% within How many years have you been practicing as an attending anesthesiologist?	1.4%	67.6%	31.0%
		Adjusted Residual	.0	-2.7	2.8
Total		Count	14	701	258
		% within How many years have you been practicing as an attending anesthesiologist?	1.4%	72.0%	26.5%

Crosstab

			Total
How many years have you been practicing as an attending anesthesiologist?	< 2 years	Count	101
		% within How many years have you been practicing as an attending anesthesiologist?	100.0%
		Adjusted Residual	
	2-5 years	Count	191
		% within How many years have you been practicing as an attending anesthesiologist?	100.0%
		Adjusted Residual	
	6-15 years	Count	258
		% within How many years have you been practicing as an attending anesthesiologist?	100.0%
		Adjusted Residual	
	>15 years	Count	423
		% within How many years have you been practicing as an attending anesthesiologist?	100.0%
		Adjusted Residual	
Total		Count	973
		% within How many years have you been practicing as an attending anesthesiologist?	100.0%

Chi-Square Tests

	Value	df	Asymp. Sig. (2-sided)
Pearson Chi-Square	15.638[a]	6	.016
Likelihood Ratio	15.191	6	.019
Linear-by-Linear Association	10.653	1	.001
N of Valid Cases	973		

a. 3 cells (25.0%) have expected count less than 5. The minimum expected count is 1.45.

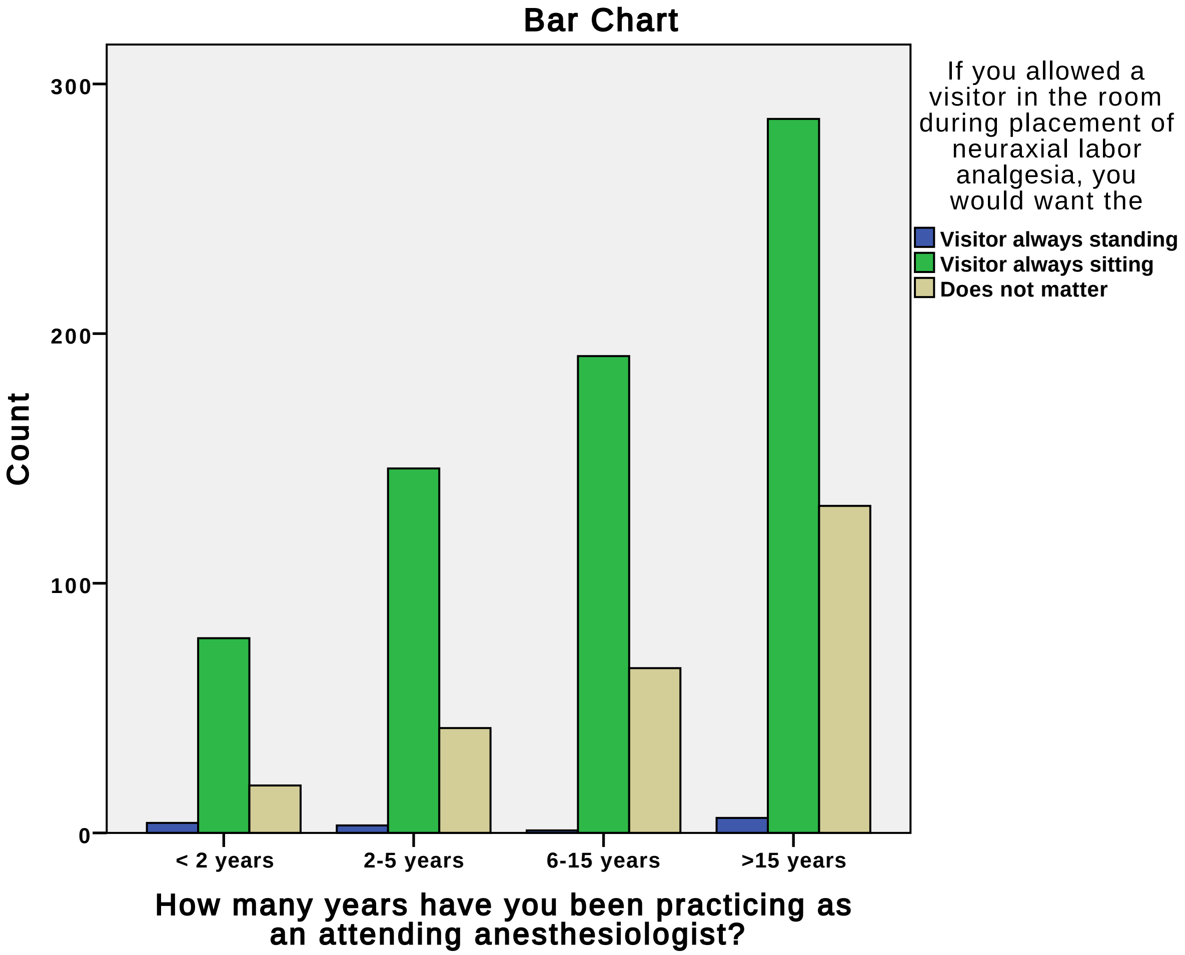

How many years have you been practicing as an attending anesthesiologist? * If you allowed a visitor in the room during placement of neuraxial labor analgesia, you would want the visitor to be positioned such that,

Crosstab

			If you allowed a visitor in the room during placement of neuraxial labor analgesia, you would want the visitor to be positioned such that,		
			Visitor has no view of procedure (patient is in between anes	Visitor has partial view of workspace (but cannot see patien	Does not matter
How many years have you been practicing as an attending anesthesiologist?	< 2 years	Count	66	21	14
		% within How many years have you been practicing as an attending anesthesiologist?	65.3%	20.8%	13.9%
		Adjusted Residual	.9	.5	-1.6
	2-5 years	Count	132	33	26
		% within How many years have you been practicing as an attending anesthesiologist?	69.1%	17.3%	13.6%
		Adjusted Residual	2.5	-.7	-2.4
	6-15 years	Count	162	48	48
		% within How many years have you been practicing as an attending anesthesiologist?	62.8%	18.6%	18.6%
		Adjusted Residual	.7	-.2	-.6
	>15 years	Count	234	83	106
		% within How many years have you been practicing as an attending anesthesiologist?	55.3%	19.6%	25.1%
		Adjusted Residual	-3.2	.4	3.5
Total		Count	594	185	194
		% within How many years have you been practicing as an attending anesthesiologist?	61.0%	19.0%	19.9%

Crosstab

			Total
How many years have you been practicing as an attending anesthesiologist?	< 2 years	Count	101
		% within How many years have you been practicing as an attending anesthesiologist?	100.0%
		Adjusted Residual	
	2-5 years	Count	191
		% within How many years have you been practicing as an attending anesthesiologist?	100.0%
		Adjusted Residual	
	6-15 years	Count	258
		% within How many years have you been practicing as an attending anesthesiologist?	100.0%
		Adjusted Residual	
	>15 years	Count	423
		% within How many years have you been practicing as an attending anesthesiologist?	100.0%
		Adjusted Residual	
Total		Count	973
		% within How many years have you been practicing as an attending anesthesiologist?	100.0%

Chi-Square Tests

	Value	df	Asymp. Sig. (2-sided)
Pearson Chi-Square	16.815[a]	6	.010
Likelihood Ratio	17.094	6	.009
Linear-by-Linear Association	13.638	1	.000
N of Valid Cases	973		

a. 0 cells (0.0%) have expected count less than 5. The minimum expected count is 19.20.

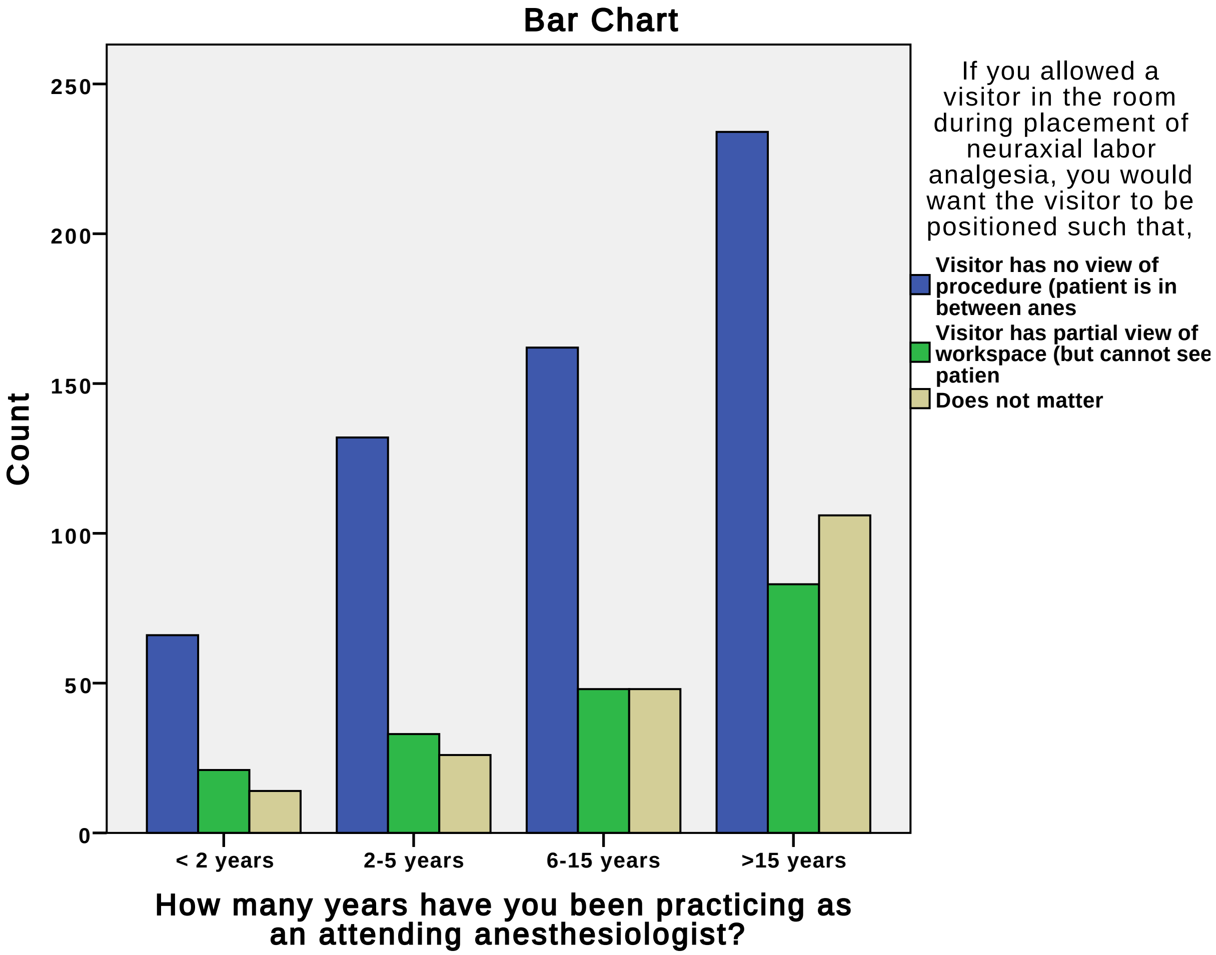

How many years have you been practicing as an attending anest hesiologist? * What would be your single most important reason f or allowing a patient’s visitor in the room during placement of ne uraxial labor analgesia?

Crosstab

			What would be your single most important reason for allowing a patient's visitor in the room during placement of neuraxial labor ...		
			It would likely reduce patient's anxiety	It would likely reduce visitor's anxiety (for e. g. if visito	Visitor's assistance needed (for e. g. if visitor were a doul
How many years have you been practicing as an attending anesthesiologist?	< 2 years	Count	72	8	3
		% within How many years have you been practicing as an attending anesthesiologist?	71.3%	7.9%	3.0%
		Adjusted Residual	1.5	1.4	-.5
	2-5 years	Count	133	11	8
		% within How many years have you been practicing as an attending anesthesiologist?	69.6%	5.8%	4.2%
		Adjusted Residual	1.6	.5	.3
	6-15 years	Count	154	9	8
		% within How many years have you been practicing as an attending anesthesiologist?	59.7%	3.5%	3.1%
		Adjusted Residual	-1.9	-1.3	-.7
	>15 years	Count	270	21	18
		% within How many years have you been practicing as an attending anesthesiologist?	63.8%	5.0%	4.3%
		Adjusted Residual	-.5	-.1	.6
Total		Count	629	49	37
		% within How many years have you been practicing as an attending anesthesiologist?	64.6%	5.0%	3.8%

Crosstab

			What would be your single most important ...	Total
			To fulfill patient's request	
How many years have you been practicing as an attending anesthesiologist?	< 2 years	Count	18	101
		% within How many years have you been practicing as an attending anesthesiologist?	17.8%	100.0%
		Adjusted Residual	-2.1	
	2-5 years	Count	39	191
		% within How many years have you been practicing as an attending anesthesiologist?	20.4%	100.0%
		Adjusted Residual	-2.1	
	6-15 years	Count	87	258
		% within How many years have you been practicing as an attending anesthesiologist?	33.7%	100.0%
		Adjusted Residual	3.1	
	>15 years	Count	114	423
		% within How many years have you been practicing as an attending anesthesiologist?	27.0%	100.0%
		Adjusted Residual	.3	
Total		Count	258	973
		% within How many years have you been practicing as an attending anesthesiologist?	26.5%	100.0%

Chi-Square Tests

	Value	df	Asymp. Sig. (2-sided)
Pearson Chi-Square	17.006[a]	9	.049
Likelihood Ratio	17.116	9	.047
Linear-by-Linear Association	4.712	1	.030
N of Valid Cases	973		

a. 1 cells (6.3%) have expected count less than 5. The minimum expected count is 3.84.

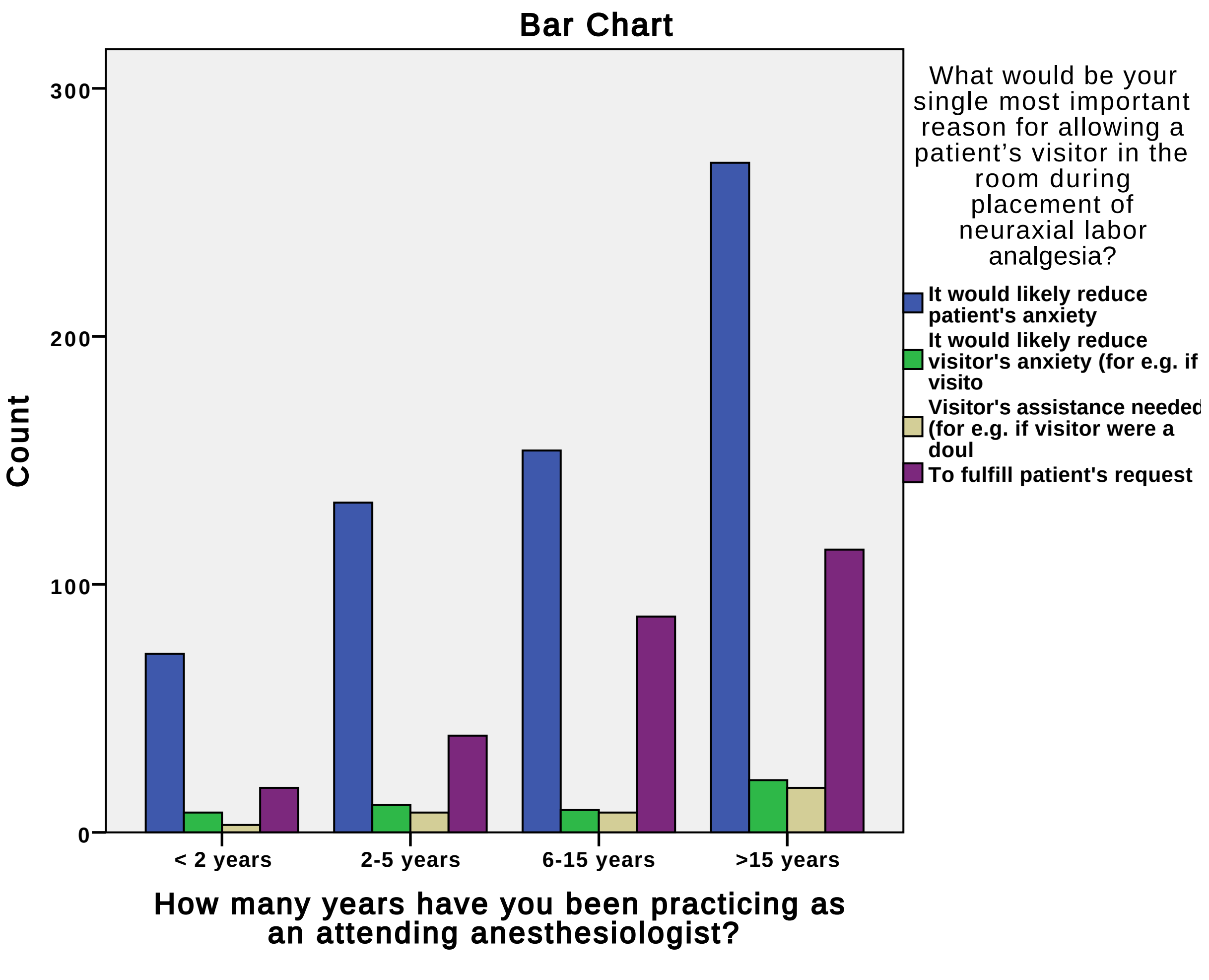

How many years have you been practicing as an attending anesthesiologist? * How often has another non anesthesia member of the labor and delivery team (for example obstetrician or nurse) attempted to influence your decision to have a visitor present during placement of neuraxial labor analgesia?

Crosstab

			How often has another non anesthesia member of the labor and delivery team (for example obstetrician or nurse) attempted to influence your decision to have a visitor present during placement of neuraxial labor analgesia?		
			Rarely (< 5 %)	Occasionally (5 % - 40 %)	Often > 40 %
How many years have you been practicing as an attending anesthesiologist?	< 2 years	Count	73	20	8
		% within How many years have you been practicing as an attending anesthesiologist?	72.3%	19.8%	7.9%
		Adjusted Residual	-.3	.5	-.1
	2-5 years	Count	142	35	14
		% within How many years have you been practicing as an attending anesthesiologist?	74.3%	18.3%	7.3%
		Adjusted Residual	.2	.1	-.5
	6-15 years	Count	183	46	29
		% within How many years have you been practicing as an attending anesthesiologist?	70.9%	17.8%	11.2%
		Adjusted Residual	-1.2	-.1	2.1
	>15 years	Count	319	75	29
		% within How many years have you been practicing as an attending anesthesiologist?	75.4%	17.7%	6.9%
		Adjusted Residual	1.1	-.3	-1.4
Total		Count	717	176	80
		% within How many years have you been practicing as an attending anesthesiologist?	73.7%	18.1%	8.2%

Crosstab

			Total
How many years have you been practicing as an attending anesthesiologist?	< 2 years	Count	101
		% within How many years have you been practicing as an attending anesthesiologist?	100.0%
		Adjusted Residual	
	2-5 years	Count	191
		% within How many years have you been practicing as an attending anesthesiologist?	100.0%
		Adjusted Residual	
	6-15 years	Count	258
		% within How many years have you been practicing as an attending anesthesiologist?	100.0%
		Adjusted Residual	
	>15 years	Count	423
		% within How many years have you been practicing as an attending anesthesiologist?	100.0%
		Adjusted Residual	
Total		Count	973
		% within How many years have you been practicing as an attending anesthesiologist?	100.0%

Chi-Square Tests

	Value	df	Asymp. Sig. (2-sided)
Pearson Chi-Square	4.700[a]	6	.583
Likelihood Ratio	4.466	6	.614
Linear-by-Linear Association	.384	1	.535
N of Valid Cases	973		

a. 0 cells (0.0%) have expected count less than 5. The minimum expected count is 8.30.

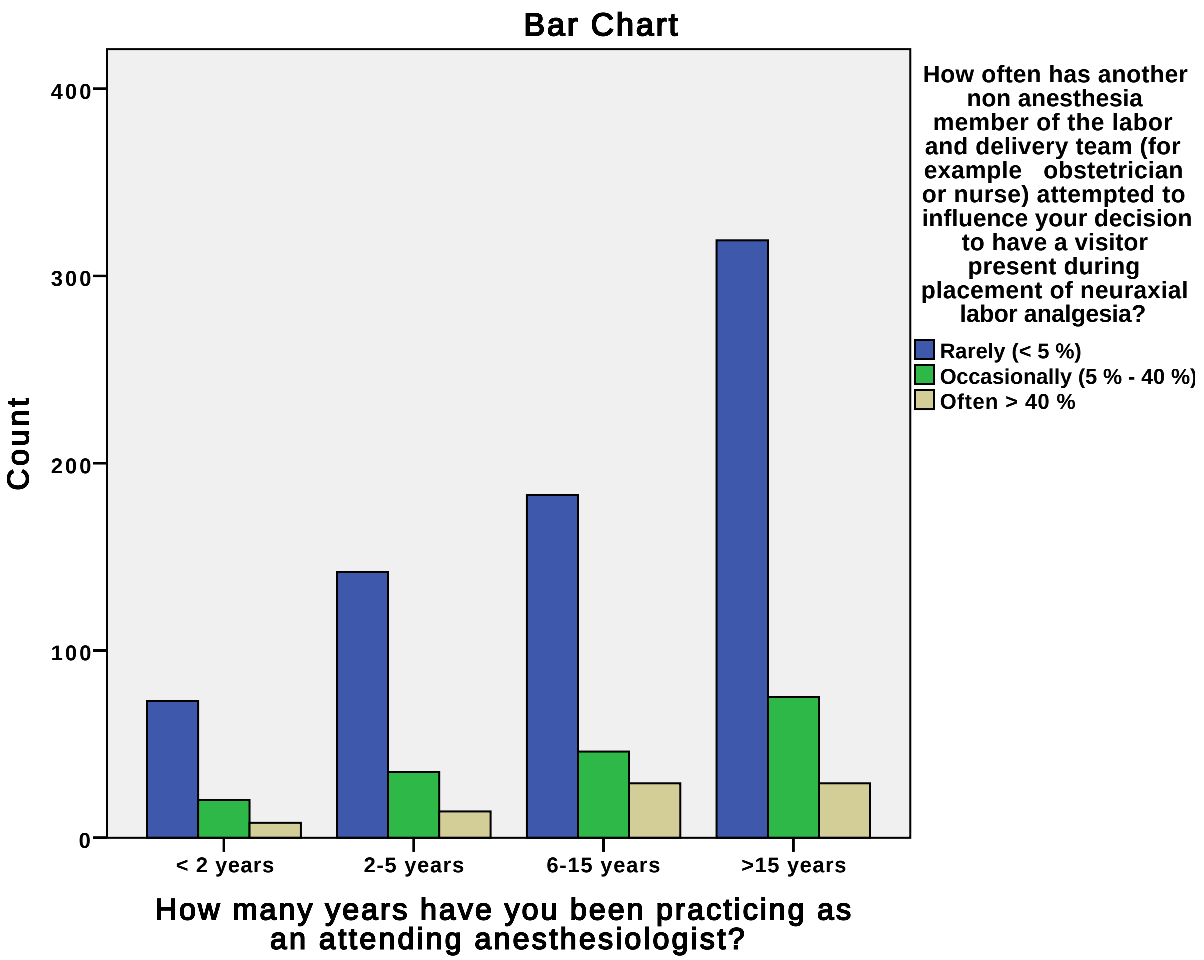

How many years have you been practicing as an attending anesthesiologist? * How often has another non anesthesia member of the labor and delivery team (for example obstetrician or nurse) attempted to influence your decision to NOT have a visitor present during placement of neuraxial labor analgesia?

Crosstab

			How often has another non anesthesia member of the labor and delivery team (for example obstetrician or nurse) attempted to influence your decision to NOT have a visitor present during placement of neuraxial labor analgesia?		
			Rarely < 5 %	Occasionally 5 - 40 %	Often > 40 %
How many years have you been practicing as an attending anesthesiologist?	< 2 years	Count	27	5	4
		% within How many years have you been practicing as an attending anesthesiologist?	75.0%	13.9%	11.1%
		Adjusted Residual	-.6	-.3	1.5
	2-5 years	Count	36	6	5
		% within How many years have you been practicing as an attending anesthesiologist?	76.6%	12.8%	10.6%
		Adjusted Residual	-.4	-.6	1.6
	6-15 years	Count	67	9	3
		% within How many years have you been practicing as an attending anesthesiologist?	84.8%	11.4%	3.8%
		Adjusted Residual	1.6	-1.2	-.8
	>15 years	Count	123	31	6
		% within How many years have you been practicing as an attending anesthesiologist?	76.9%	19.4%	3.8%
		Adjusted Residual	-.7	1.7	-1.4
Total		Count	253	51	18
		% within How many years have you been practicing as an attending anesthesiologist?	78.6%	15.8%	5.6%

Crosstab

			Total
How many years have you been practicing as an attending anesthesiologist?	< 2 years	Count	36
		% within How many years have you been practicing as an attending anesthesiologist?	100.0%
		Adjusted Residual	
	2-5 years	Count	47
		% within How many years have you been practicing as an attending anesthesiologist?	100.0%
		Adjusted Residual	
	6-15 years	Count	79
		% within How many years have you been practicing as an attending anesthesiologist?	100.0%
		Adjusted Residual	
	>15 years	Count	160
		% within How many years have you been practicing as an attending anesthesiologist?	100.0%
		Adjusted Residual	
Total		Count	322
		% within How many years have you been practicing as an attending anesthesiologist?	100.0%

Chi-Square Tests

	Value	df	Asymp. Sig. (2-sided)
Pearson Chi-Square	8.677[a]	6	.193
Likelihood Ratio	8.006	6	.238
Linear-by-Linear Association	.948	1	.330
N of Valid Cases	322		

a. 3 cells (25.0%) have expected count less than 5. The minimum expected count is 2.01.

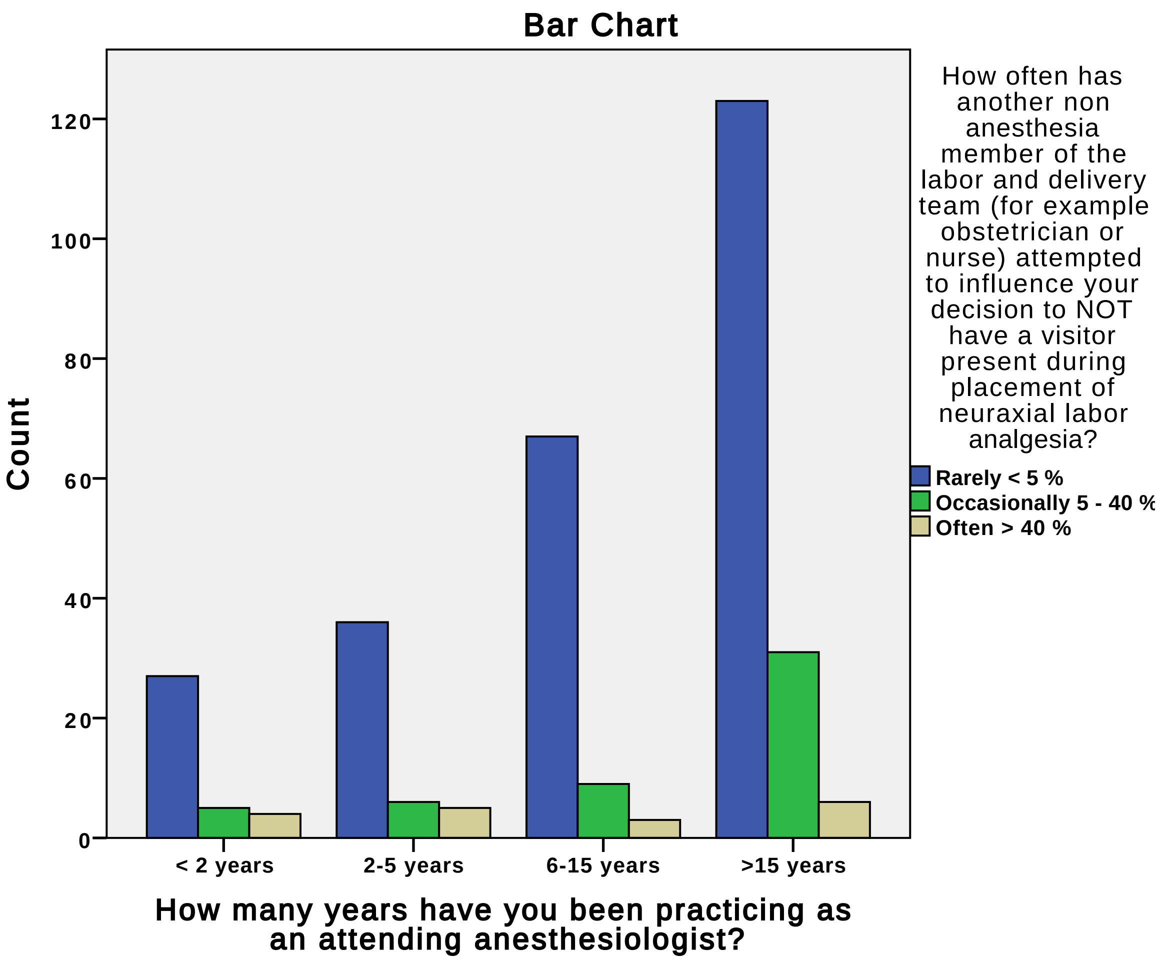

How many years have you been practicing as an attending anesthesiologist? * What would be your single most important reason for NOT allowing a patient's visitor in the room during placement of neuraxial labor analgesia?

Crosstab

			What would be your single most important reason for NOT allowing a patient's visitor in the room during placement of neuraxial labor ..		
			Increase in anesthesiol ogist's stress	Possible interference by visitor(for e.g. comments made if d	Concern about visitor (for e.g. passing out or no being ab
How many years have you been practicing as an attending anesthesiologist?	< 2 years	Count	5	17	10
		% within How many years have you been practicing as an attending anesthesiologist?	13.9%	47.2%	27.8%
		Adjusted Residual	1.7	-.2	-.7
	2-5 years	Count	3	33	10
		% within How many years have you been practicing as an attending anesthesiologist?	6.4%	70.2%	21.3%
		Adjusted Residual	-.2	3.2	-1.8
	6-15 years	Count	1	36	33
		% within How many years have you been practicing as an attending anesthesiologist?	1.3%	45.6%	41.8%
		Adjusted Residual	-2.3	-.6	1.9
	>15 years	Count	14	70	53
		% within How many years have you been practicing as an attending anesthesiologist?	8.8%	43.8%	33.1%
		Adjusted Residual	1.1	-1.7	.1
Total		Count	23	156	106
		% within How many years have you been practicing as an attending anesthesiologist?	7.1%	48.4%	32.9%

Crosstab

			What would be your single most important ... Medico-legal concerns	Total
How many years have you been practicing as an attending anesthesiologist?	< 2 years	Count	4	36
		% within How many years have you been practicing as an attending anesthesiologist?	11.1%	100.0%
		Adjusted Residual	-.1	
	2-5 years	Count	1	47
		% within How many years have you been practicing as an attending anesthesiologist?	2.1%	100.0%
		Adjusted Residual	-2.2	
	6-15 years	Count	9	79
		% within How many years have you been practicing as an attending anesthesiologist?	11.4%	100.0%
		Adjusted Residual	.0	
	>15 years	Count	23	160
		% within How many years have you been practicing as an attending anesthesiologist?	14.4%	100.0%
		Adjusted Residual	1.6	
Total		Count	37	322
		% within How many years have you been practicing as an attending anesthesiologist?	11.5%	100.0%

Chi-Square Tests

	Value	df	Asymp. Sig. (2-sided)
Pearson Chi-Square	21.058[a]	9	.012
Likelihood Ratio	23.889	9	.004
Linear-by-Linear Association	4.171	1	.041
N of Valid Cases	322		

a. 3 cells (18.8%) have expected count less than 5. The minimum expected count is 2.57.

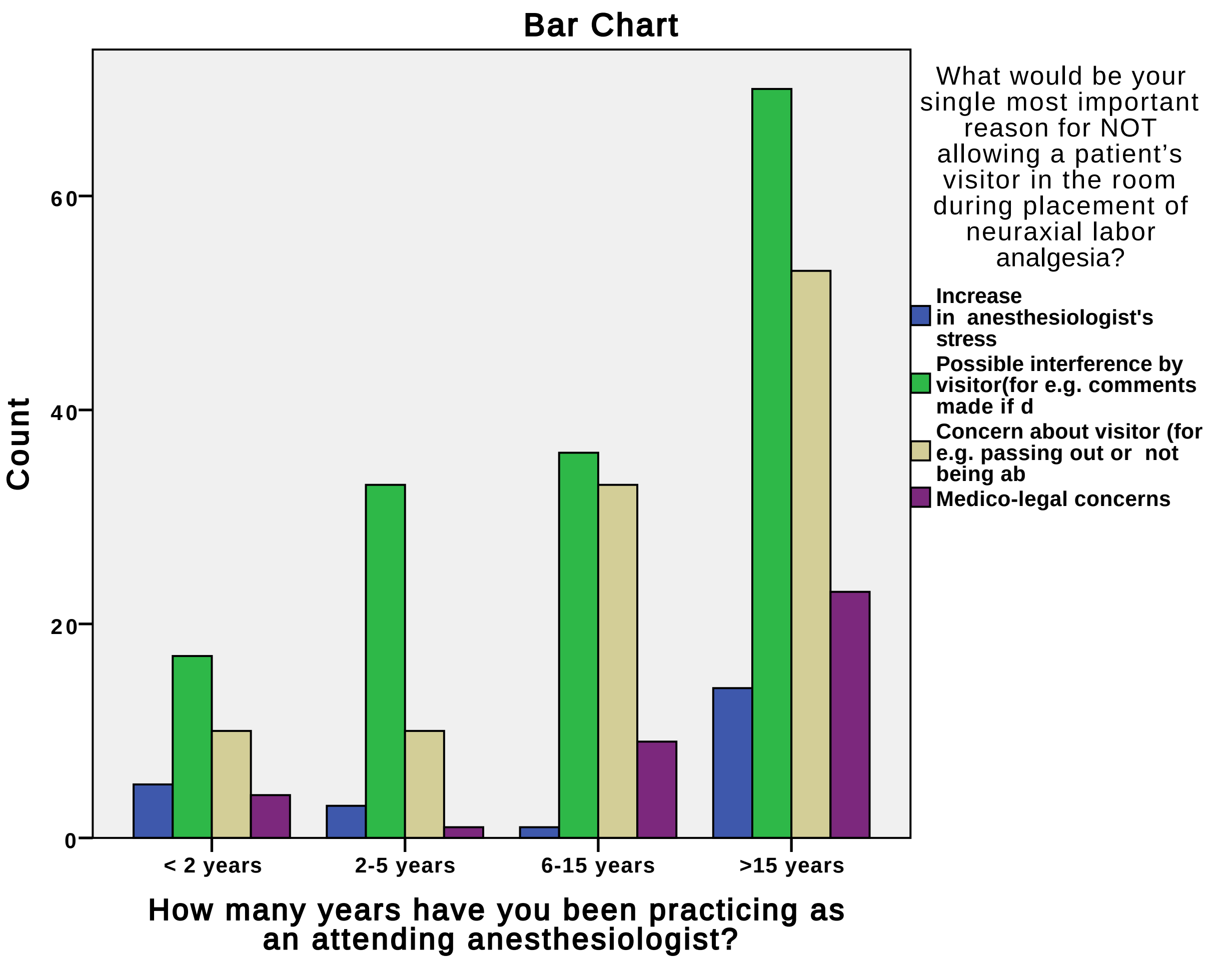

What is your qualification ? * What is your preferred technique for neuraxial labor analgesia in an otherwise healthy parturient?

Crosstab

			What is your preferred technique for neuraxial labor analgesia in an otherwise healthy parturient?		
			Epidural analgesia	Combined spinal epidural analgesia	Total
What is your qualification ?	Board eligible	Count	75	15	90
		% within What is your qualification ?	83.3%	16.7%	100.0%
		Adjusted Residual	1.3	-1.3	
	Board certified	Count	823	199	1022
		% within What is your qualification ?	80.5%	19.5%	100.0%
		Adjusted Residual	4.2	-4.2	
	Board eligible and obstetric anesthesia fellowship trained	Count	10	12	22
		% within What is your qualification ?	45.5%	54.5%	100.0%
		Adjusted Residual	-3.7	3.7	
	Board certified and obstetric anesthesia fellowship trained	Count	107	60	167
		% within What is your qualification ?	64.1%	35.9%	100.0%
		Adjusted Residual	-4.7	4.7	
Total		Count	1015	286	1301
		% within What is your qualification ?	78.0%	22.0%	100.0%

Chi-Square Tests

	Value	df	Asymp. Sig. (2-sided)
Pearson Chi-Square	37.779[a]	3	.000
Likelihood Ratio	33.266	3	.000
Linear-by-Linear Association	27.502	1	.000
N of Valid Cases	1301		

a. 1 cells (12.5%) have expected count less than 5. The minimum expected count is 4.84.

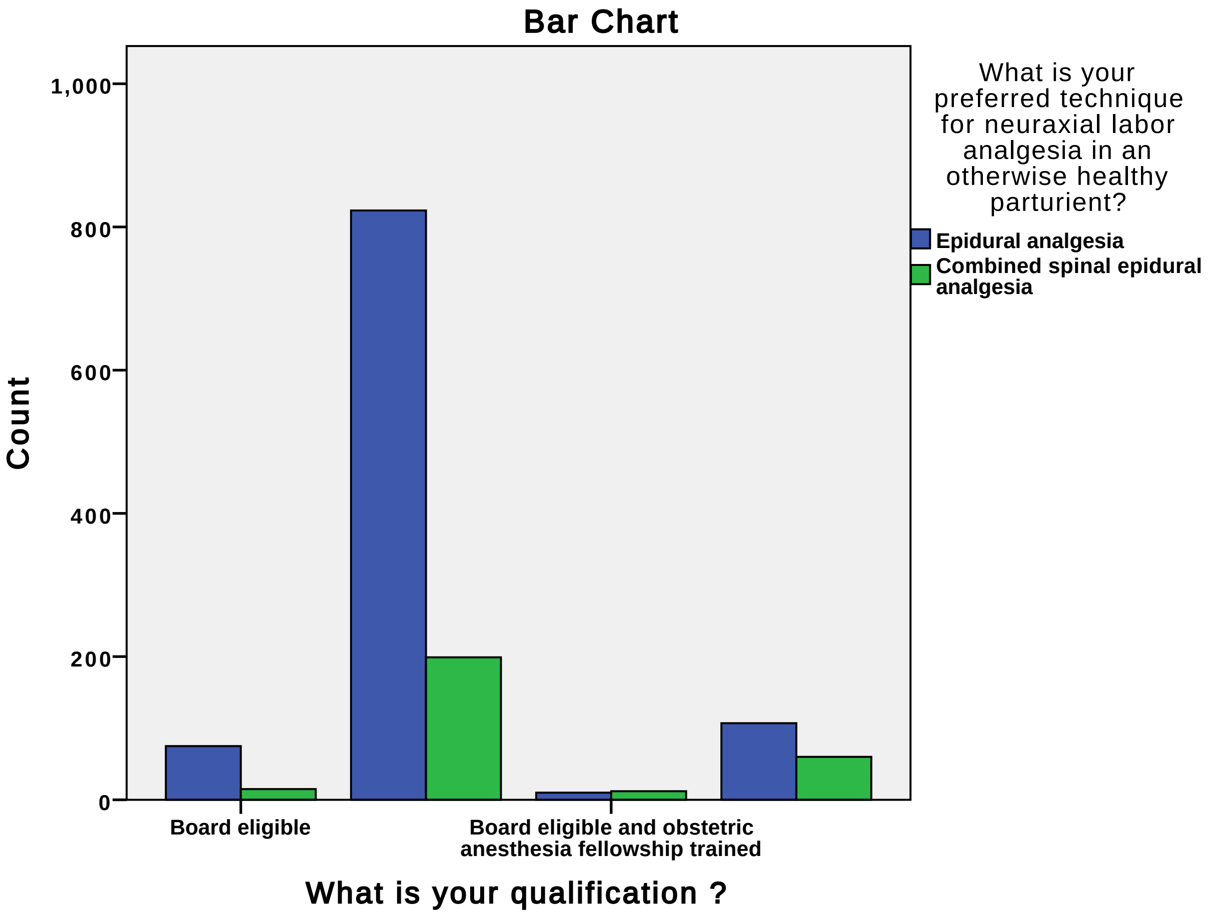

What is your qualification ? * Does your practice or hospital have a written policy regarding allowing a patient's visitor in the room during placement of neuraxial labor analgesia?

Crosstab

			Does your practice or hospital have a written policy regarding allowing a patient’s visitor in ..	
			Yes, a written policy exists - Allows visitors	Yes, a written policy exists - Does not allow visitors
What is your qualification ?	Board eligible	Count	14	5
		% within What is your qualification ?	15.6%	5.6%
		Adjusted Residual	-1.6	-.9
	Board certified	Count	206	77
		% within What is your qualification ?	20.2%	7.5%
		Adjusted Residual	-3.9	-1.4
	Board eligible and obstetric anesthesia fellowship trained	Count	3	2
		% within What is your qualification ?	13.6%	9.1%
		Adjusted Residual	-1.0	.2
	Board certified and obstetric anesthesia fellowship trained	Count	70	21
		% within What is your qualification ?	41.9%	12.6%
		Adjusted Residual	6.4	2.3
Total		Count	293	105
		% within What is your qualification ?	22.5%	8.1%

Crosstab

			Does your practice or hospital have a written policy regarding allowing a patient's visitor in ...		Total
			No written policy exists	I do not know	
What is your qualification ?	Board eligible	Count	31	40	90
		% within What is your qualification ?	34.4%	44.4%	100.0%
		Adjusted Residual	-1.0	3.1	
	Board certified	Count	418	321	1022
		% within What is your qualification ?	40.9%	31.4%	100.0%
		Adjusted Residual	2.1	2.2	
	Board eligible and obstetric anesthesia fellowship trained	Count	9	8	22
		% within What is your qualification ?	40.9%	36.4%	100.0%
		Adjusted Residual	.1	.7	
	Board certified and obstetric anesthesia fellowship trained	Count	55	21	167
		% within What is your qualification ?	32.9%	12.6%	100.0%
		Adjusted Residual	-1.8	-5.3	
Total		Count	513	390	1301
		% within What is your qualification ?	39.4%	30.0%	100.0%

Chi-Square Tests

	Value	df	Asymp. Sig. (2-sided)
Pearson Chi-Square	65.517[a]	9	.000
Likelihood Ratio	63.793	9	.000
Linear-by-Linear Association	57.012	1	.000
N of Valid Cases	1301		

a. 2 cells (12.5%) have expected count less than 5. The minimum expected count is 1.78.

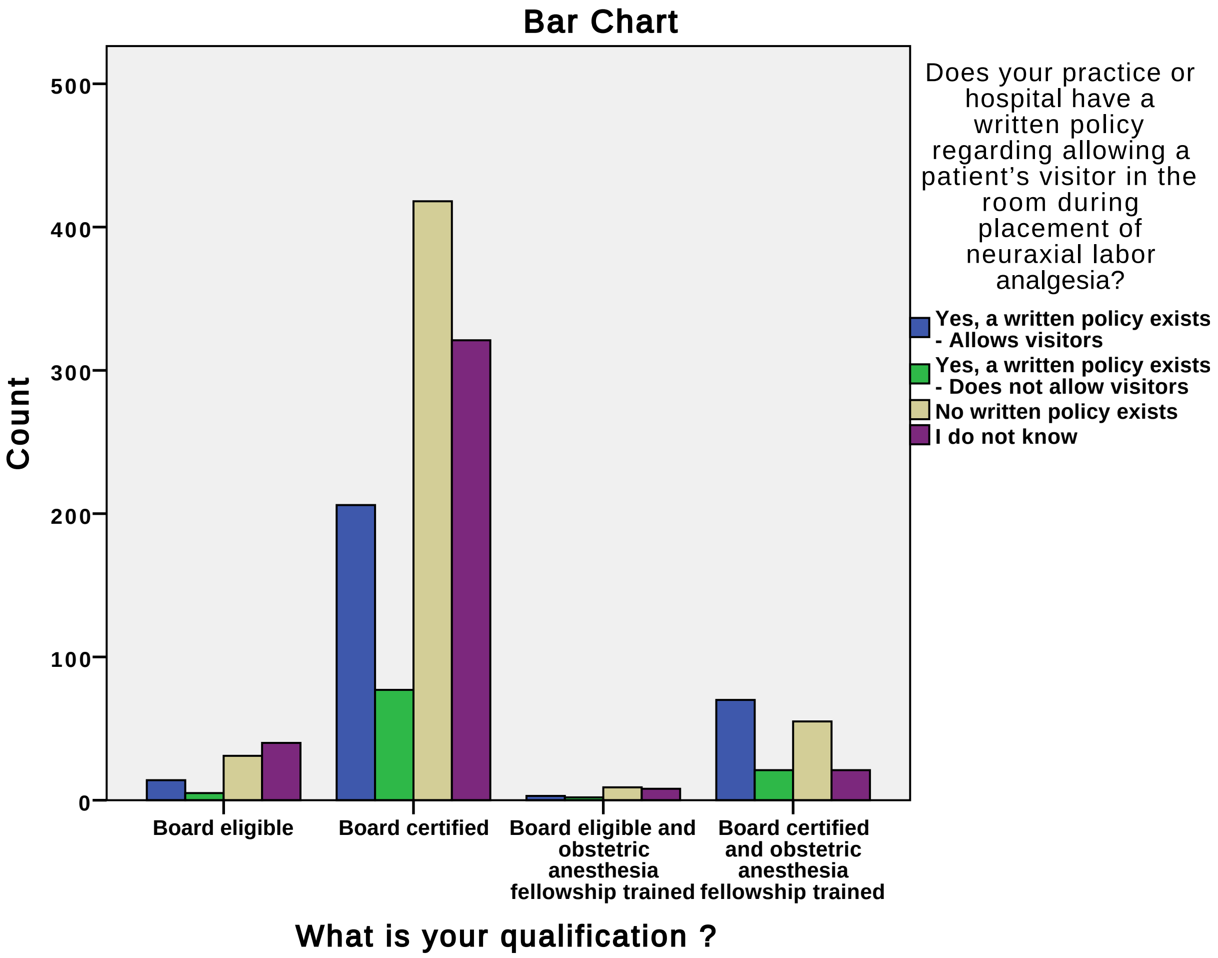

What is your qualification ? * If no policy existed, would you be open to allowing a patient’s visitor in the room during placement of neuraxial labor analgesia?

Crosstab

			If no policy existed, would you be open to allowing a patient's visitor in the room during placement of neuraxial labor analgesia?		
			Yes	No	Total
What is your qualification ?	Board eligible	Count	62	28	90
		% within What is your qualification ?	68.9%	31.1%	100.0%
		Adjusted Residual	-1.4	1.4	
	Board certified	Count	763	259	1022
		% within What is your qualification ?	74.7%	25.3%	100.0%
		Adjusted Residual	-.7	.7	
	Board eligible and obstetric anesthesia fellowship trained	Count	17	5	22
		% within What is your qualification ?	77.3%	22.7%	100.0%
		Adjusted Residual	.2	-.2	
	Board certified and obstetric anesthesia fellowship trained	Count	135	32	167
		% within What is your qualification ?	80.8%	19.2%	100.0%
		Adjusted Residual	1.8	-1.8	
Total		Count	977	324	1301
		% within What is your qualification ?	75.1%	24.9%	100.0%

Chi-Square Tests

	Value	df	Asymp. Sig. (2-sided)
Pearson Chi-Square	4.959[a]	3	.175
Likelihood Ratio	5.045	3	.169
Linear-by-Linear Association	4.693	1	.030
N of Valid Cases	1301		

a. 0 cells (0.0%) have expected count less than 5. The minimum expected count is 5.48.

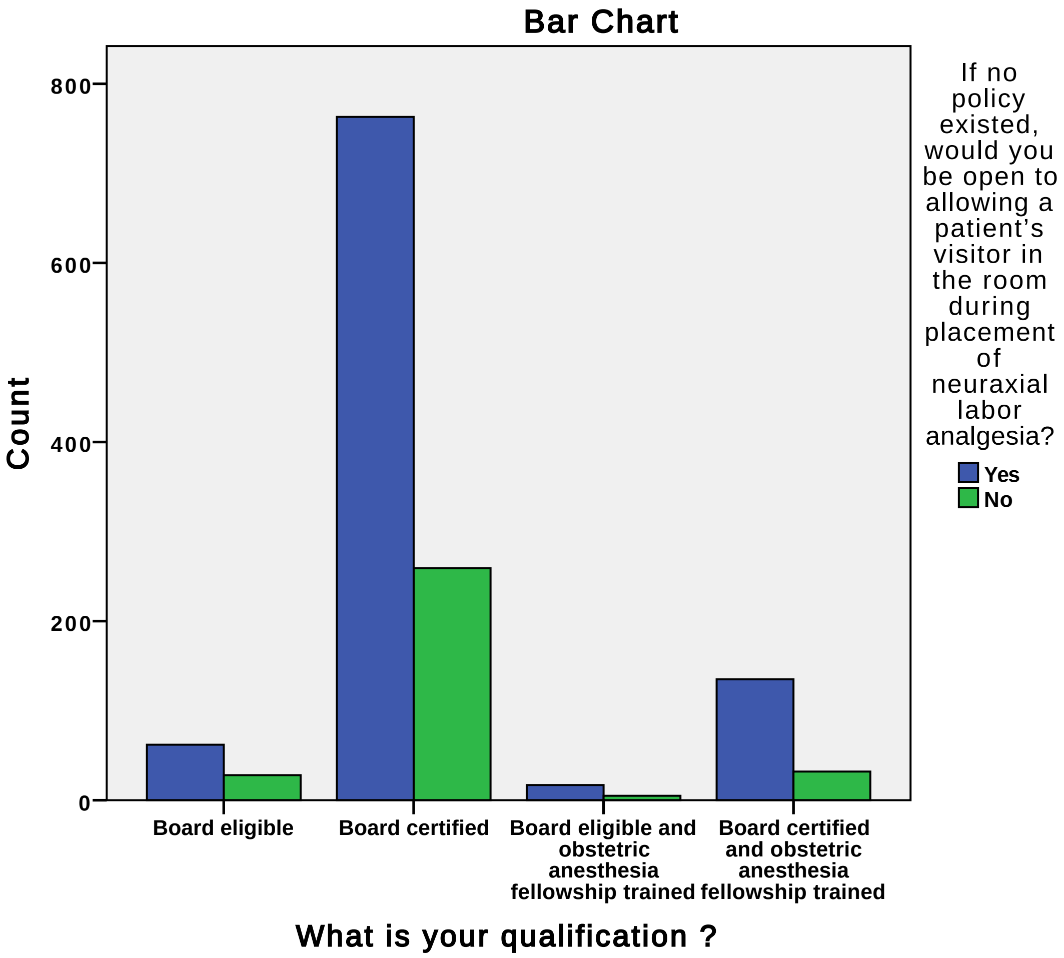

What is your qualification ? * If you allowed a visitor in the room during placement of neuraxial labor analgesia, you would want t he

Crosstab

			If you allowed a visitor in the room during placement of neuraxial labor analgesia, you .	
			Visitor always standing	Visitor always sitting
What is your qualification ?	Board eligible	Count	2	41
		% within What is your qualification ?	3.2%	65.1%
		Adjusted Residual	1.2	-1.3
	Board certified	Count	9	542
		% within What is your qualification ?	1.2%	71.4%
		Adjusted Residual	-1.2	-.8
	Board eligible and obstetric anesthesia fellowship trained	Count	1	15
		% within What is your qualification ?	5.6%	83.3%
		Adjusted Residual	1.5	1.1
	Board certified and obstetric anesthesia fellowship trained	Count	2	103
		% within What is your qualification ?	1.5%	77.4%
		Adjusted Residual	.1	1.5
Total		Count	14	701
		% within What is your qualification ?	1.4%	72.0%

Crosstab

			If you allowed a visitor in the room during ...	Total
			Does not matter	
What is your qualification ?	Board eligible	Count	20	63
		% within What is your qualification ?	31.7%	100.0%
		Adjusted Residual	1.0	
	Board certified	Count	208	759
		% within What is your qualification ?	27.4%	100.0%
		Adjusted Residual	1.2	
	Board eligible and obstetric anesthesia fellowship trained	Count	2	18
		% within What is your qualification ?	11.1%	100.0%
		Adjusted Residual	-1.5	
	Board certified and obstetric anesthesia fellowship trained	Count	28	133
		% within What is your qualification ?	21.1%	100.0%
		Adjusted Residual	-1.5	
Total		Count	258	973
		% within What is your qualification ?	26.5%	100.0%

Chi-Square Tests

	Value	df	Asymp. Sig. (2-sided)
Pearson Chi-Square	9.088[a]	6	.169
Likelihood Ratio	8.384	6	.211
Linear-by-Linear Association	3.416	1	.065
N of Valid Cases	973		

a. 4 cells (33.3%) have expected count less than 5. The minimum expected count is .26.

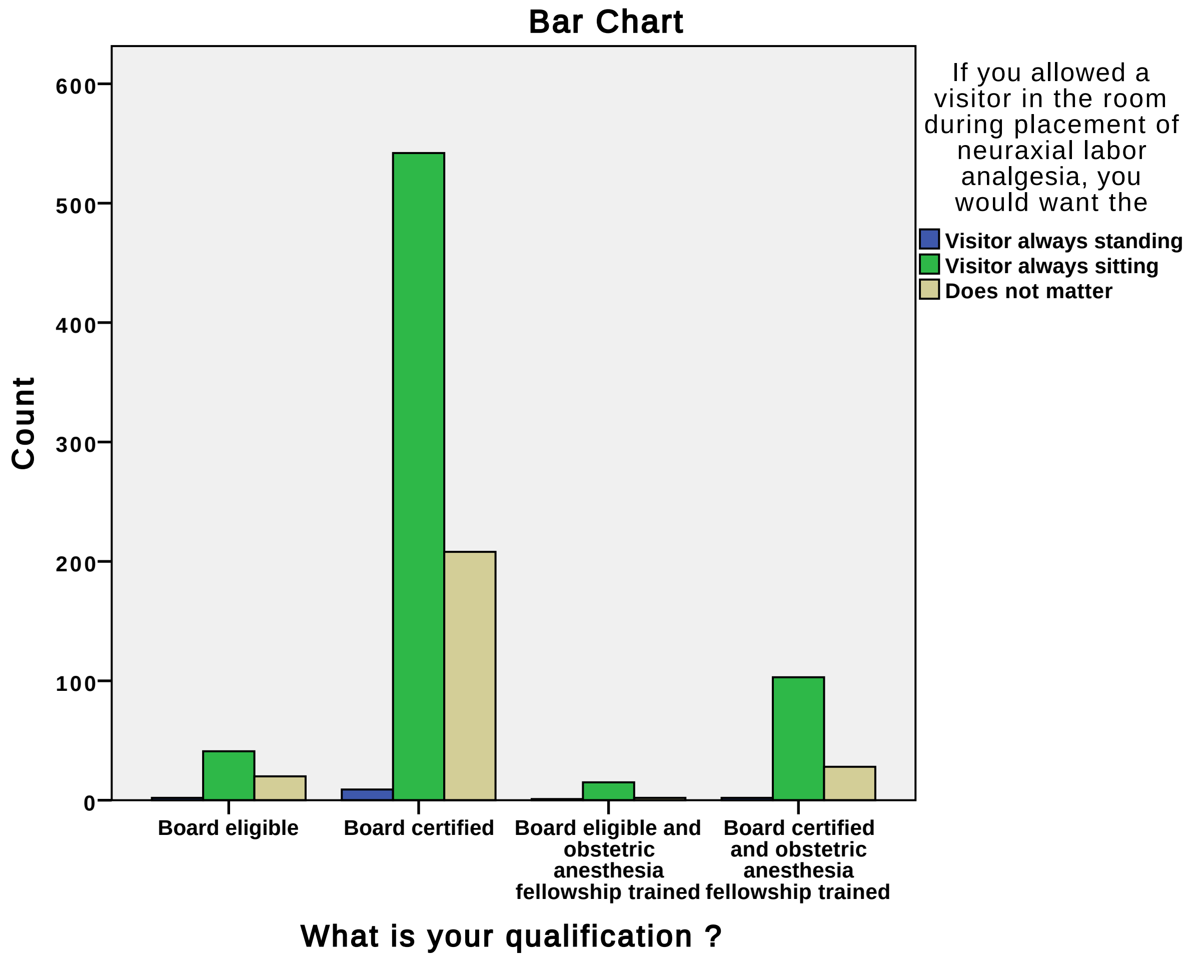

What is your qualification ? * If you allowed a visitor in the room during placement of neuraxial labor analgesia, you would want t he visitor to be positioned such that,

Crosstab

			If you allowed a visitor in the room during placement of neuraxial labor analgesia, you would want the visitor to be ...	
			Visitor has no view of procedure (patient is in between anes	Visitor has partial view of workspace (but cannot see patien
What is your qualification ?	Board eligible	Count	33	17
		% within What is your qualification ?	52.4%	27.0%
		Adjusted Residual	-1.5	1.7
	Board certified	Count	467	140
		% within What is your qualification ?	61.5%	18.4%
		Adjusted Residual	.6	-.9
	Board eligible and obstetric anesthesia fellowship trained	Count	10	4
		% within What is your qualification ?	55.6%	22.2%
		Adjusted Residual	-.5	.4
	Board certified and obstetric anesthesia fellowship trained	Count	84	24
		% within What is your qualification ?	63.2%	18.0%
		Adjusted Residual	.5	-.3
Total		Count	594	185
		% within What is your qualification ?	61.0%	19.0%

Crosstab

			If you allowed a visitor in the room during placement of ...	Total
			Does not matter	
What is your qualification ?	Board eligible	Count	13	63
		% within What is your qualification ?	20.6%	100.0%
		Adjusted Residual	.1	
	Board certified	Count	152	759
		% within What is your qualification ?	20.0%	100.0%
		Adjusted Residual	.1	
	Board eligible and obstetric anesthesia fellowship trained	Count	4	18
		% within What is your qualification ?	22.2%	100.0%
		Adjusted Residual	.2	
	Board certified and obstetric anesthesia fellowship trained	Count	25	133
		% within What is your qualification ?	18.8%	100.0%
		Adjusted Residual	-.4	
Total		Count	194	973
		% within What is your qualification ?	19.9%	100.0%

Chi-Square Tests

	Value	df	Asymp. Sig. (2-sided)
Pearson Chi-Square	3.539[a]	6	.739
Likelihood Ratio	3.333	6	.766
Linear-by-Linear Association	.479	1	.489
N of Valid Cases	973		

a. 2 cells (16.7%) have expected count less than 5. The minimum expected count is 3.42.

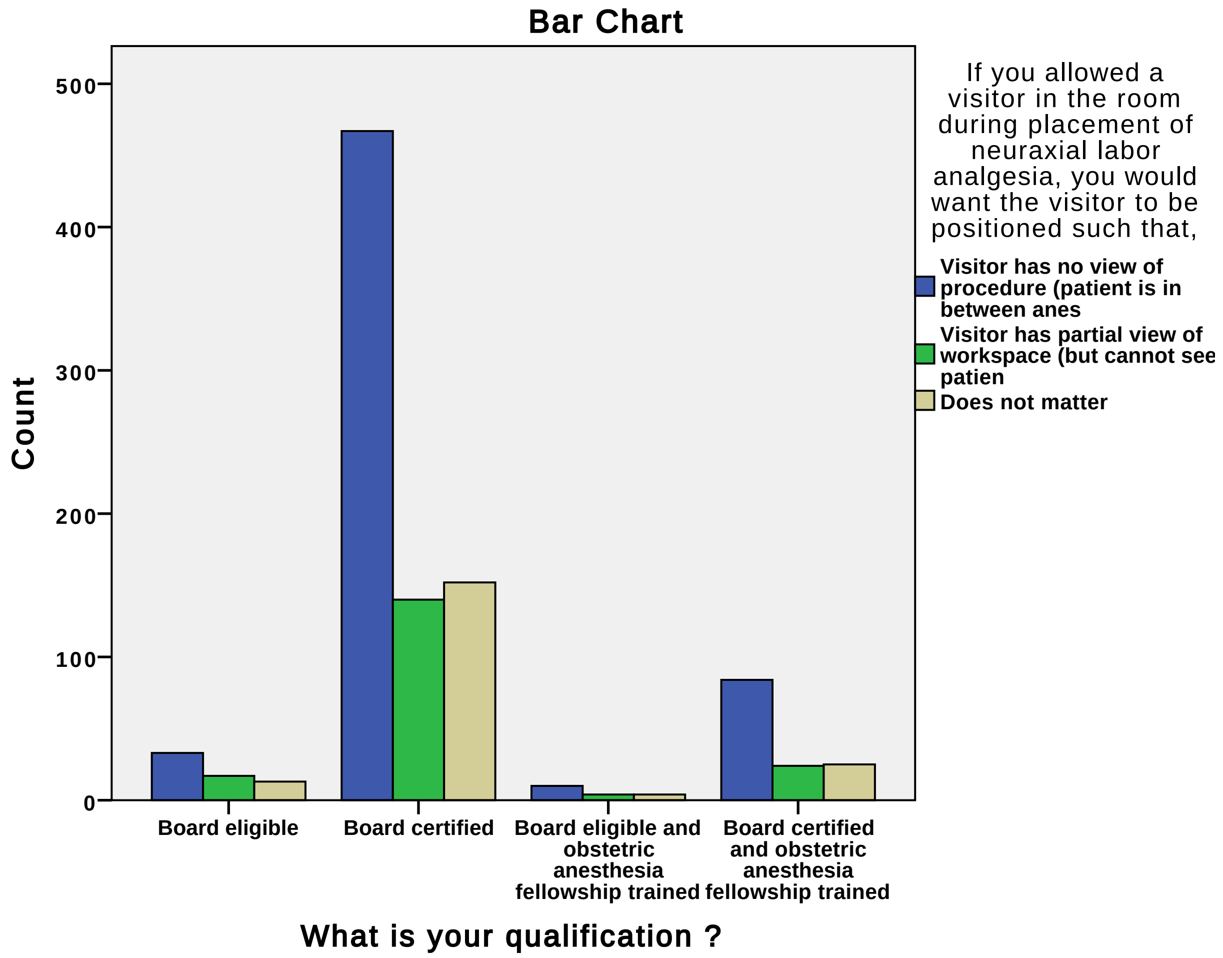

What is your qualification ? * What would be your single most important reason for allowing a patient's visitor in the room during placement of neuraxial labor analgesia?

Crosstab

			What would be your single most important reason for allowing a patient’s visitor in ..	
			It would likely reduce patient's anxiety	It would likely reduce visitor's anxiety (for e. g. if visito
What is your qualification ?	Board eligible	Count	40	5
		% within What is your qualification ?	63.5%	7.9%
		Adjusted Residual	-.2	1.1
	Board certified	Count	503	36
		% within What is your qualification ?	66.3%	4.7%
		Adjusted Residual	2.0	-.8
	Board eligible and obstetric anesthesia fellowship trained	Count	13	0
		% within What is your qualification ?	72.2%	0.0%
		Adjusted Residual	.7	-1.0
	Board certified and obstetric anesthesia fellowship trained	Count	73	8
		% within What is your qualification ?	54.9%	6.0%
		Adjusted Residual	-2.5	.6
Total		Count	629	49
		% within What is your qualification ?	64.6%	5.0%

Crosstab

			What would be your single most important reason for allowing a patient’s visitor in the room …		Total
			Visitor's assistance needed (for e. g. if visitor were a doul	To fulfill patient's request	
What is your qualification ?	Board eligible	Count	2	16	63
		% within What is your qualification ?	3.2%	25.4%	100.0%
		Adjusted Residual	-.3	-.2	
	Board certified	Count	26	194	759
		% within What is your qualification ?	3.4%	25.6%	100.0%
		Adjusted Residual	-1.2	-1.3	
	Board eligible and obstetric anesthesia fellowship trained	Count	0	5	18
		% within What is your qualification ?	0.0%	27.8%	100.0%
		Adjusted Residual	-.9	.1	
	Board certified and obstetric anesthesia fellowship trained	Count	9	43	133
		% within What is your qualification ?	6.8%	32.3%	100.0%
		Adjusted Residual	1.9	1.6	
Total		Count	37	258	973
		% within What is your qualification ?	3.8%	26.5%	100.0%

Chi-Square Tests

	Value	df	Asymp. Sig. (2-sided)
Pearson Chi-Square	10.889[a]	9	.283
Likelihood Ratio	11.729	9	.229
Linear-by-Linear Association	4.215	1	.040
N of Valid Cases	973		

a. 5 cells (31.3%) have expected count less than 5. The minimum expected count is .68.

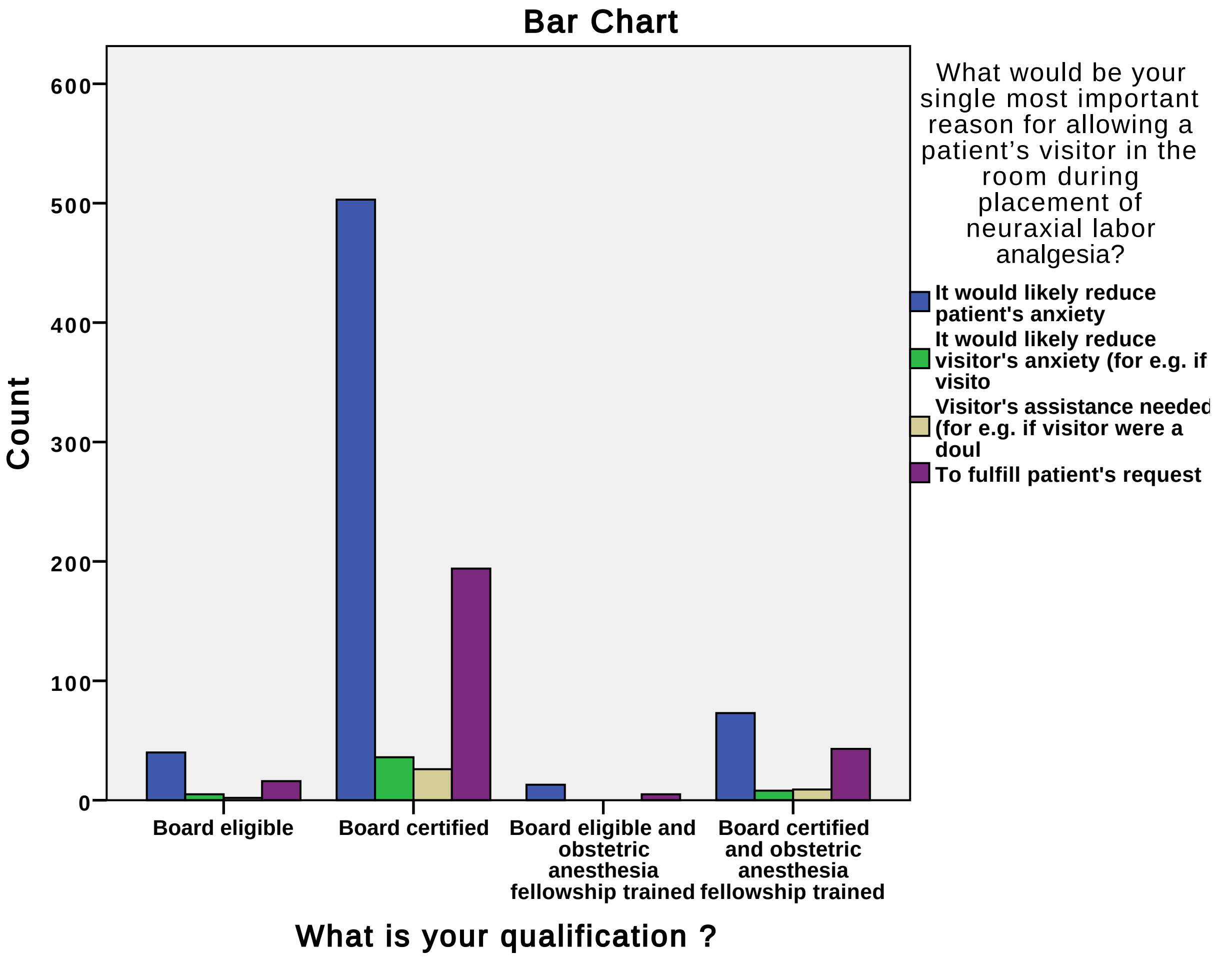

What is your qualification ? * How often has another non anesthesia member of the labor and delivery team (for example obstetrician or nurse) attempted to influence your decision to have a visitor present during placement of neuraxial labor analgesia?

Crosstab

			How often has another non anesthesia member of the labor and delivery team (for example obstetrician or nurse) attempted to influence your decision to have a visitor .	
			Rarely (< 5 %)	Occasionally (5 % - 40 %)
What is your qualification ?	Board eligible	Count	50	7
		% within What is your qualification ?	79.4%	11.1%
		Adjusted Residual	1.1	-1.5
	Board certified	Count	562	141
		% within What is your qualification ?	74.0%	18.6%
		Adjusted Residual	.5	.7
	Board eligible and obstetric anesthesia fellowship trained	Count	9	5
		% within What is your qualification ?	50.0%	27.8%
		Adjusted Residual	-2.3	1.1
	Board certified and obstetric anesthesia fellowship trained	Count	96	23
		% within What is your qualification ?	72.2%	17.3%
		Adjusted Residual	-.4	-.3
Total		Count	717	176
		% within What is your qualification ?	73.7%	18.1%

Crosstab

			How often has another non anesthesia member of the labor and delivery team ...	
			Often > 40 %	Total
What is your qualification ?	Board eligible	Count	6	63
		% within What is your qualification ?	9.5%	100.0%
		Adjusted Residual	.4	
	Board certified	Count	56	759
		% within What is your qualification ?	7.4%	100.0%
		Adjusted Residual	-1.8	
	Board eligible and obstetric anesthesia fellowship trained	Count	4	18
		% within What is your qualification ?	22.2%	100.0%
		Adjusted Residual	2.2	
	Board certified and obstetric anesthesia fellowship trained	Count	14	133
		% within What is your qualification ?	10.5%	100.0%
		Adjusted Residual	1.0	
Total		Count	80	973
		% within What is your qualification ?	8.2%	100.0%

Chi-Square Tests

	Value	df	Asymp. Sig. (2-sided)
Pearson Chi-Square	10.414[a]	6	.108
Likelihood Ratio	9.294	6	.158
Linear-by-Linear Association	1.761	1	.184
N of Valid Cases	973		

a. 2 cells (16.7%) have expected count less than 5. The minimum expected count is 1.48.

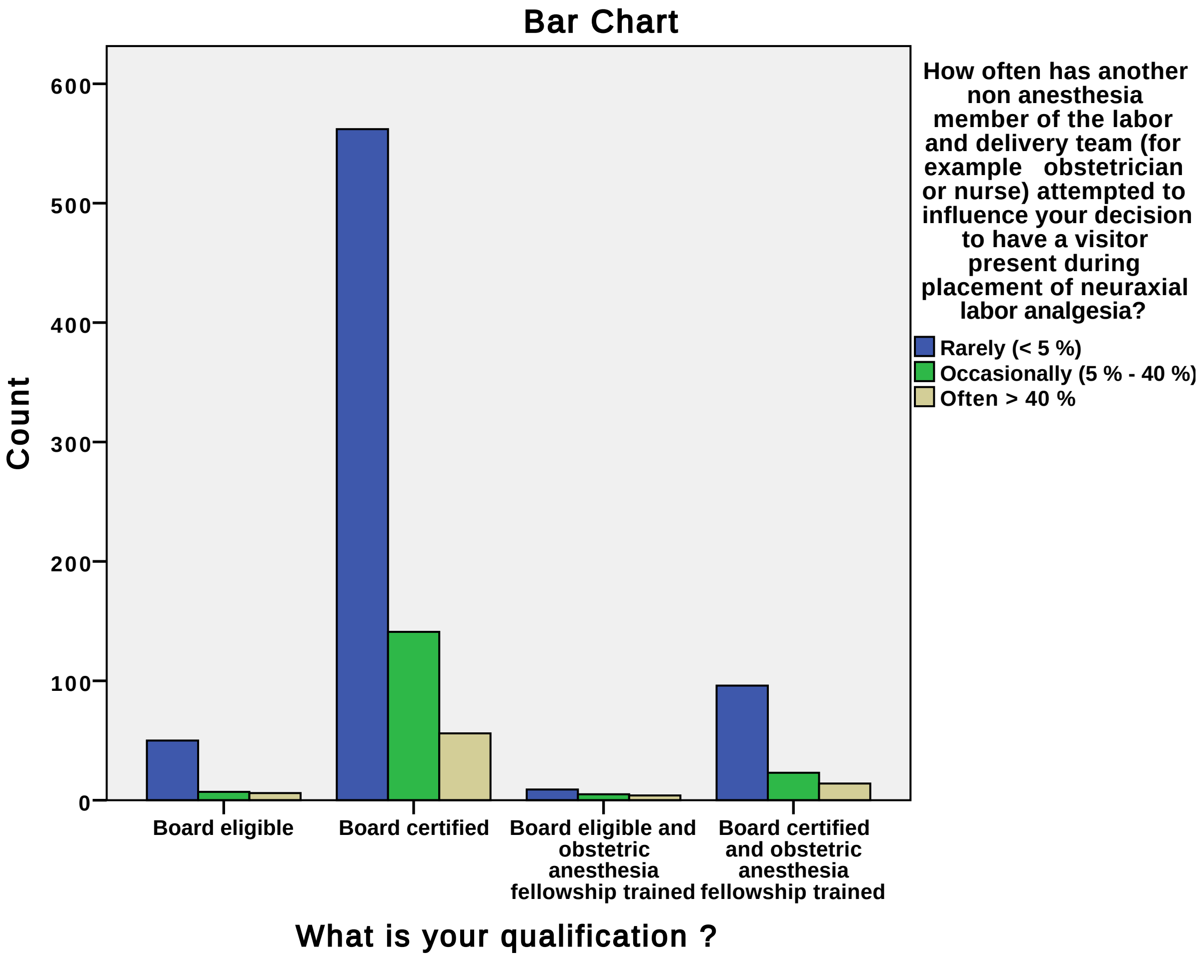

What is your qualification ? * How often has another non anesthes ia member of the labor and delivery team (for example obstetrici an or nurse) attempted to influence your decision to NOT have a visitor present during placement of neuraxial labor analgesia?

Crosstab

			How often has another non anesthesia member of the labor and delivery team (for example obstetrician or nurse) attempted to influence your decision to NOT have a visitor .	
			Rarely < 5 %	Occasionally 5 - 40 %
What is your qualification ?	Board eligible	Count	22	3
		% within What is your qualification ?	78.6%	10.7%
		Adjusted Residual	.0	-.8
	Board certified	Count	203	42
		% within What is your qualification ?	79.0%	16.3%
		Adjusted Residual	.4	.5
	Board eligible and obstetric anesthesia fellowship trained	Count	3	2
		% within What is your qualification ?	60.0%	40.0%
		Adjusted Residual	-1.0	1.5
	Board certified and obstetric anesthesia fellowship trained	Count	25	4
		% within What is your qualification ?	78.1%	12.5%
		Adjusted Residual	-.1	-.5
Total		Count	253	51
		% within What is your qualification ?	78.6%	15.8%

Crosstab

			How often has another non anesthesia member of the labor and delivery team ...	
			Often > 40 %	Total
What is your qualification ?	Board eligible	Count	3	28
		% within What is your qualification ?	10.7%	100.0%
		Adjusted Residual	1.2	
	Board certified	Count	12	257
		% within What is your qualification ?	4.7%	100.0%
		Adjusted Residual	-1.4	
	Board eligible and obstetric anesthesia fellowship trained	Count	0	5
		% within What is your qualification ?	0.0%	100.0%
		Adjusted Residual	-.5	
	Board certified and obstetric anesthesia fellowship trained	Count	3	32
		% within What is your qualification ?	9.4%	100.0%
		Adjusted Residual	1.0	
Total		Count	18	322
		% within What is your qualification ?	5.6%	100.0%

Chi-Square Tests

	Value	df	Asymp. Sig. (2-sided)
Pearson Chi-Square	5.604[a]	6	.469
Likelihood Ratio	5.029	6	.540
Linear-by-Linear Association	.077	1	.782
N of Valid Cases	322		

a. 6 cells (50.0%) have expected count less than 5. The minimum expected count is .28.

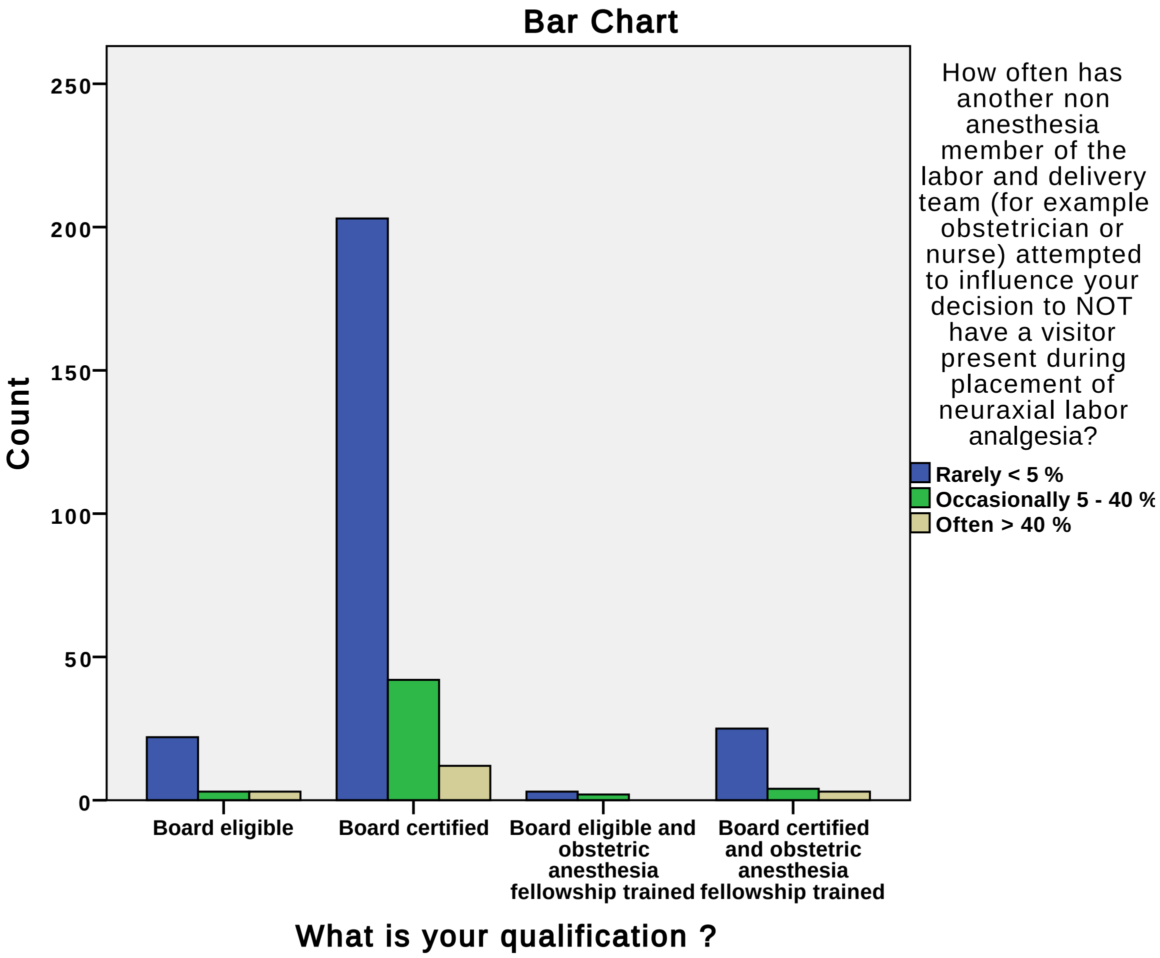

What is your qualification ? * What would be your single most important reason for NOT allowing a patient's visitor in the room during placement of neuraxial labor analgesia?

Crosstab

			What would be your single most important reason for NOT allowing a patient's visitor in ..	
			Increase in anesthesiologist's stress	Possible interference by visitor(for e.g. comments made if d
What is your qualification ?	Board eligible	Count	4	16
		% within What is your qualification ?	14.3%	57.1%
		Adjusted Residual	1.5	1.0
	Board certified	Count	16	125
		% within What is your qualification ?	6.2%	48.6%
		Adjusted Residual	-1.3	.1
	Board eligible and obstetric anesthesia fellowship trained	Count	0	3
		% within What is your qualification ?	0.0%	60.0%
		Adjusted Residual	-.6	.5
	Board certified and obstetric anesthesia fellowship trained	Count	3	12
		% within What is your qualification ?	9.4%	37.5%
		Adjusted Residual	.5	-1.3
Total		Count	23	156
		% within What is your qualification ?	7.1%	48.4%

Crosstab

			What would be your single most important reason for NOT allowing a patient's visitor in ...		Total
			Concern about visitor (for e.g. passing out or not being ab	Medico-legal concerns	
What is your qualification ?	Board eligible	Count	6	2	28
		% within What is your qualification ?	21.4%	7.1%	100.0%
		Adjusted Residual	-1.4	-.8	
	Board certified	Count	87	29	257
		% within What is your qualification ?	33.9%	11.3%	100.0%
		Adjusted Residual	.7	-.2	
	Board eligible and obstetric anesthesia fellowship trained	Count	1	1	5
		% within What is your qualification ?	20.0%	20.0%	100.0%
		Adjusted Residual	-.6	.6	
	Board certified and obstetric anesthesia fellowship trained	Count	12	5	32
		% within What is your qualification ?	37.5%	15.6%	100.0%
		Adjusted Residual	.6	.8	
Total		Count	106	37	322
		% within What is your qualification ?	32.9%	11.5%	100.0%

Chi-Square Tests

	Value	df	Asymp. Sig. (2-sided)
Pearson Chi-Square	7.161[a]	9	.620
Likelihood Ratio	7.249	9	.611
Linear-by-Linear Association	2.316	1	.128
N of Valid Cases	322		

a. 8 cells (50.0%) have expected count less than 5. The minimum expected count is .36.

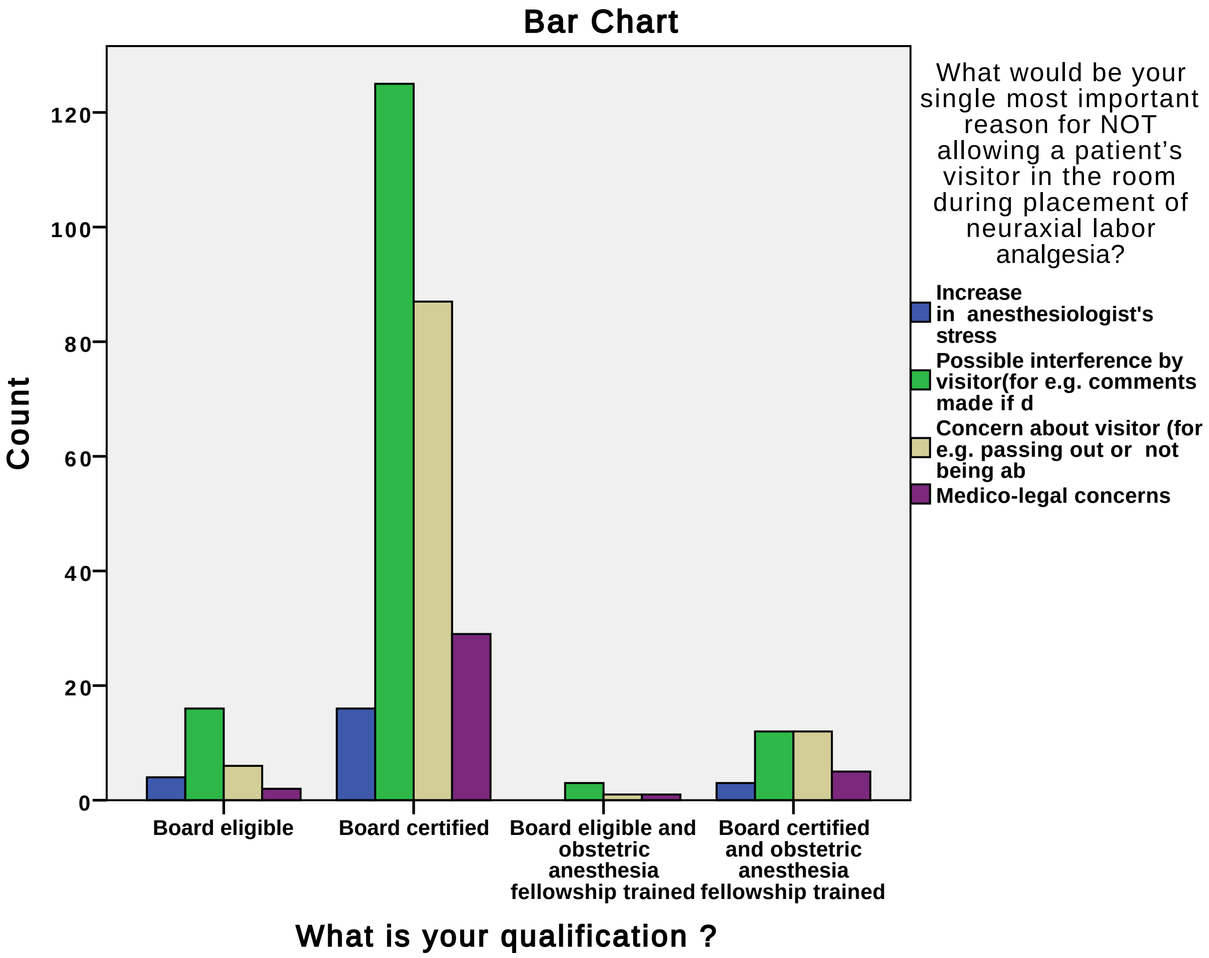

Which region of USA do you practice in ? * What is your preferred technique for neuraxial labor analgesia in an otherwise healthy p arturient?

Crosstab

			What is your preferred technique for neuraxial labor analgesia in an otherwise healthy parturient?		
			Epidural analgesia	Combined spinal epidural analgesia	Total
Which region of USA do you practice in ?	Northeast	Count	417	128	545
		% within Which region of USA do you practice in ?	76.5%	23.5%	100.0%
		Adjusted Residual	-1.1	1.1	
	Midwest	Count	218	67	285
		% within Which region of USA do you practice in ?	76.5%	23.5%	100.0%
		Adjusted Residual	-.7	.7	
	South	Count	197	44	241
		% within Which region of USA do you practice in ?	81.7%	18.3%	100.0%
		Adjusted Residual	1.5	-1.5	
	West	Count	183	47	230
		% within Which region of USA do you practice in ?	79.6%	20.4%	100.0%
		Adjusted Residual	.6	-.6	
Total		Count	1015	286	1301
		% within Which region of USA do you practice in ?	78.0%	22.0%	100.0%

Chi-Square Tests

	Value	df	Asymp. Sig. (2-sided)
Pearson Chi-Square	3.377[a]	3	.337
Likelihood Ratio	3.453	3	.327
Linear-by-Linear Association	2.038	1	.153
N of Valid Cases	1301		

a. 0 cells (0.0%) have expected count less than 5. The minimum expected count is 50.56.

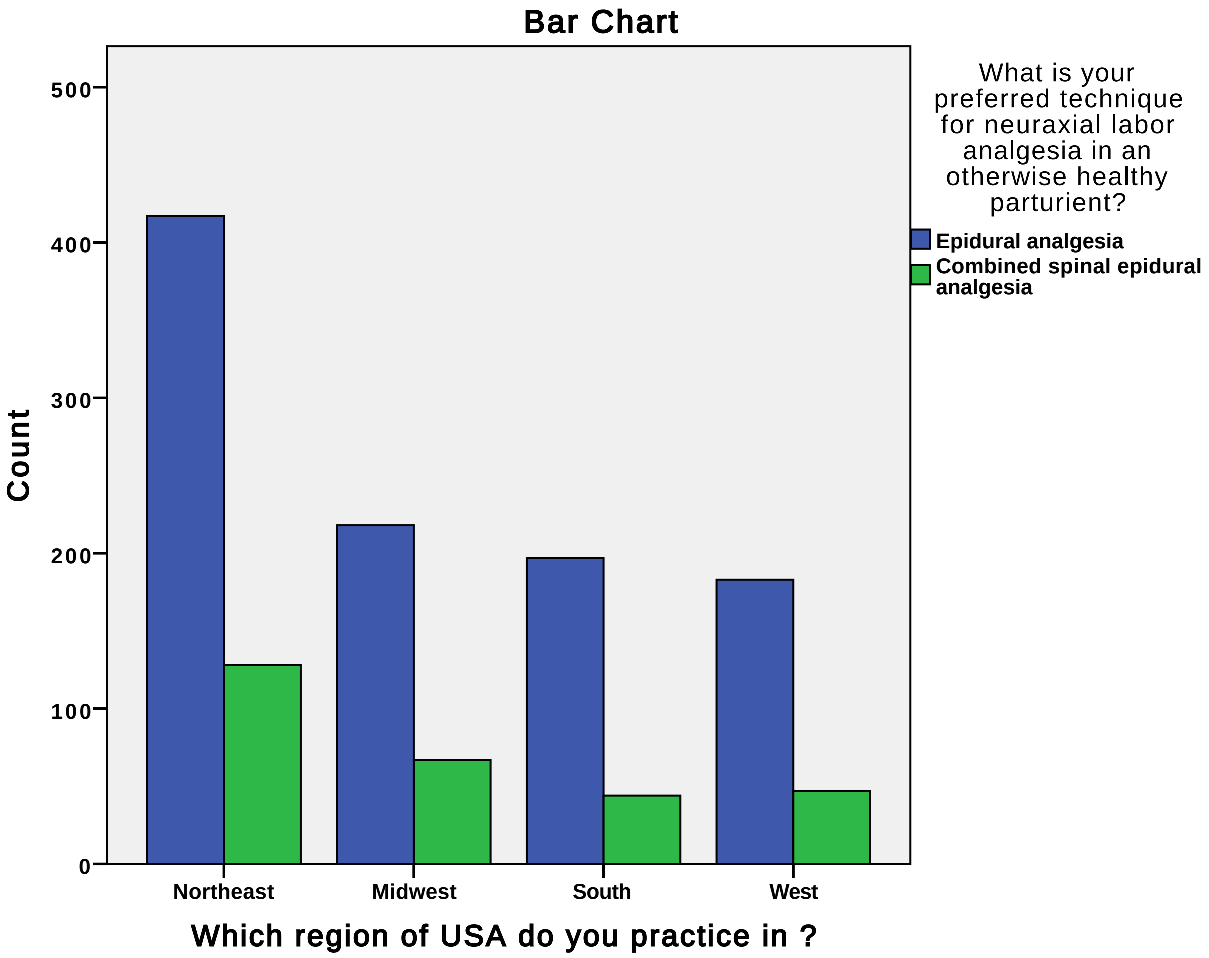

Which region of USA do you practice in ? * Does your practice or hospital have a written policy regarding allowing a patient's visitor in the room during placement of neuraxial labor analgesia?

Crosstab

			Does your practice or hospital have a written policy regarding allowing a patient's visitor in the room during placement of neuraxial labor ..		
			Yes, a written policy exists - Allows visitors	Yes, a written policy exists - Does not allow visitors	No written policy exists
Which region of USA do you practice in ?	Northeast	Count	99	54	227
		% within Which region of USA do you practice in ?	18.2%	9.9%	41.7%
		Adjusted Residual	-3.2	2.1	1.4
	Midwest	Count	67	26	123
		% within Which region of USA do you practice in ?	23.5%	9.1%	43.2%
		Adjusted Residual	.5	.7	1.5
	South	Count	60	18	79
		% within Which region of USA do you practice in ?	24.9%	7.5%	32.8%
		Adjusted Residual	1.0	-.4	-2.3
	West	Count	67	7	84
		% within Which region of USA do you practice in ?	29.1%	3.0%	36.5%
		Adjusted Residual	2.6	-3.1	-1.0
Total		Count	293	105	513
		% within Which region of USA do you practice in ?	22.5%	8.1%	39.4%

Crosstab

			Does your practice or hospital have a ...	
			I do not know	Total
Which region of USA do you practice in ?	Northeast	Count	165	545
		% within Which region of USA do you practice in ?	30.3%	100.0%
		Adjusted Residual	.2	
	Midwest	Count	69	285
		% within Which region of USA do you practice in ?	24.2%	100.0%
		Adjusted Residual	-2.4	
	South	Count	84	241
		% within Which region of USA do you practice in ?	34.9%	100.0%
		Adjusted Residual	1.8	
	West	Count	72	230
		% within Which region of USA do you practice in ?	31.3%	100.0%
		Adjusted Residual	.5	
Total		Count	390	1301
		% within Which region of USA do you practice in ?	30.0%	100.0%

Chi-Square Tests

	Value	df	Asymp. Sig. (2-sided)
Pearson Chi-Square	29.870[a]	9	.000
Likelihood Ratio	32.158	9	.000
Linear-by-Linear Association	2.290	1	.130
N of Valid Cases	1301		

a. 0 cells (0.0%) have expected count less than 5. The minimum expected count is 18.56.

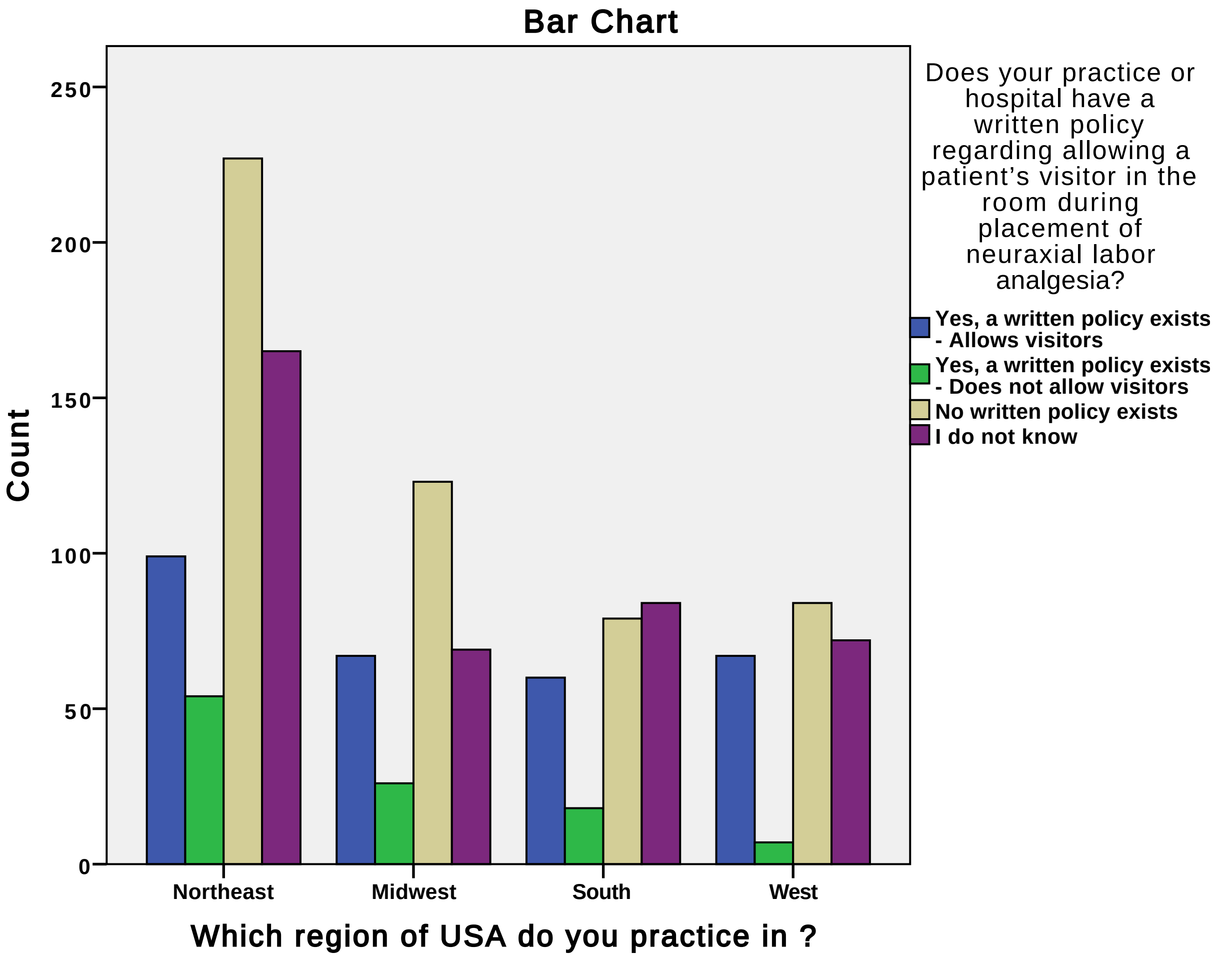

Which region of USA do you practice in ? * If no policy existed, would you be open to allowing a patient's visitor in the room during placement of neuraxial labor analgesia?

Crosstab

			If no policy existed, would you be open to allowing a patient's visitor in the room during placement of neuraxial labor analgesia?		
			Yes	No	Total
Which region of USA do you practice in ?	Northeast	Count	361	184	545
		% within Which region of USA do you practice in ?	66.2%	33.8%	100.0%
		Adjusted Residual	-6.3	6.3	
	Midwest	Count	233	52	285
		% within Which region of USA do you practice in ?	81.8%	18.2%	100.0%
		Adjusted Residual	2.9	-2.9	
	South	Count	181	60	241
		% within Which region of USA do you practice in ?	75.1%	24.9%	100.0%
		Adjusted Residual	.0	.0	
	West	Count	202	28	230
		% within Which region of USA do you practice in ?	87.8%	12.2%	100.0%
		Adjusted Residual	4.9	-4.9	
Total		Count	977	324	1301
		% within Which region of USA do you practice in ?	75.1%	24.9%	100.0%

Chi-Square Tests

	Value	df	Asymp. Sig. (2-sided)
Pearson Chi-Square	49.549[a]	3	.000
Likelihood Ratio	51.780	3	.000
Linear-by-Linear Association	36.153	1	.000
N of Valid Cases	1301		

a. 0 cells (0.0%) have expected count less than 5. The minimum expected count is 57.28.

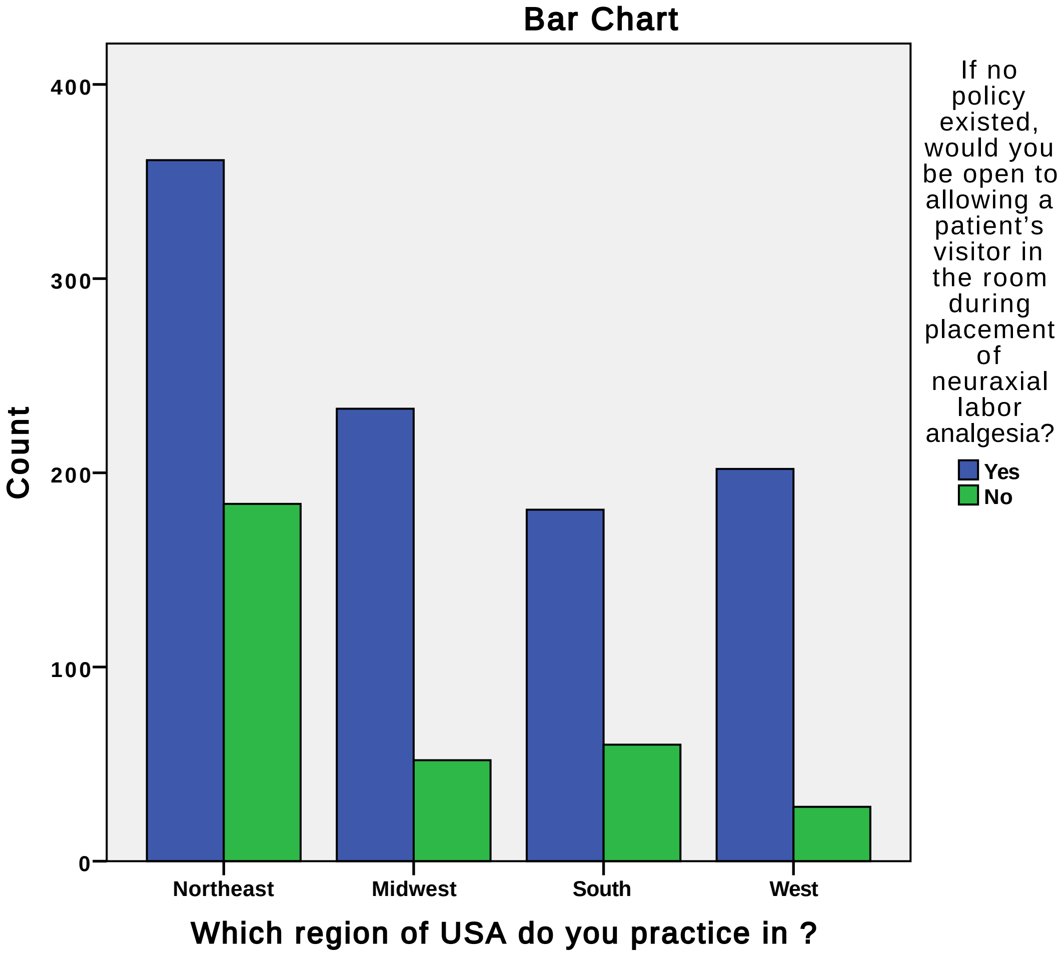

Which region of USA do you practice in ? * If you allowed a visitor in the room during placement of neuraxial labor analgesia, you w ould want the

Crosstab

			If you allowed a visitor in the room during placement of neuraxial labor analgesia, you would want the		
			Visitor always standing	Visitor always sitting	Does not matter
Which region of USA do you practice in ?	Northeast	Count	4	245	110
		% within Which region of USA do you practice in ?	1.1%	68.2%	30.6%
		Adjusted Residual	-.7	-2.0	2.2
	Midwest	Count	4	166	62
		% within Which region of USA do you practice in ?	1.7%	71.6%	26.7%
		Adjusted Residual	.4	-.2	.1
	South	Count	6	129	46
		% within Which region of USA do you practice in ?	3.3%	71.3%	25.4%
		Adjusted Residual	2.3	-.3	-.4
	West	Count	0	161	40
		% within Which region of USA do you practice in ?	0.0%	80.1%	19.9%
		Adjusted Residual	-1.9	2.9	-2.4
Total		Count	14	701	258
		% within Which region of USA do you practice in ?	1.4%	72.0%	26.5%

Crosstab

			Total
Which region of USA do you practice in ?	Northeast	Count	359
		% within Which region of USA do you practice in ?	100.0%
		Adjusted Residual	
	Midwest	Count	232
		% within Which region of USA do you practice in ?	100.0%
		Adjusted Residual	
	South	Count	181
		% within Which region of USA do you practice in ?	100.0%
		Adjusted Residual	
	West	Count	201
		% within Which region of USA do you practice in ?	100.0%
		Adjusted Residual	
Total		Count	973
		% within Which region of USA do you practice in ?	100.0%

Chi-Square Tests

	Value	df	Asymp. Sig. (2-sided)
Pearson Chi-Square	15.974[a]	6	.014
Likelihood Ratio	17.832	6	.007
Linear-by-Linear Association	6.345	1	.012
N of Valid Cases	973		

a. 3 cells (25.0%) have expected count less than 5. The minimum expected count is 2.60.

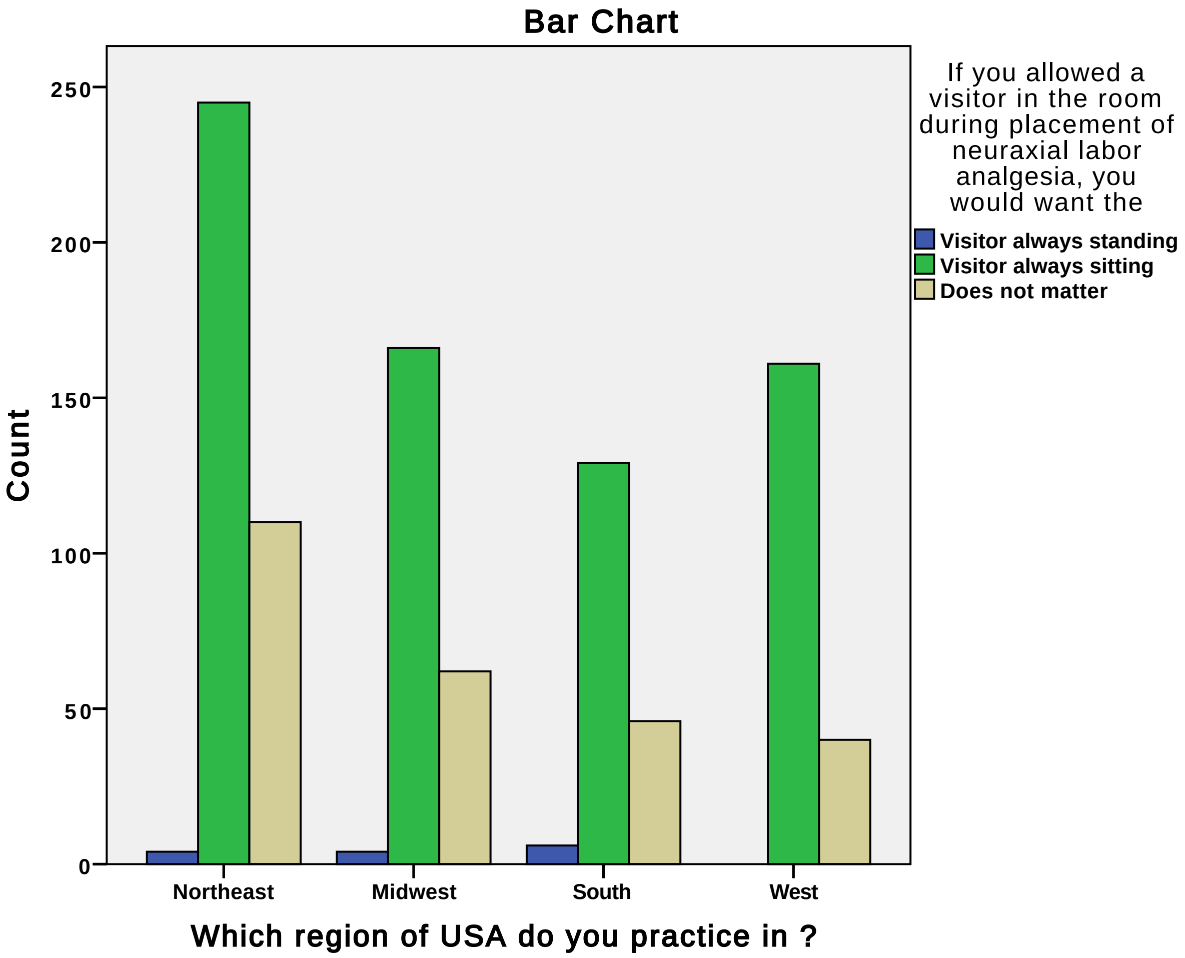

Which region of USA do you practice in ? * If you allowed a visitor in the room during placement of neuraxial labor analgesia, you w ould want the visitor to be positioned such that,

Crosstab

			If you allowed a visitor in the room during placement of neuraxial labor analgesia, you would want the visitor to be positioned such that,		
			Visitor has no view of procedure (patient is in between anes	Visitor has partial view of workspace (but cannot see patien	Does not matter
Which region of USA do you practice in ?	Northeast	Count	234	71	54
		% within Which region of USA do you practice in ?	65.2%	19.8%	15.0%
		Adjusted Residual	2.0	.5	-2.9
	Midwest	Count	128	45	59
		% within Which region of USA do you practice in ?	55.2%	19.4%	25.4%
		Adjusted Residual	-2.1	.2	2.4
	South	Count	116	36	29
		% within Which region of USA do you practice in ?	64.1%	19.9%	16.0%
		Adjusted Residual	.9	.3	-1.5
	West	Count	116	33	52
		% within Which region of USA do you practice in ?	57.7%	16.4%	25.9%
		Adjusted Residual	-1.1	-1.1	2.4
Total		Count	594	185	194
		% within Which region of USA do you practice in ?	61.0%	19.0%	19.9%

Crosstab

			Total
Which region of USA do you practice in ?	Northeast	Count	359
		% within Which region of USA do you practice in ?	100.0%
		Adjusted Residual	
	Midwest	Count	232
		% within Which region of USA do you practice in ?	100.0%
		Adjusted Residual	
	South	Count	181
		% within Which region of USA do you practice in ?	100.0%
		Adjusted Residual	
	West	Count	201
		% within Which region of USA do you practice in ?	100.0%
		Adjusted Residual	
Total		Count	973
		% within Which region of USA do you practice in ?	100.0%

Chi-Square Tests

	Value	df	Asymp. Sig. (2-sided)
Pearson Chi-Square	16.638[a]	6	.011
Likelihood Ratio	16.609	6	.011
Linear-by-Linear Association	3.930	1	.047
N of Valid Cases	973		

a. 0 cells (0.0%) have expected count less than 5. The minimum expected count is 34.41.

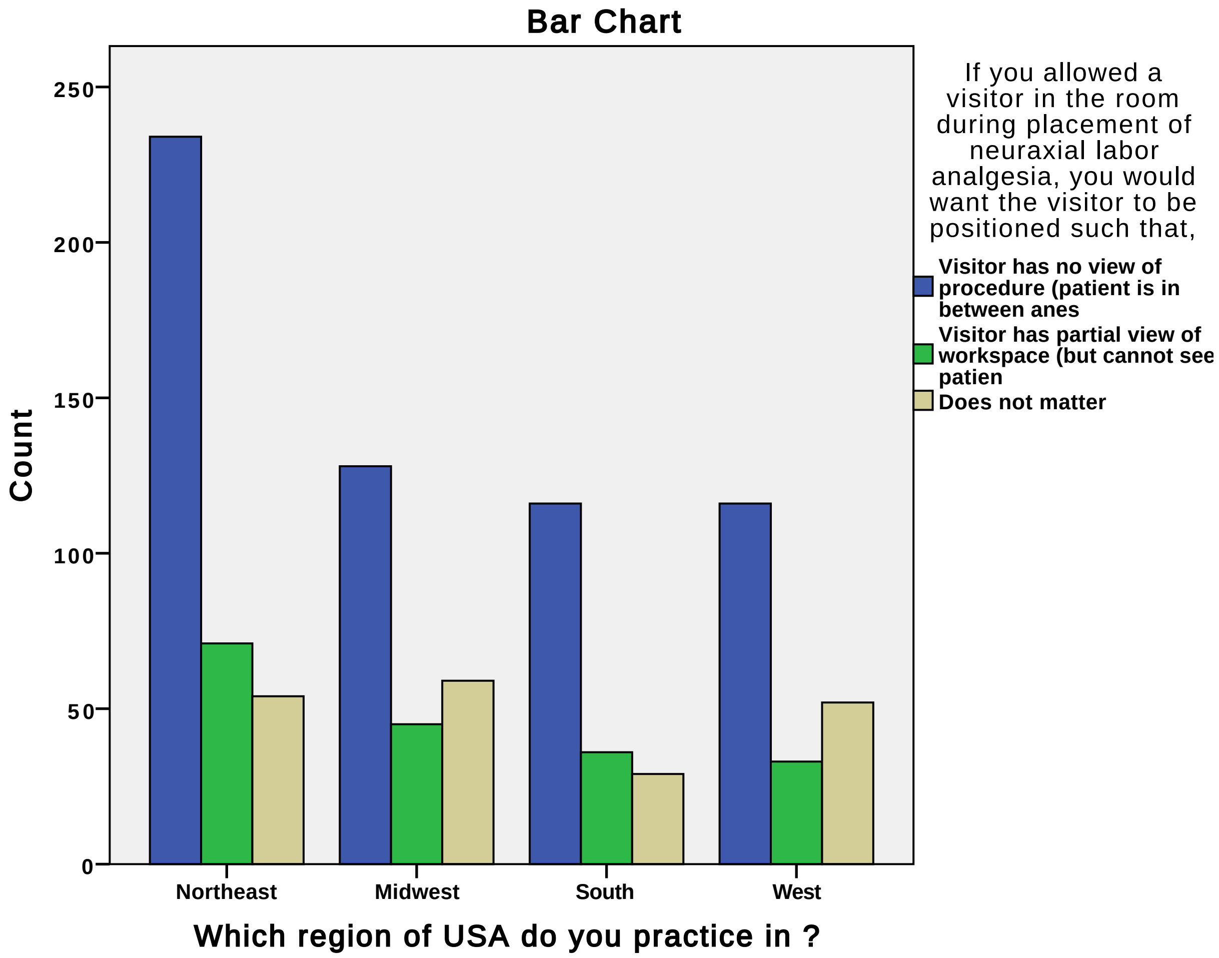

Which region of USA do you practice in ? * What would be your single most important reason for allowing a patient's visitor in the room during placement of neuraxial labor analgesia?

Crosstab

			What would be your single most important reason for allowing a patient’s visitor in the room during placement of neuraxial labor …		
			It would likely reduce patient's anxiety	It would likely reduce visitor's anxiety (for e. g. if visito	Visitor's assistance needed (for e. g. if visitor were a doul
Which region of USA do you practice in ?	Northeast	Count	230	21	19
		% within Which region of USA do you practice in ?	64.1%	5.8%	5.3%
		Adjusted Residual	-.3	.9	1.9
	Midwest	Count	140	12	7
		% within Which region of USA do you practice in ?	60.3%	5.2%	3.0%
		Adjusted Residual	-1.6	.1	-.7
	South	Count	120	8	7
		% within Which region of USA do you practice in ?	66.3%	4.4%	3.9%
		Adjusted Residual	.5	-.4	.1
	West	Count	139	8	4
		% within Which region of USA do you practice in ?	69.2%	4.0%	2.0%
		Adjusted Residual	1.5	-.8	-1.5
Total		Count	629	49	37
		% within Which region of USA do you practice in ?	64.6%	5.0%	3.8%

Crosstab

			What would be your single most important ...	Total
			To fulfill patient's request	
Which region of USA do you practice in ?	Northeast	Count	89	359
		% within Which region of USA do you practice in ?	24.8%	100.0%
		Adjusted Residual	-.9	
	Midwest	Count	73	232
		% within Which region of USA do you practice in ?	31.5%	100.0%
		Adjusted Residual	2.0	
	South	Count	46	181
		% within Which region of USA do you practice in ?	25.4%	100.0%
		Adjusted Residual	-.4	
	West	Count	50	201
		% within Which region of USA do you practice in ?	24.9%	100.0%
		Adjusted Residual	-.6	
Total		Count	258	973
		% within Which region of USA do you practice in ?	26.5%	100.0%

Chi-Square Tests

	Value	df	Asymp. Sig. (2-sided)
Pearson Chi-Square	9.496[a]	9	.393
Likelihood Ratio	9.575	9	.386
Linear-by-Linear Association	.729	1	.393
N of Valid Cases	973		

a. 0 cells (0.0%) have expected count less than 5. The minimum expected count is 6.88.

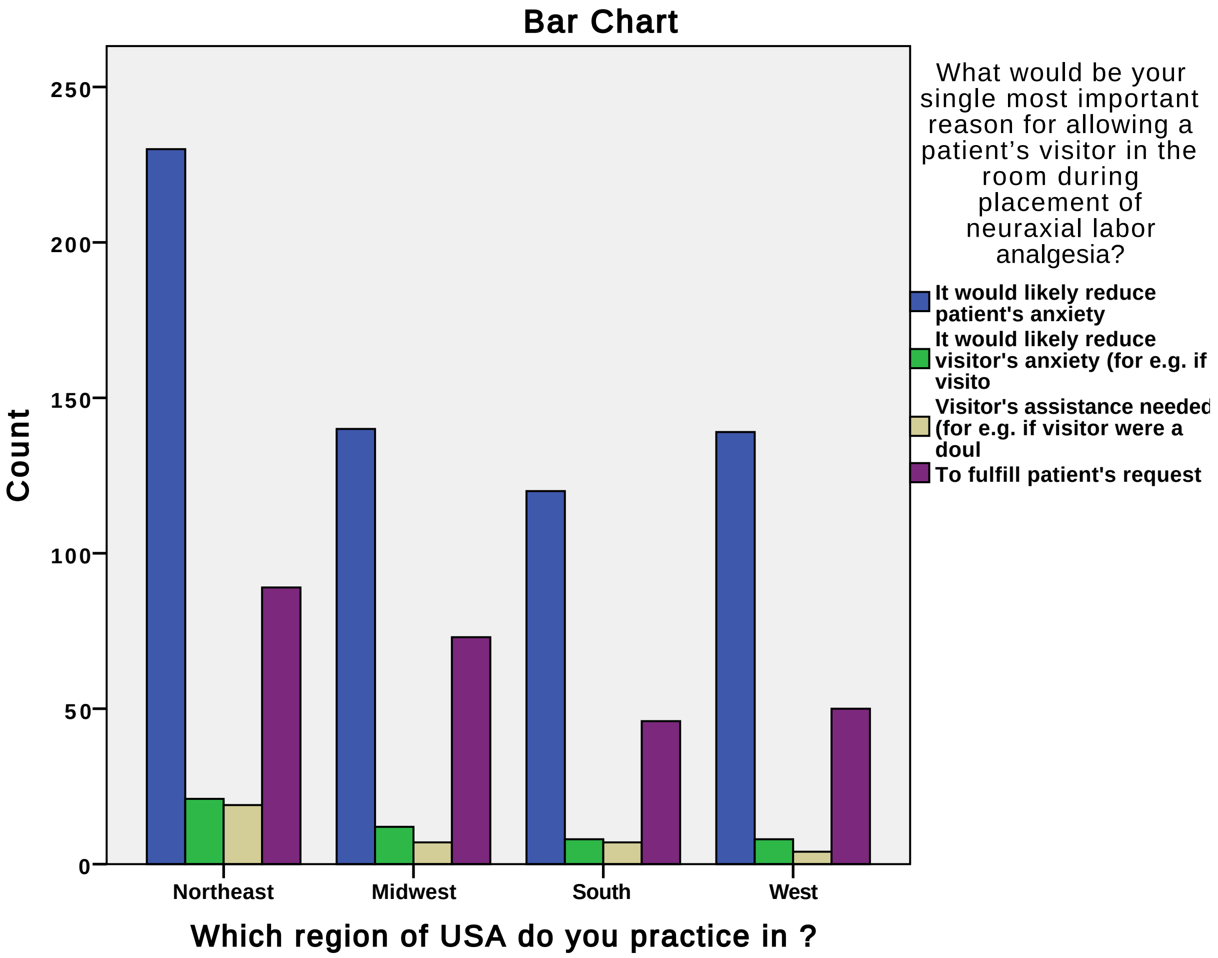

Which region of USA do you practice in ? * How often has another non anesthesia member of the labor and delivery team (for exa mple obstetrician or nurse) attempted to influence your decision to have a visitor present during placement of neuraxial labor ana lgesia?

Crosstab

			How often has another non anesthesia member of the labor and delivery team (for example obstetrician or nurse) attempted to influence your decision to have a visitor present during placement of neuraxial labor analgesia?		
			Rarely (< 5 %)	Occasionally (5 % - 40 %)	Often > 40 %
Which region of USA do you practice in ?	Northeast	Count	259	70	30
		% within Which region of USA do you practice in ?	72.1%	19.5%	8.4%
		Adjusted Residual	-.8	.9	.1
	Midwest	Count	173	43	16
		% within Which region of USA do you practice in ?	74.6%	18.5%	6.9%
		Adjusted Residual	.3	.2	-.8
	South	Count	135	29	17
		% within Which region of USA do you practice in ?	74.6%	16.0%	9.4%
		Adjusted Residual	.3	-.8	.6
	West	Count	150	34	17
		% within Which region of USA do you practice in ?	74.6%	16.9%	8.5%
		Adjusted Residual	.3	-.5	.1
Total		Count	717	176	80
		% within Which region of USA do you practice in ?	73.7%	18.1%	8.2%

Crosstab

			Total
Which region of USA do you practice in ?	Northeast	Count	359
		% within Which region of USA do you practice in ?	100.0%
		Adjusted Residual	
	Midwest	Count	232
		% within Which region of USA do you practice in ?	100.0%
		Adjusted Residual	
	South	Count	181
		% within Which region of USA do you practice in ?	100.0%
		Adjusted Residual	
	West	Count	201
		% within Which region of USA do you practice in ?	100.0%
		Adjusted Residual	
Total		Count	973
		% within Which region of USA do you practice in ?	100.0%

Chi-Square Tests

	Value	df	Asymp. Sig. (2-sided)
Pearson Chi-Square	2.003[a]	6	.919
Likelihood Ratio	2.030	6	.917
Linear-by-Linear Association	.138	1	.710
N of Valid Cases	973		

a. 0 cells (0.0%) have expected count less than 5. The minimum expected count is 14.88.

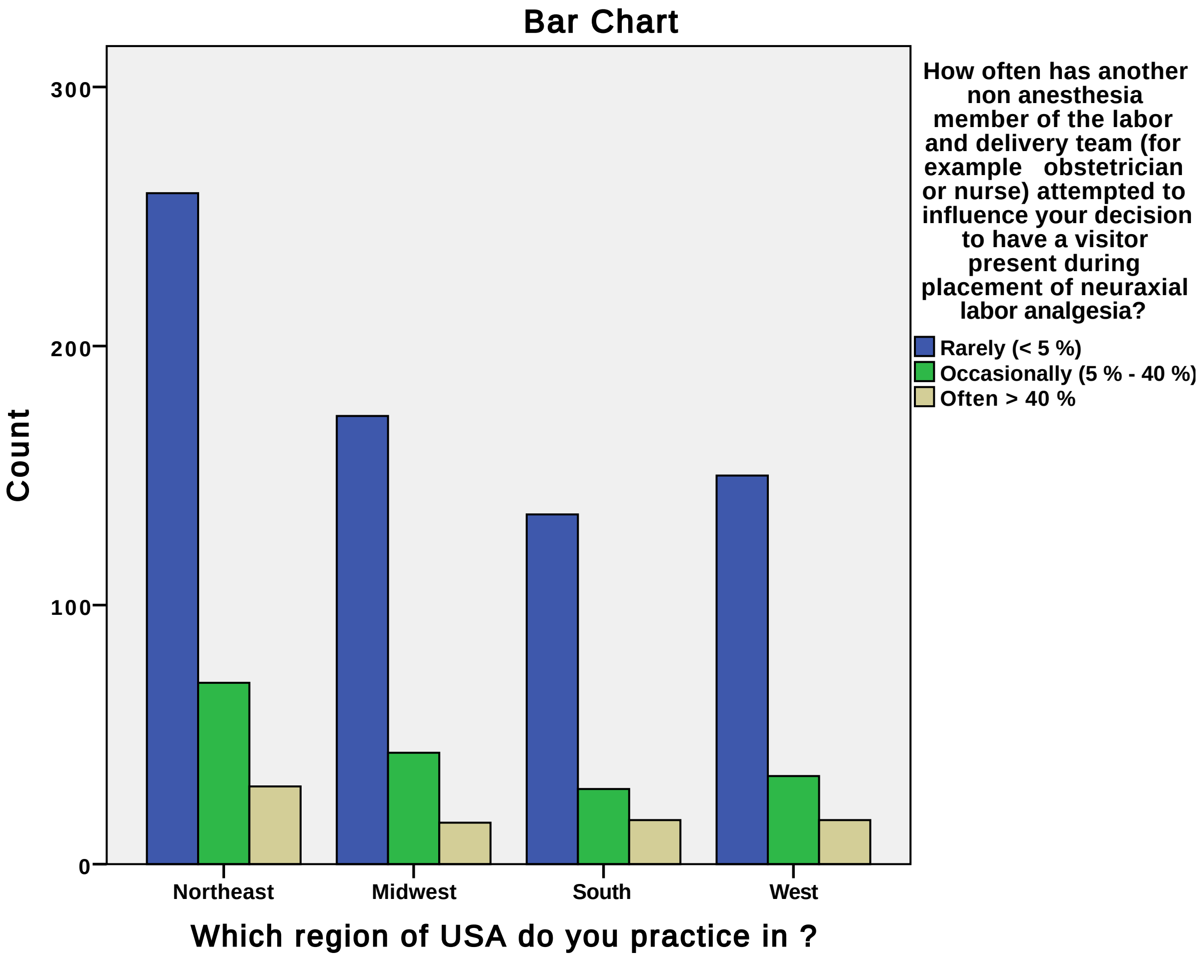

Which region of USA do you practice in ? * How often has another non anesthesia member of the labor and delivery team (for example obstetrician or nurse) attempted to influence your decision to NOT have a visitor present during placement of neuraxial labor analgesia?

Crosstab

			How often has another non anesthesia member of the labor and delivery team (for example obstetrician or nurse) attempted to influence your decision to NOT have a visitor present during placement of neuraxial labor analgesia?		
			Rarely < 5 %	Occasionally 5 - 40 %	Often > 40 %
Which region of USA do you practice in ?	Northeast	Count	146	27	9
		% within Which region of USA do you practice in ?	80.2%	14.8%	4.9%
		Adjusted Residual	.8	-.6	-.6
	Midwest	Count	38	10	3
		% within Which region of USA do you practice in ?	74.5%	19.6%	5.9%
		Adjusted Residual	-.8	.8	.1
	South	Count	48	9	4
		% within Which region of USA do you practice in ?	78.7%	14.8%	6.6%
		Adjusted Residual	.0	-.3	.4
	West	Count	21	5	2
		% within Which region of USA do you practice in ?	75.0%	17.9%	7.1%
		Adjusted Residual	-.5	.3	.4
Total		Count	253	51	18
		% within Which region of USA do you practice in ?	78.6%	15.8%	5.6%

Crosstab

			Total
Which region of USA do you practice in ?	Northeast	Count	182
		% within Which region of USA do you practice in ?	100.0%
		Adjusted Residual	
	Midwest	Count	51
		% within Which region of USA do you practice in ?	100.0%
		Adjusted Residual	
	South	Count	61
		% within Which region of USA do you practice in ?	100.0%
		Adjusted Residual	
	West	Count	28
		% within Which region of USA do you practice in ?	100.0%
		Adjusted Residual	
Total		Count	322
		% within Which region of USA do you practice in ?	100.0%

Chi-Square Tests

	Value	df	Asymp. Sig. (2-sided)
Pearson Chi-Square	1.272[a]	6	.973
Likelihood Ratio	1.233	6	.975
Linear-by-Linear Association	.540	1	.463
N of Valid Cases	322		

a. 4 cells (33.3%) have expected count less than 5. The minimum expected count is 1.57.

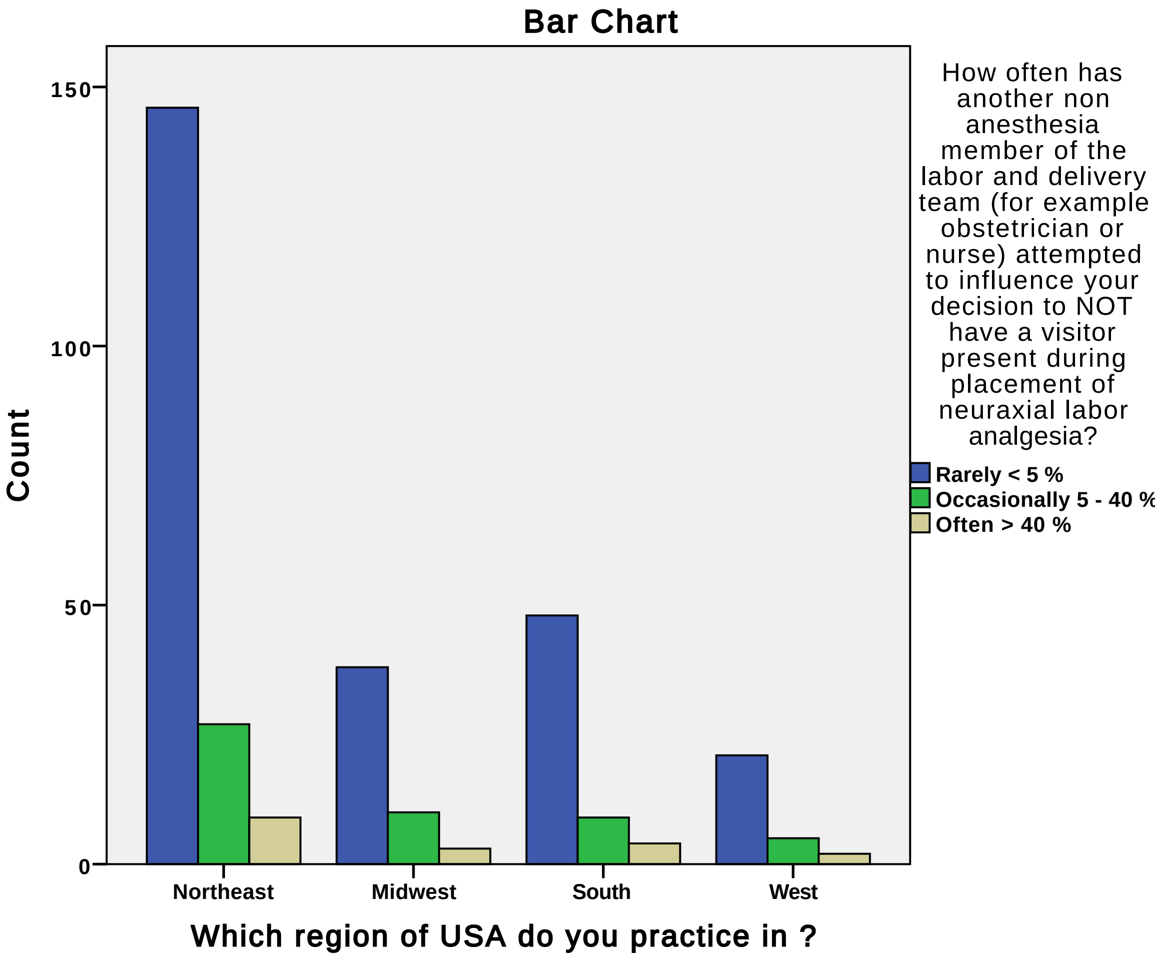

Which region of USA do you practice in ? * What would be your single most important reason for NOT allowing a patient's visitor in the room during placement of neuraxial labor analgesia?

Crosstab

			What would be your single most important reason for NOT allowing a patient's visitor in the room during placement of neuraxial labor ..		
			Increase in anesthesiologist's stress	Possible interference by visitor(for e.g. comments made if d	Concern about visitor (for e.g. passing out or not being ab
Which region of USA do you practice in ?	Northeast	Count	12	85	65
		% within Which region of USA do you practice in ?	6.6%	46.7%	35.7%
		Adjusted Residual	-.4	-.7	1.2
	Midwest	Count	4	26	16
		% within Which region of USA do you practice in ?	7.8%	51.0%	31.4%
		Adjusted Residual	.2	.4	-.3
	South	Count	2	32	17
		% within Which region of USA do you practice in ?	3.3%	52.5%	27.9%
		Adjusted Residual	-1.3	.7	-.9
	West	Count	5	13	8
		% within Which region of USA do you practice in ?	17.9%	46.4%	28.6%
		Adjusted Residual	2.3	-.2	-.5
Total		Count	23	156	106
		% within Which region of USA do you practice in ?	7.1%	48.4%	32.9%

Crosstab

			What would be your single most important ...	Total
			Medico-legal concerns	
Which region of USA do you practice in ?	Northeast	Count	20	182
		% within Which region of USA do you practice in ?	11.0%	100.0%
		Adjusted Residual	-.3	
	Midwest	Count	5	51
		% within Which region of USA do you practice in ?	9.8%	100.0%
		Adjusted Residual	-.4	
	South	Count	10	61
		% within Which region of USA do you practice in ?	16.4%	100.0%
		Adjusted Residual	1.3	
	West	Count	2	28
		% within Which region of USA do you practice in ?	7.1%	100.0%
		Adjusted Residual	-.8	
Total		Count	37	322
		% within Which region of USA do you practice in ?	11.5%	100.0%

Chi-Square Tests

	Value	df	Asymp. Sig. (2-sided)
Pearson Chi-Square	9.300[a]	9	.410
Likelihood Ratio	8.236	9	.511
Linear-by-Linear Association	.710	1	.400
N of Valid Cases	322		

a. 4 cells (25.0%) have expected count less than 5. The minimum expected count is 2.00.

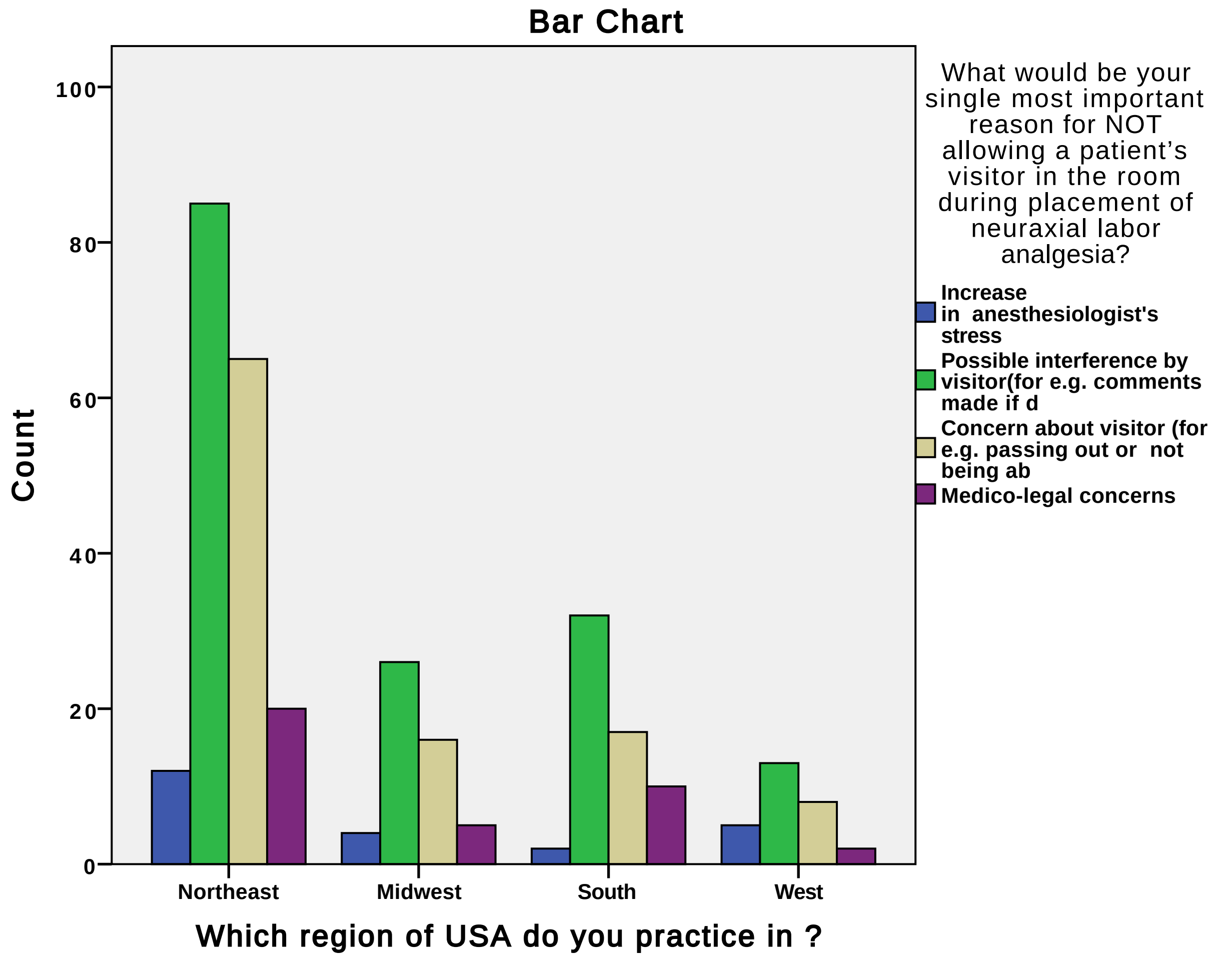

What is the setting of your practice ? * What is your preferred tec hnique for neuraxial labor analgesia in an otherwise healthy part urient?

Crosstab

			What is your preferred technique for neuraxial labor analgesia in an otherwise healthy parturient?		
			Epidural analgesia	Combined spinal epidural analgesia	Total
What is the setting of your practice ?	Rural	Count	59	24	83
		% within What is the setting of your practice ?	71.1%	28.9%	100.0%
		Adjusted Residual	-1.6	1.6	
	Suburban	Count	359	91	450
		% within What is the setting of your practice ?	79.8%	20.2%	100.0%
		Adjusted Residual	1.1	-1.1	
	Urban	Count	597	171	768
		% within What is the setting of your practice ?	77.7%	22.3%	100.0%
		Adjusted Residual	-.3	.3	
Total		Count	1015	286	1301
		% within What is the setting of your practice ?	78.0%	22.0%	100.0%

Chi-Square Tests

	Value	df	Asymp. Sig. (2-sided)
Pearson Chi-Square	3.175[a]	2	.204
Likelihood Ratio	3.042	2	.219
Linear-by-Linear Association	.153	1	.696
N of Valid Cases	1301		

a. 0 cells (0.0%) have expected count less than 5. The minimum expected count is 18.25.

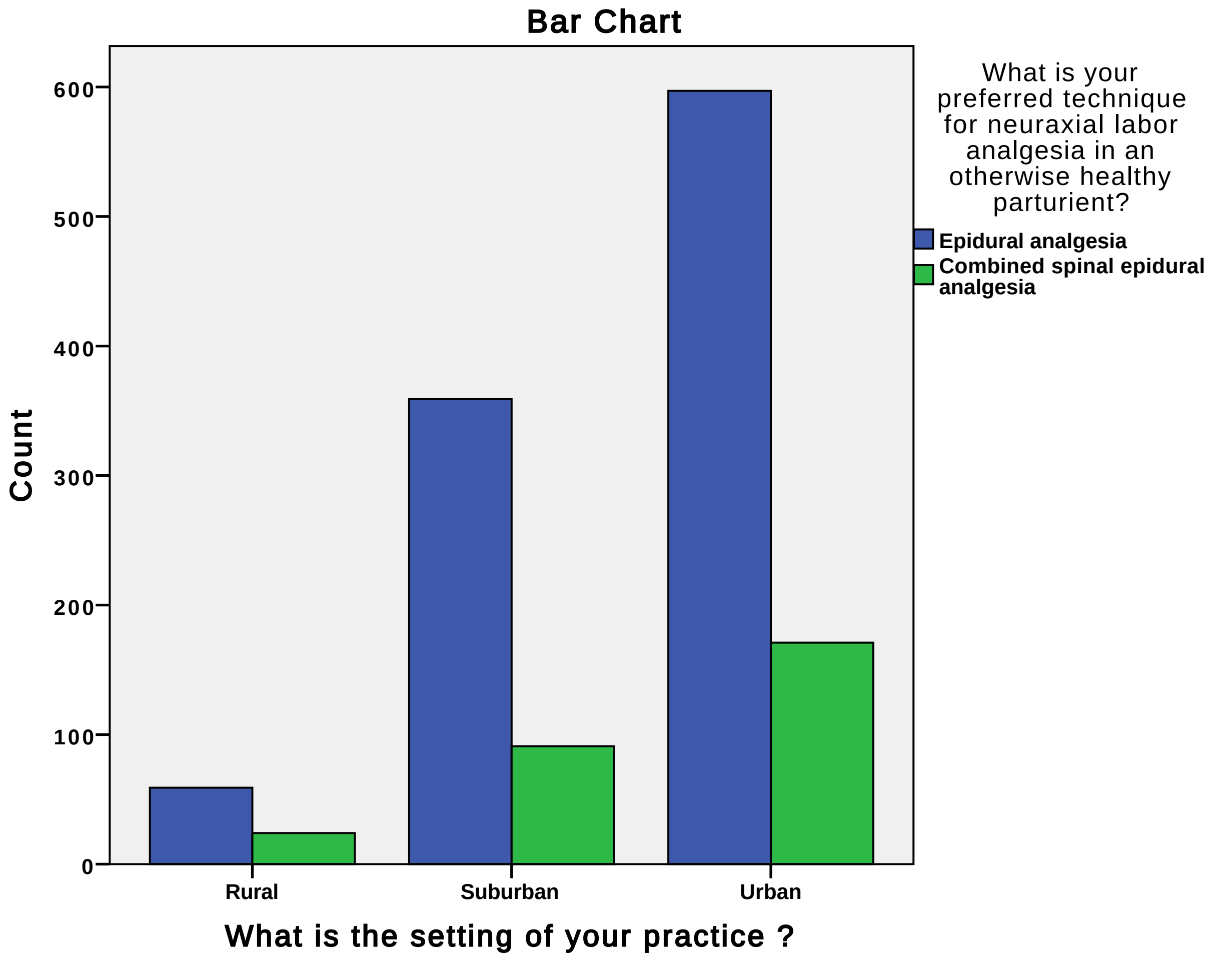

What is the setting of your practice ? * Does your practice or hospital have a written policy regarding allowing a patient's visitor in the room during placement of neuraxial labor analgesia?

Crosstab

			Does your practice or hospital have a written policy regarding allowing a patient's visitor in the room during placement of neuraxial labor ..		
			Yes, a written policy exists - Allows visitors	Yes, a written policy exists - Does not allow visitors	No written policy exists
What is the setting of your practice ?	Rural	Count	14	3	49
		% within What is the setting of your practice ?	16.9%	3.6%	59.0%
		Adjusted Residual	-1.3	-1.5	3.8
	Suburban	Count	91	32	193
		% within What is the setting of your practice ?	20.2%	7.1%	42.9%
		Adjusted Residual	-1.4	-.9	1.9
	Urban	Count	188	70	271
		% within What is the setting of your practice ?	24.5%	9.1%	35.3%
		Adjusted Residual	2.0	1.7	-3.7
Total		Count	293	105	513
		% within What is the setting of your practice ?	22.5%	8.1%	39.4%

Crosstab

			Does your practice or hospital have a ...	
			I do not know	Total
What is the setting of your practice ?	Rural	Count	17	83
		% within What is the setting of your practice ?	20.5%	100.0%
		Adjusted Residual	-2.0	
	Suburban	Count	134	450
		% within What is the setting of your practice ?	29.8%	100.0%
		Adjusted Residual	-.1	
	Urban	Count	239	768
		% within What is the setting of your practice ?	31.1%	100.0%
		Adjusted Residual	1.1	
Total		Count	390	1301
		% within What is the setting of your practice ?	30.0%	100.0%

Chi-Square Tests

	Value	df	Asymp. Sig. (2-sided)
Pearson Chi-Square	22.772[a]	6	.001
Likelihood Ratio	22.716	6	.001
Linear-by-Linear Association	1.978	1	.160
N of Valid Cases	1301		

a. 0 cells (0.0%) have expected count less than 5. The minimum expected count is 6.70.

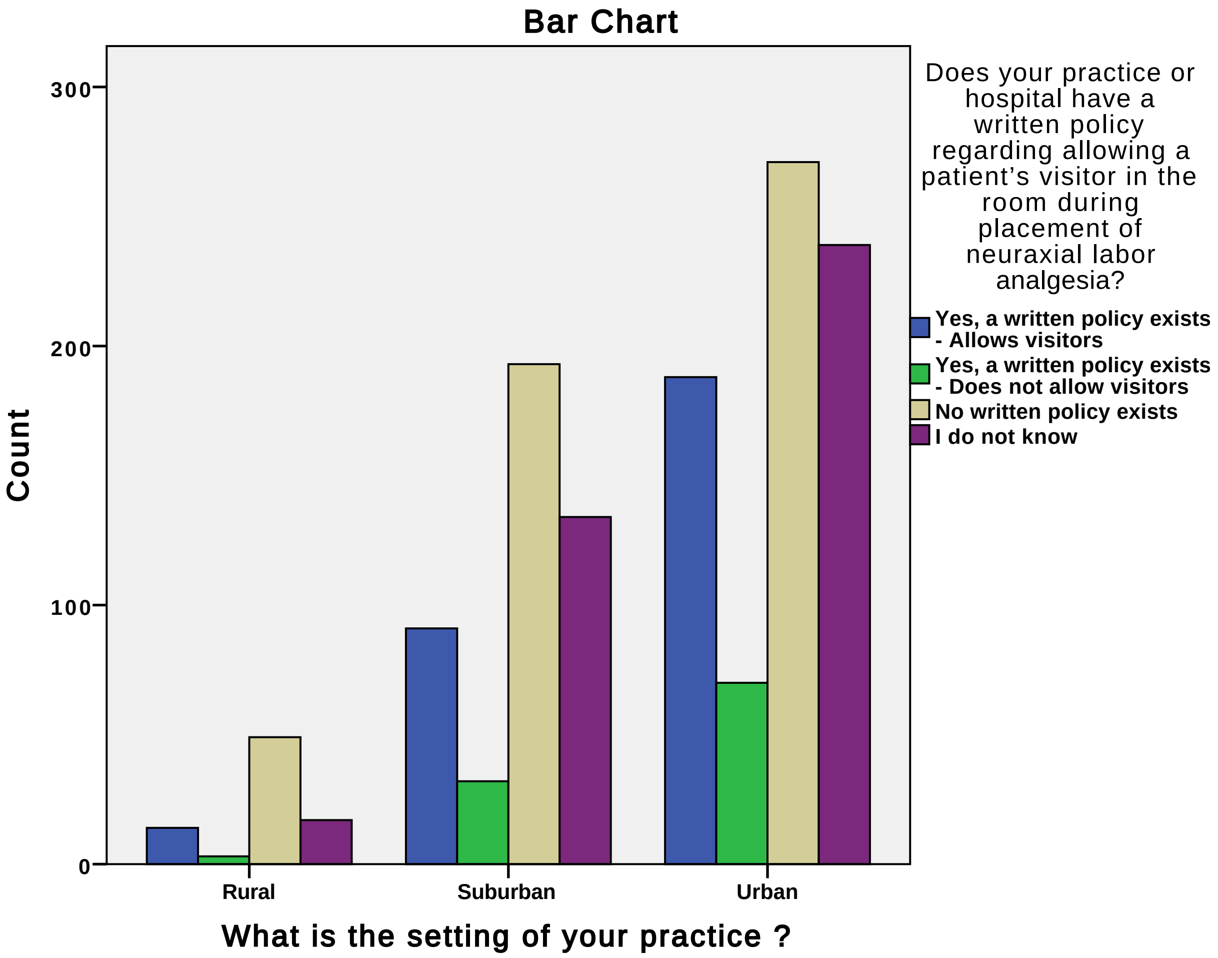

What is the setting of your practice ? * If no policy existed, would you be open to allowing a patient’s visitor in the room during pla cement of neuraxial labor analgesia?

Crosstab

			If no policy existed, would you be open to allowing a patient's visitor in the room during placement of neuraxial labor analgesia?		Total
			Yes	No	
What is the setting of your practice ?	Rural	Count	72	11	83
		% within What is the setting of your practice ?	86.7%	13.3%	100.0%
		Adjusted Residual	2.5	-2.5	
	Suburban	Count	340	110	450
		% within What is the setting of your practice ?	75.6%	24.4%	100.0%
		Adjusted Residual	.3	-.3	
	Urban	Count	565	203	768
		% within What is the setting of your practice ?	73.6%	26.4%	100.0%
		Adjusted Residual	-1.5	1.5	
Total		Count	977	324	1301
		% within What is the setting of your practice ?	75.1%	24.9%	100.0%

Chi-Square Tests

	Value	df	Asymp. Sig. (2-sided)
Pearson Chi-Square	7.034[a]	2	.030
Likelihood Ratio	7.889	2	.019
Linear-by-Linear Association	4.994	1	.025
N of Valid Cases	1301		

a. 0 cells (0.0%) have expected count less than 5. The minimum expected count is 20.67.

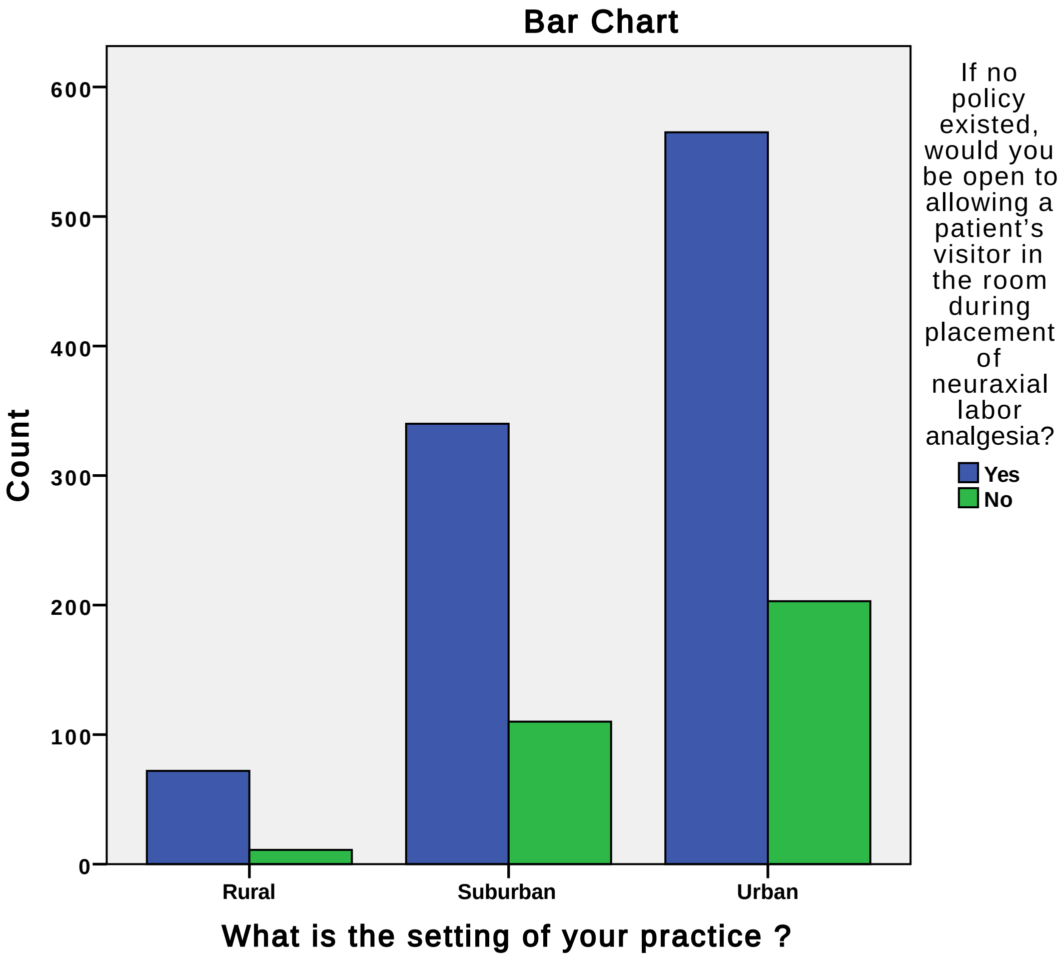

What is the setting of your practice ? * If you allowed a visitor in the room during placement of neuraxial labor analgesia, you would want the

Crosstab

			If you allowed a visitor in the room during placement of neuraxial labor analgesia, you would want the		
			Visitor always standing	Visitor always sitting	Does not matter
What is the setting of your practice ?	Rural	Count	2	30	40
		% within What is the setting of your practice ?	2.8%	41.7%	55.6%
		Adjusted Residual	1.0	-6.0	5.8
	Suburban	Count	4	250	86
		% within What is the setting of your practice ?	1.2%	73.5%	25.3%
		Adjusted Residual	-.5	.8	-.6
	Urban	Count	8	421	132
		% within What is the setting of your practice ?	1.4%	75.0%	23.5%
		Adjusted Residual	.0	2.4	-2.5
Total		Count	14	701	258
		% within What is the setting of your practice ?	1.4%	72.0%	26.5%

Crosstab

			Total
What is the setting of your practice ?	Rural	Count	72
		% within What is the setting of your practice ?	100.0%
		Adjusted Residual	
	Suburban	Count	340
		% within What is the setting of your practice ?	100.0%
		Adjusted Residual	
	Urban	Count	561
		% within What is the setting of your practice ?	100.0%
		Adjusted Residual	
Total		Count	973
		% within What is the setting of your practice ?	100.0%

Chi-Square Tests

	Value	df	Asymp. Sig. (2-sided)
Pearson Chi-Square	36.065[a]	4	.000
Likelihood Ratio	32.072	4	.000
Linear-by-Linear Association	15.973	1	.000
N of Valid Cases	973		

a. 2 cells (22.2%) have expected count less than 5. The minimum expected count is 1.04.

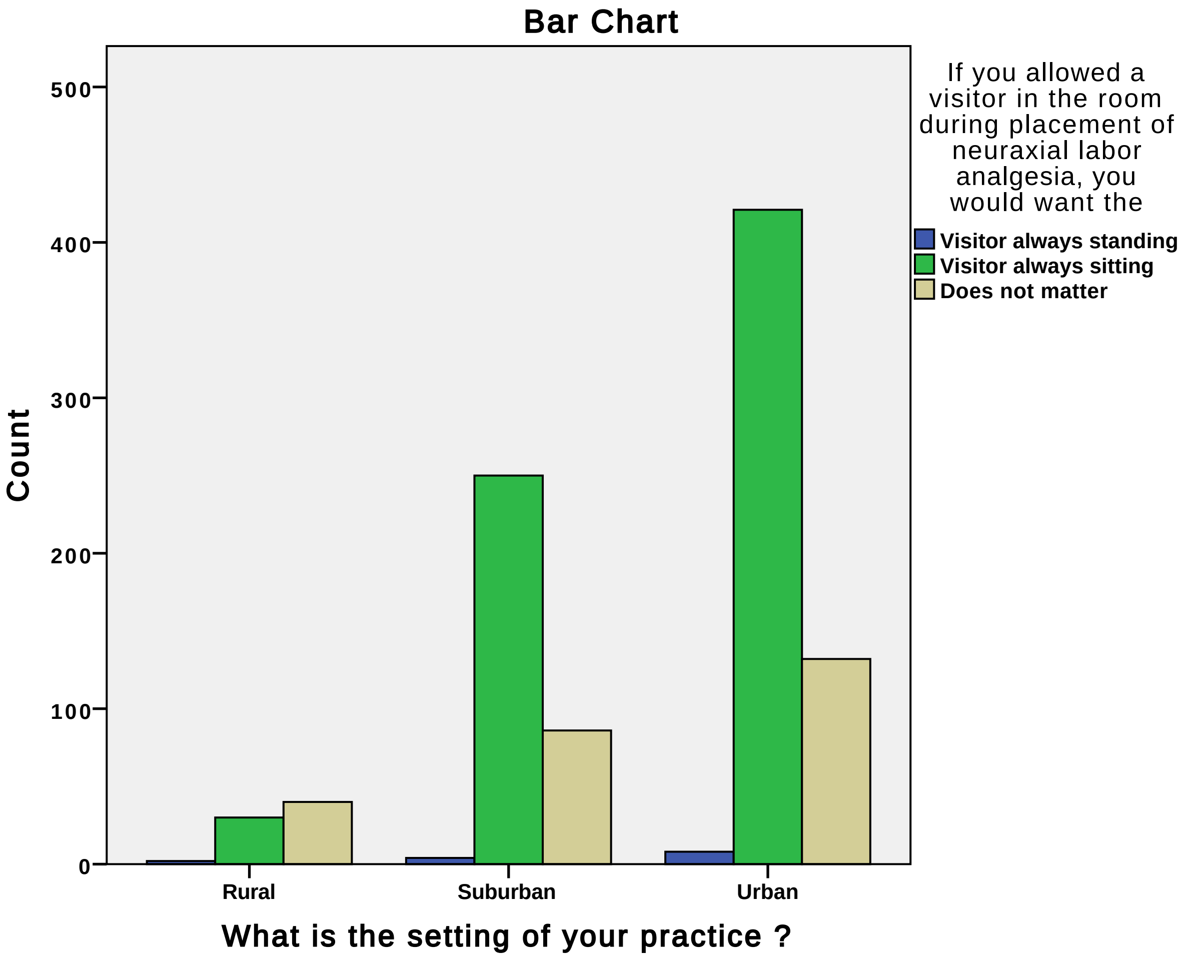

What is the setting of your practice ? * If you allowed a visitor in the room during placement of neuraxial labor analgesia, you would want the visitor to be positioned such that,

Crosstab

			If you allowed a visitor in the room during placement of neuraxial labor analgesia, you would want the visitor to be positioned such that,		
			Visitor has no view of procedure (patient is in between anes	Visitor has partial view of workspace (but cannot see patien	Does not matter
What is the setting of your practice ?	Rural	Count	36	10	26
		% within What is the setting of your practice ?	50.0%	13.9%	36.1%
		Adjusted Residual	-2.0	-1.2	3.6
	Suburban	Count	213	69	58
		% within What is the setting of your practice ?	62.6%	20.3%	17.1%
		Adjusted Residual	.7	.7	-1.6
	Urban	Count	345	106	110
		% within What is the setting of your practice ?	61.5%	18.9%	19.6%
		Adjusted Residual	.3	-.1	-.3
Total		Count	594	185	194
		% within What is the setting of your practice ?	61.0%	19.0%	19.9%

Crosstab

			Total
What is the setting of your practice ?	Rural	Count	72
		% within What is the setting of your practice ?	100.0%
		Adjusted Residual	
	Suburban	Count	340
		% within What is the setting of your practice ?	100.0%
		Adjusted Residual	
	Urban	Count	561
		% within What is the setting of your practice ?	100.0%
		Adjusted Residual	
Total		Count	973
		% within What is the setting of your practice ?	100.0%

Chi-Square Tests

	Value	df	Asymp. Sig. (2-sided)
Pearson Chi-Square	13.782[a]	4	.008
Likelihood Ratio	12.199	4	.016
Linear-by-Linear Association	2.313	1	.128
N of Valid Cases	973		

a. 0 cells (0.0%) have expected count less than 5. The minimum expected count is 13.69.

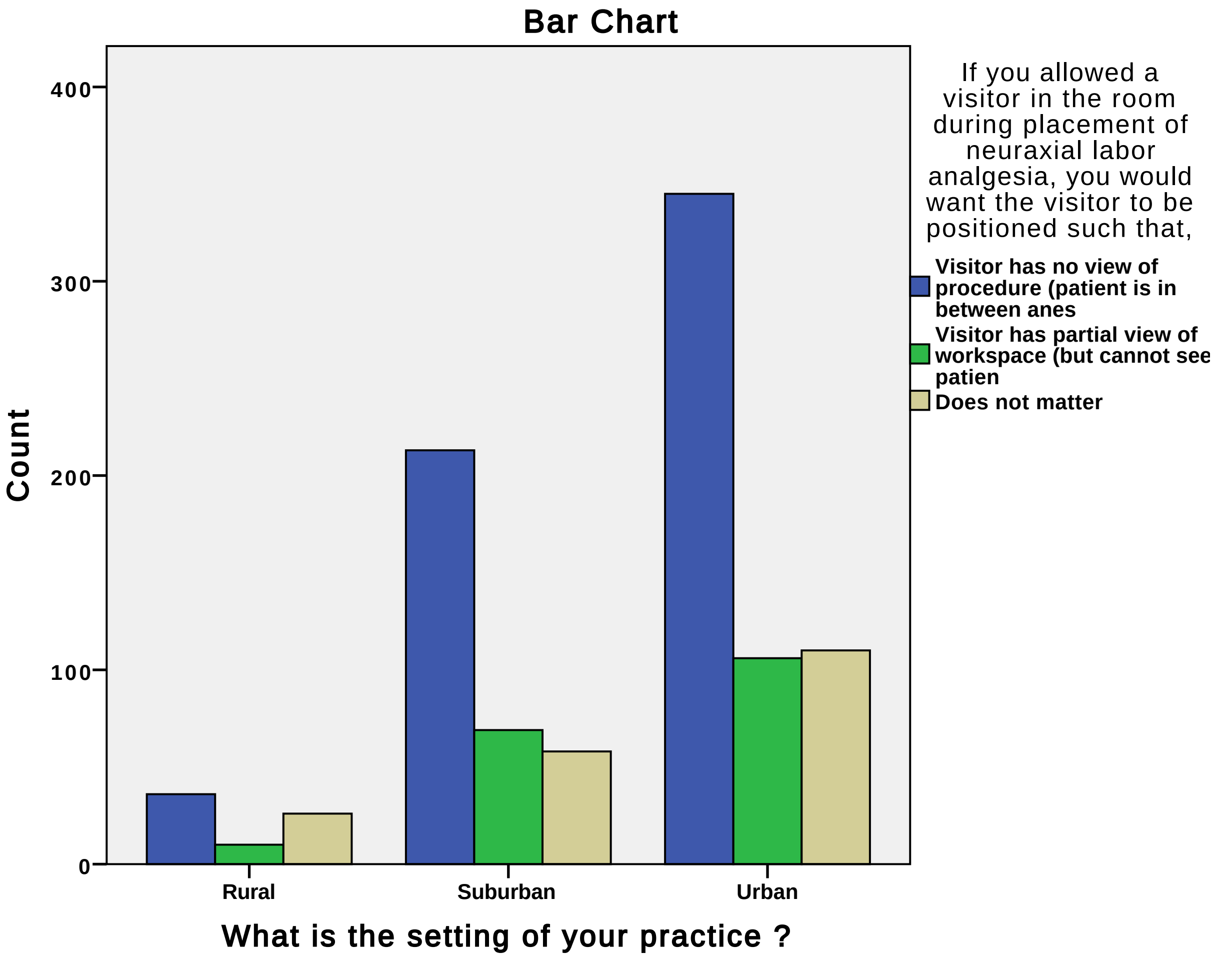

What is the setting of your practice ? * What would be your single most important reason for allowing a patient’s visitor in the room during placement of neuraxial labor analgesia?

Crosstab

			What would be your single most important reason for allowing a patient’s visitor in the room during placement of neuraxial labor ...		
			It would likely reduce patient's anxiety	It would likely reduce visitor's anxiety (for e. g. if visito	Visitor's assistance needed (for e. g. if visitor were a doul
What is the setting of your practice ?	Rural	Count	48	3	3
		% within What is the setting of your practice ?	66.7%	4.2%	4.2%
		Adjusted Residual	.4	-.4	.2
	Suburban	Count	222	16	13
		% within What is the setting of your practice ?	65.3%	4.7%	3.8%
		Adjusted Residual	.3	-.3	.0
	Urban	Count	359	30	21
		% within What is the setting of your practice ?	64.0%	5.3%	3.7%
		Adjusted Residual	-.5	.5	-.1
Total		Count	629	49	37
		% within What is the setting of your practice ?	64.6%	5.0%	3.8%

Crosstab

			What would be your single most important ...	
			To fulfill patient's request	Total
What is the setting of your practice ?	Rural	Count	18	72
		% within What is the setting of your practice ?	25.0%	100.0%
		Adjusted Residual	-.3	
	Suburban	Count	89	340
		% within What is the setting of your practice ?	26.2%	100.0%
		Adjusted Residual	-.2	
	Urban	Count	151	561
		% within What is the setting of your practice ?	26.9%	100.0%
		Adjusted Residual	.3	
Total		Count	258	973
		% within What is the setting of your practice ?	26.5%	100.0%

Chi-Square Tests

	Value	df	Asymp. Sig. (2-sided)
Pearson Chi-Square	.536[a]	6	.997
Likelihood Ratio	.542	6	.997
Linear-by-Linear Association	.186	1	.666
N of Valid Cases	973		

a. 2 cells (16.7%) have expected count less than 5. The minimum expected count is 2.74.

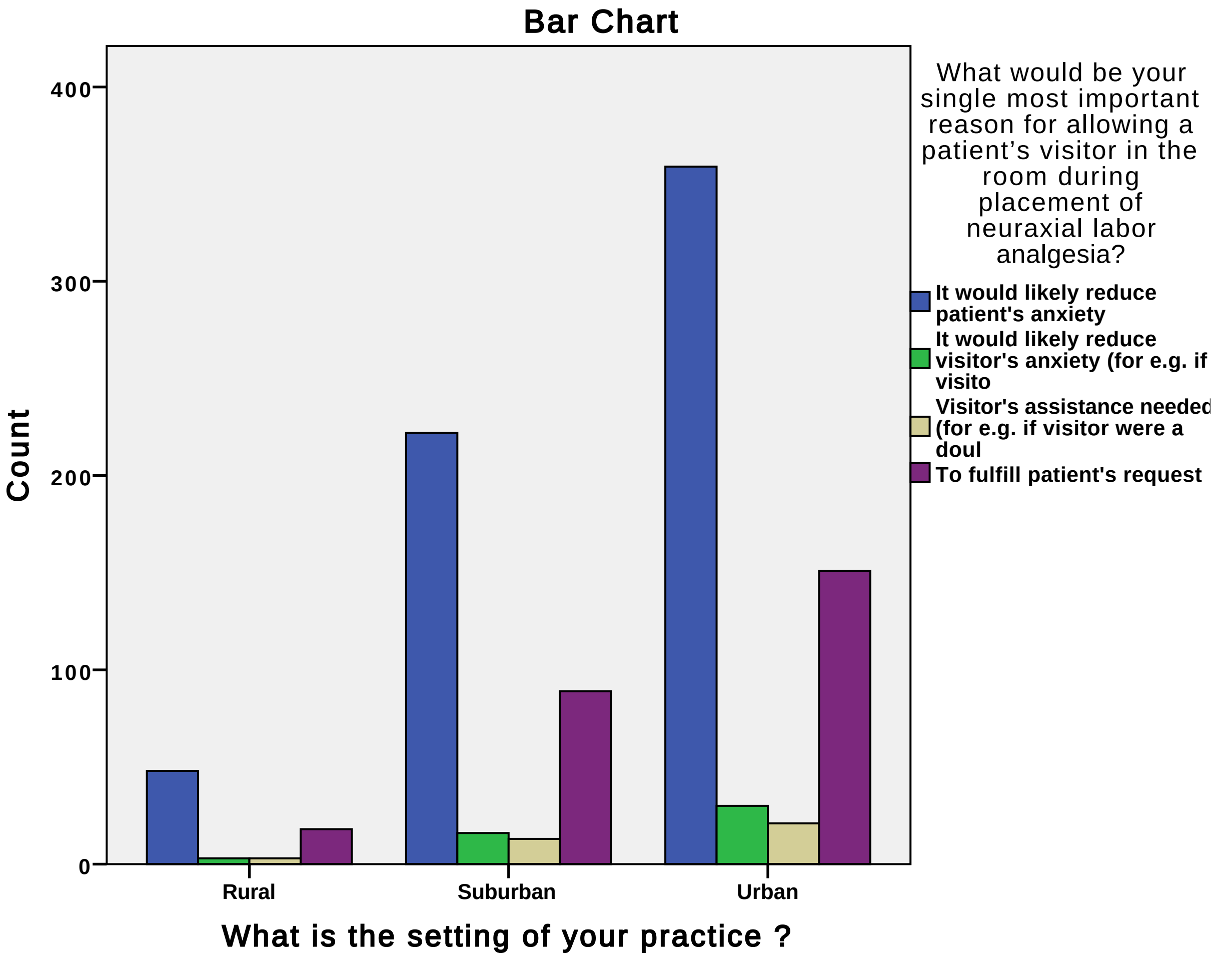

What is the setting of your practice ? * How often has another non anesthesia member of the labor and delivery team (for example obstetrician or nurse) attempted to influence your decision to have a visitor present during placement of neuraxial labor analgesia?

Crosstab

			How often has another non anesthesia member of the labor and delivery team (for example obstetrician or nurse) attempted to influence your decision to have a visitor present during placement of neuraxial labor analgesia?		
			Rarely (< 5 %)	Occasionally (5 % - 40 %)	Often > 40 %
What is the setting of your practice ?	Rural	Count	50	15	7
		% within What is the setting of your practice ?	69.4%	20.8%	9.7%
		Adjusted Residual	-.9	.6	.5
	Suburban	Count	235	69	36
		% within What is the setting of your practice ?	69.1%	20.3%	10.6%
		Adjusted Residual	-2.4	1.3	2.0
	Urban	Count	432	92	37
		% within What is the setting of your practice ?	77.0%	16.4%	6.6%
		Adjusted Residual	2.7	-1.6	-2.2
Total		Count	717	176	80
		% within What is the setting of your practice ?	73.7%	18.1%	8.2%

Crosstab

			Total
What is the setting of your practice ?	Rural	Count	72
		% within What is the setting of your practice ?	100.0%
		Adjusted Residual	
	Suburban	Count	340
		% within What is the setting of your practice ?	100.0%
		Adjusted Residual	
	Urban	Count	561
		% within What is the setting of your practice ?	100.0%
		Adjusted Residual	
Total		Count	973
		% within What is the setting of your practice ?	100.0%

Chi-Square Tests

	Value	df	Asymp. Sig. (2-sided)
Pearson Chi-Square	8.395[a]	4	.078
Likelihood Ratio	8.313	4	.081
Linear-by-Linear Association	6.707	1	.010
N of Valid Cases	973		

a. 0 cells (0.0%) have expected count less than 5. The minimum expected count is 5.92.

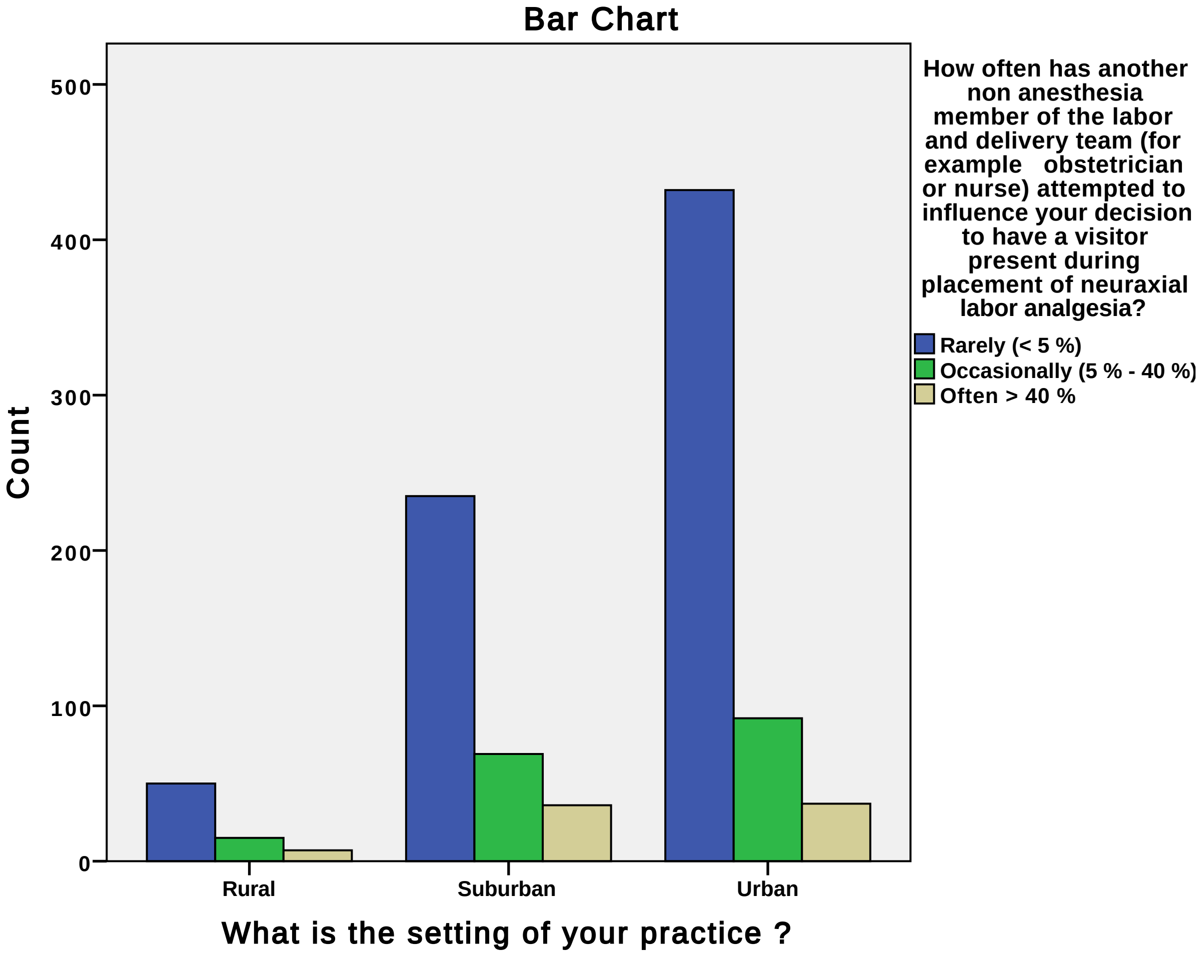

What is the setting of your practice ? * How often has another non anesthesia member of the labor and delivery team (for example obstetrician or nurse) attempted to influence your decision to NO T have a visitor present during placement of neuraxial labor anal gesia?

Crosstab

			How often has another non anesthesia member of the labor and delivery team (for example obstetrician or nurse) attempted to influence your decision to NOT have a visitor present during placement of neuraxial labor analgesia?		
			Rarely < 5 %	Occasionally 5 - 40 %	Often > 40 %
What is the setting of your practice ?	Rural	Count	7	3	1
		% within What is the setting of your practice ?	63.6%	27.3%	9.1%
		Adjusted Residual	-1.2	1.1	.5
	Suburban	Count	85	15	8
		% within What is the setting of your practice ?	78.7%	13.9%	7.4%
		Adjusted Residual	.0	-.7	1.0
	Urban	Count	161	33	9
		% within What is the setting of your practice ?	79.3%	16.3%	4.4%
		Adjusted Residual	.4	.3	-1.2
Total		Count	253	51	18
		% within What is the setting of your practice ?	78.6%	15.8%	5.6%

Crosstab

			Total
What is the setting of your practice ?	Rural	Count	11
		% within What is the setting of your practice ?	100.0%
		Adjusted Residual	
	Suburban	Count	108
		% within What is the setting of your practice ?	100.0%
		Adjusted Residual	
	Urban	Count	203
		% within What is the setting of your practice ?	100.0%
		Adjusted Residual	
Total		Count	322
		% within What is the setting of your practice ?	100.0%

Chi-Square Tests

	Value	df	Asymp. Sig. (2-sided)
Pearson Chi-Square	2.881[a]	4	.578
Likelihood Ratio	2.690	4	.611
Linear-by-Linear Association	1.119	1	.290
N of Valid Cases	322		

a. 2 cells (22.2%) have expected count less than 5. The minimum expected count is .61.

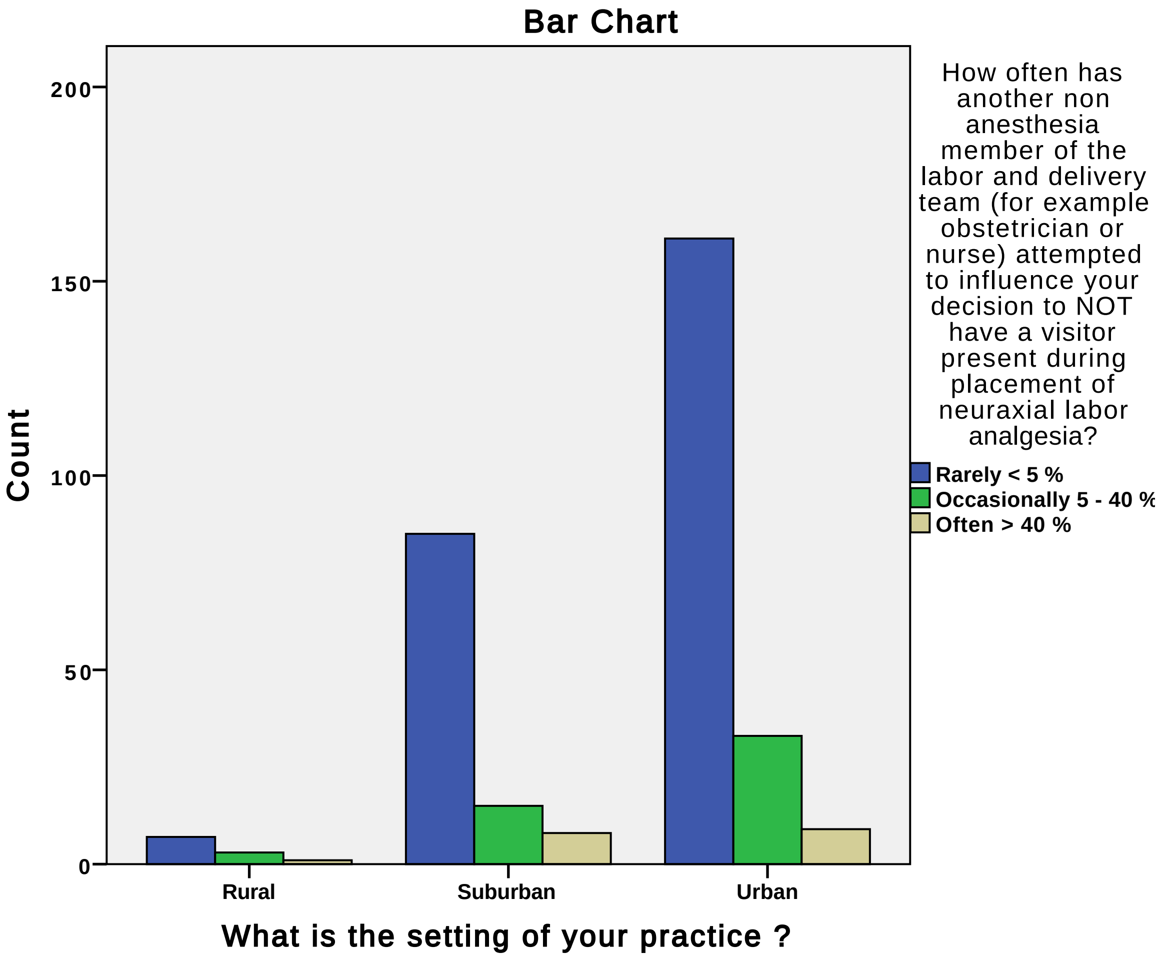

What is the setting of your practice ? * What would be your single most important reason for NOT allowing a patient's visitor in the room during placement of neuraxial labor analgesia?

Crosstab

			What would be your single most important reason for NOT allowing a patient's visitor in the room during placement of neuraxial labor ..		
			Increase in anesthesiologist's stress	Possible interference by visitor(for e.g. comments made if d	Concern about visitor (for e.g. passing out or nc being ab
What is the setting of your practice ?	Rural	Count	3	3	2
		% within What is the setting of your practice ?	27.3%	27.3%	18.2%
		Adjusted Residual	2.6	-1.4	-1.1
	Suburban	Count	8	56	32
		% within What is the setting of your practice ?	7.4%	51.9%	29.6%
		Adjusted Residual	.1	.9	-.9
	Urban	Count	12	97	72
		% within What is the setting of your practice ?	5.9%	47.8%	35.5%
		Adjusted Residual	-1.1	-.3	1.3
Total		Count	23	156	106
		% within What is the setting of your practice ?	7.1%	48.4%	32.9%

Crosstab

			What would be your single most important ...	
			Medico-legal concerns	Total
What is the setting of your practice ?	Rural	Count	3	11
		% within What is the setting of your practice ?	27.3%	100.0%
		Adjusted Residual	1.7	
	Suburban	Count	12	108
		% within What is the setting of your practice ?	11.1%	100.0%
		Adjusted Residual	-.2	
	Urban	Count	22	203
		% within What is the setting of your practice ?	10.8%	100.0%
		Adjusted Residual	-.5	
Total		Count	37	322
		% within What is the setting of your practice ?	11.5%	100.0%

Chi-Square Tests

	Value	df	Asymp. Sig. (2-sided)
Pearson Chi-Square	11.931[a]	6	.064
Likelihood Ratio	8.991	6	.174
Linear-by-Linear Association	.467	1	.495
N of Valid Cases	322		

a. 3 cells (25.0%) have expected count less than 5. The minimum expected count is .79.

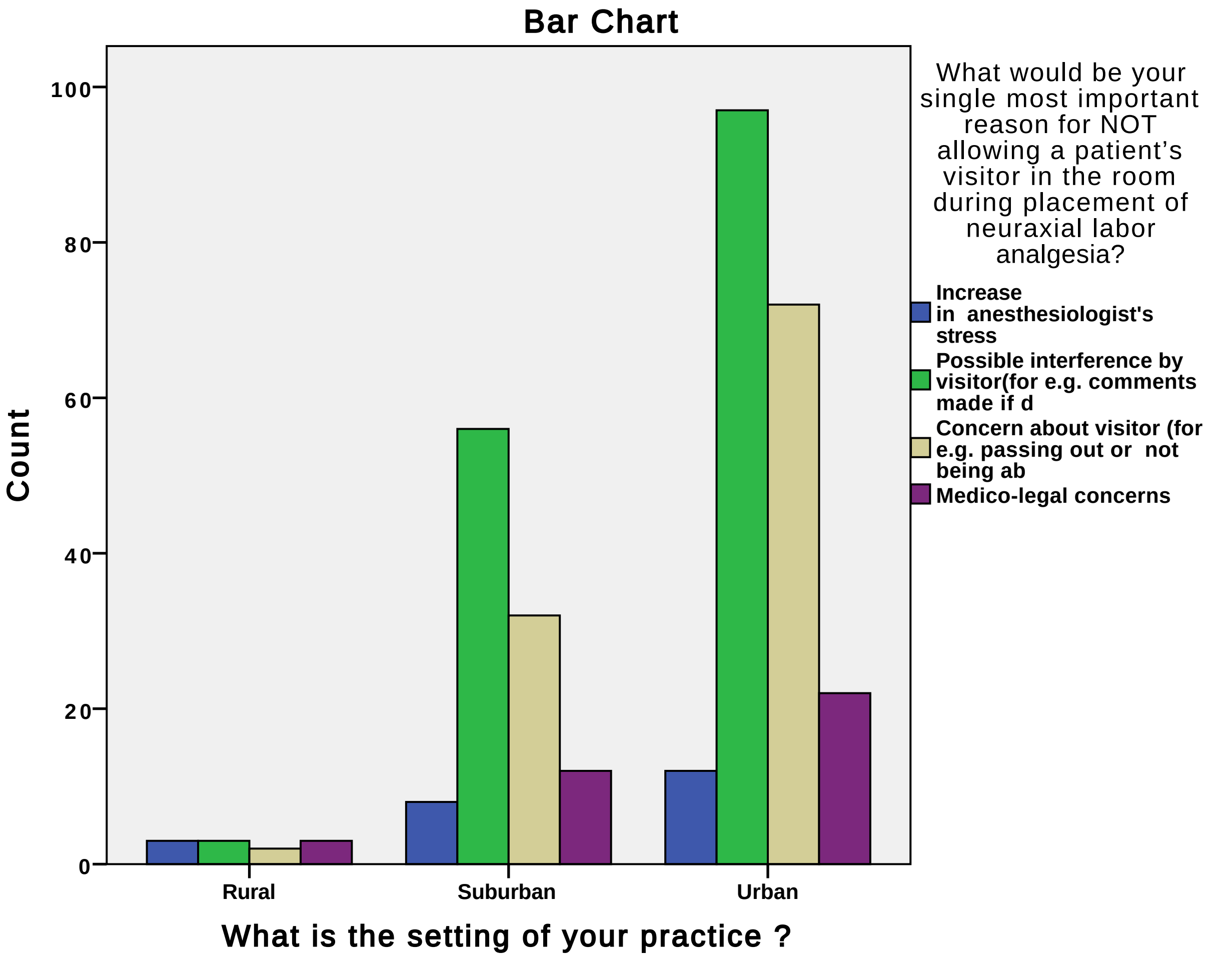

Describe the nature of your practice ? * What is your preferred technique for neuraxial labor analgesia in an otherwise healthy parturient?

Crosstab

			What is your preferred technique for neuraxial labor analgesia in an otherwise healthy parturient?		
			Epidural analgesia	Combined spinal epidural analgesia	Total
Describe the nature of your practice ?	Academic	Count	420	161	581
		% within Describe the nature of your practice ?	72.3%	27.7%	100.0%
		Adjusted Residual	-4.5	4.5	
	Private	Count	561	121	682
		% within Describe the nature of your practice ?	82.3%	17.7%	100.0%
		Adjusted Residual	3.9	-3.9	
	Government	Count	34	4	38
		% within Describe the nature of your practice ?	89.5%	10.5%	100.0%
		Adjusted Residual	1.7	-1.7	
Total		Count	1015	286	1301
		% within Describe the nature of your practice ?	78.0%	22.0%	100.0%

Chi-Square Tests

	Value	df	Asymp. Sig. (2-sided)
Pearson Chi-Square	21.175[a]	2	.000
Likelihood Ratio	21.457	2	.000
Linear-by-Linear Association	21.028	1	.000
N of Valid Cases	1301		

a. 0 cells (0.0%) have expected count less than 5. The minimum expected count is 8.35.

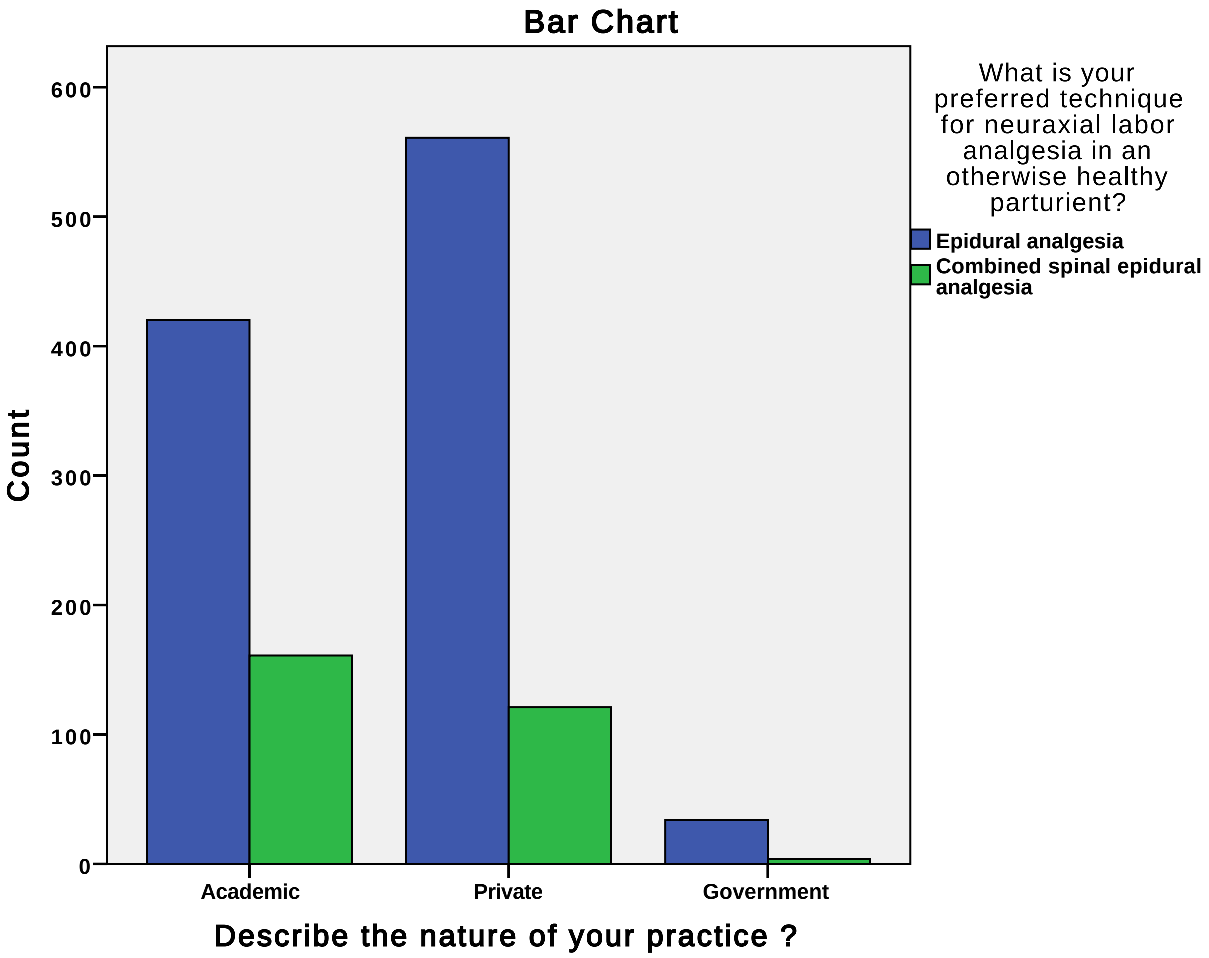

Describe the nature of your practice ? * Does your practice or hospital have a written policy regarding allowing a patient's visitor in the room during placement of neuraxial labor analgesia?

Crosstab

			Does your practice or hospital have a written policy regarding allowing a patient's visitor in the room during placement of neuraxial labor ..		
			Yes, a written policy exists - Allows visitors	Yes, a written policy exists - Does not allow visitors	No written policy exists
Describe the nature of your practice ?	Academic	Count	144	49	184
		% within Describe the nature of your practice ?	24.8%	8.4%	31.7%
		Adjusted Residual	1.8	.4	-5.1
	Private	Count	144	55	308
		% within Describe the nature of your practice ?	21.1%	8.1%	45.2%
		Adjusted Residual	-1.3	.0	4.4
	Government	Count	5	1	21
		% within Describe the nature of your practice ?	13.2%	2.6%	55.3%
		Adjusted Residual	-1.4	-1.2	2.0
Total		Count	293	105	513
		% within Describe the nature of your practice ?	22.5%	8.1%	39.4%

Crosstab

			Does your practice or hospital have a ...	
			I do not know	Total
Describe the nature of your practice ?	Academic	Count	204	581
		% within Describe the nature of your practice ?	35.1%	100.0%
		Adjusted Residual	3.6	
	Private	Count	175	682
		% within Describe the nature of your practice ?	25.7%	100.0%
		Adjusted Residual	-3.6	
	Government	Count	11	38
		% within Describe the nature of your practice ?	28.9%	100.0%
		Adjusted Residual	-.1	
Total		Count	390	1301
		% within Describe the nature of your practice ?	30.0%	100.0%

Chi-Square Tests

	Value	df	Asymp. Sig. (2-sided)
Pearson Chi-Square	31.224[a]	6	.000
Likelihood Ratio	32.026	6	.000
Linear-by-Linear Association	.113	1	.737
N of Valid Cases	1301		

a. 1 cells (8.3%) have expected count less than 5. The minimum expected count is 3.07.

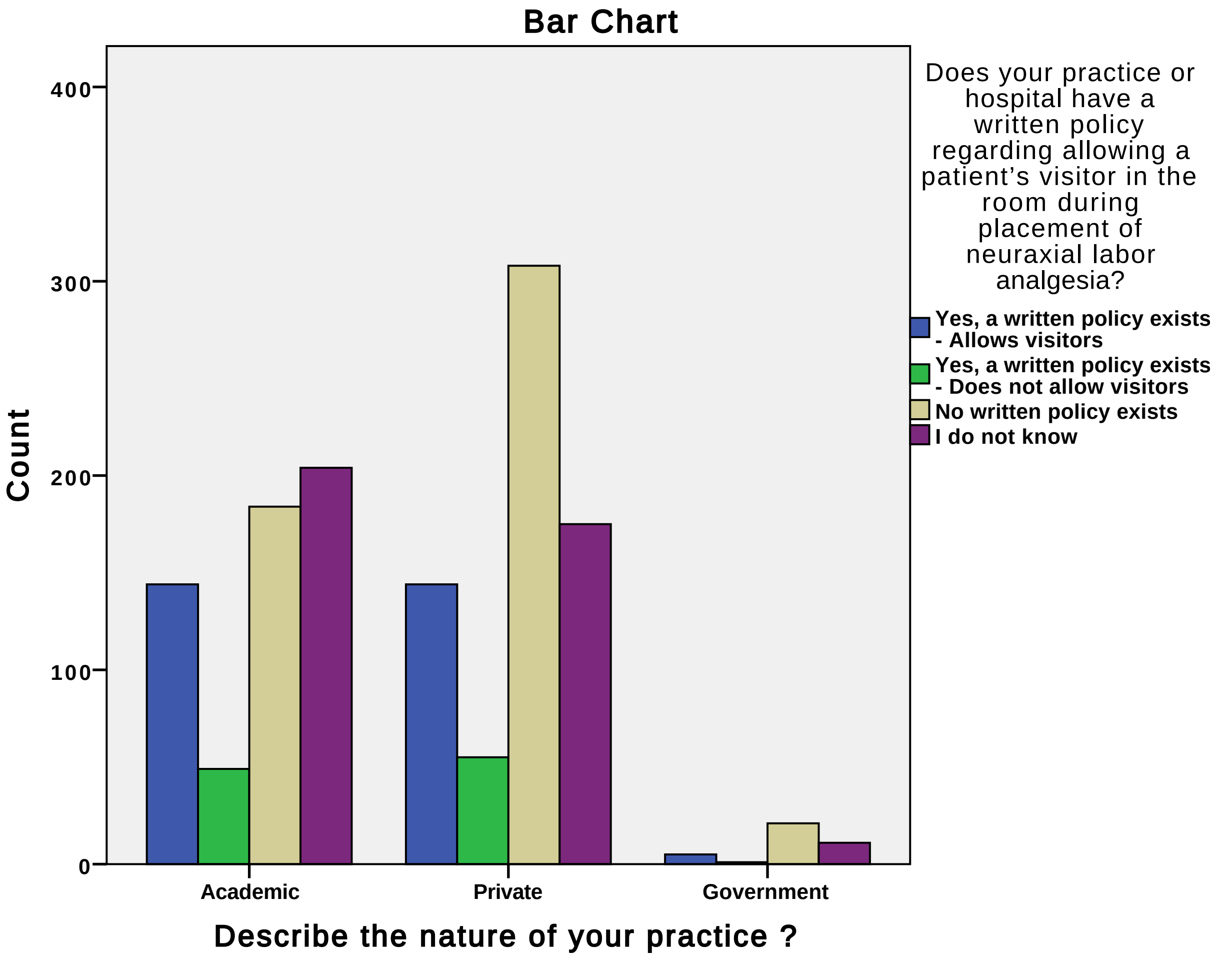

Describe the nature of your practice ? * If no policy existed, would you be open to allowing a patient's visitor in the room during placement of neuraxial labor analgesia?

Crosstab

			If no policy existed, would you be open to allowing a patient's visitor in the room during placement of neuraxial labor analgesia?		Total
			Yes	No	
Describe the nature of your practice ?	Academic	Count	422	159	581
		% within Describe the nature of your practice ?	72.6%	27.4%	100.0%
		Adjusted Residual	-1.8	1.8	
	Private	Count	528	154	682
		% within Describe the nature of your practice ?	77.4%	22.6%	100.0%
		Adjusted Residual	2.0	-2.0	
	Government	Count	27	11	38
		% within Describe the nature of your practice ?	71.1%	28.9%	100.0%
		Adjusted Residual	-.6	.6	
Total		Count	977	324	1301
		% within Describe the nature of your practice ?	75.1%	24.9%	100.0%

Chi-Square Tests

	Value	df	Asymp. Sig. (2-sided)
Pearson Chi-Square	4.185[a]	2	.123
Likelihood Ratio	4.178	2	.124
Linear-by-Linear Association	2.221	1	.136
N of Valid Cases	1301		

a. 0 cells (0.0%) have expected count less than 5. The minimum expected count is 9.46.

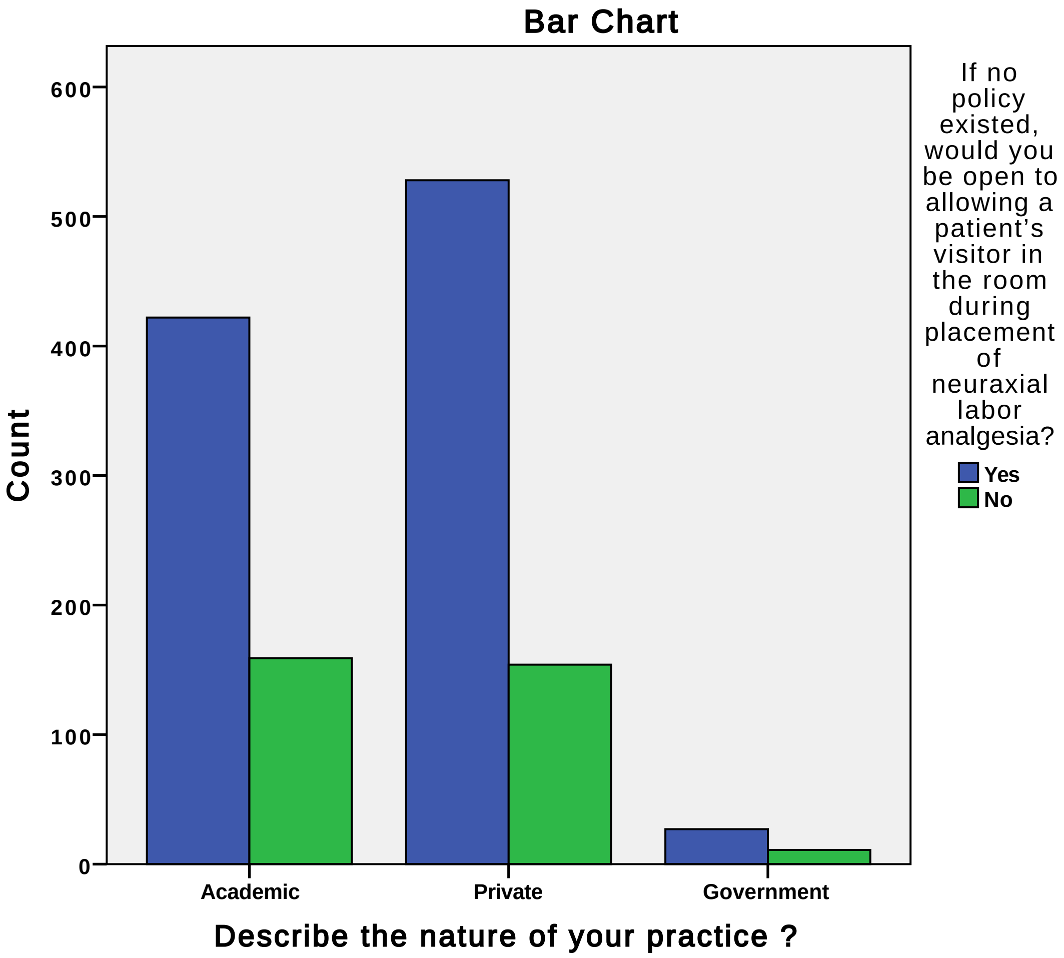

Describe the nature of your practice ? * If you allowed a visitor in the room during placement of neuraxial labor analgesia, you wou ld want the

Crosstab

			If you allowed a visitor in the room during placement of neuraxial labor analgesia, you would want the		
			Visitor always standing	Visitor always sitting	Does not matter
Describe the nature of your practice ?	Academic	Count	5	317	97
		% within Describe the nature of your practice ?	1.2%	75.7%	23.2%
		Adjusted Residual	-.6	2.2	-2.1
	Private	Count	5	371	150
		% within Describe the nature of your practice ?	1.0%	70.5%	28.5%
		Adjusted Residual	-1.4	-1.1	1.5
	Government	Count	4	13	11
		% within Describe the nature of your practice ?	14.3%	46.4%	39.3%
		Adjusted Residual	5.8	-3.1	1.6
Total		Count	14	701	258
		% within Describe the nature of your practice ?	1.4%	72.0%	26.5%

Crosstab

			Total
Describe the nature of your practice ?	Academic	Count	419
		% within Describe the nature of your practice ?	100.0%
		Adjusted Residual	
	Private	Count	526
		% within Describe the nature of your practice ?	100.0%
		Adjusted Residual	
	Government	Count	28
		% within Describe the nature of your practice ?	100.0%
		Adjusted Residual	
Total		Count	973
		% within Describe the nature of your practice ?	100.0%

Chi-Square Tests

	Value	df	Asymp. Sig. (2-sided)
Pearson Chi-Square	40.946[a]	4	.000
Likelihood Ratio	20.326	4	.000
Linear-by-Linear Association	2.709	1	.100
N of Valid Cases	973		

a. 1 cells (11.1%) have expected count less than 5. The minimum expected count is .40.

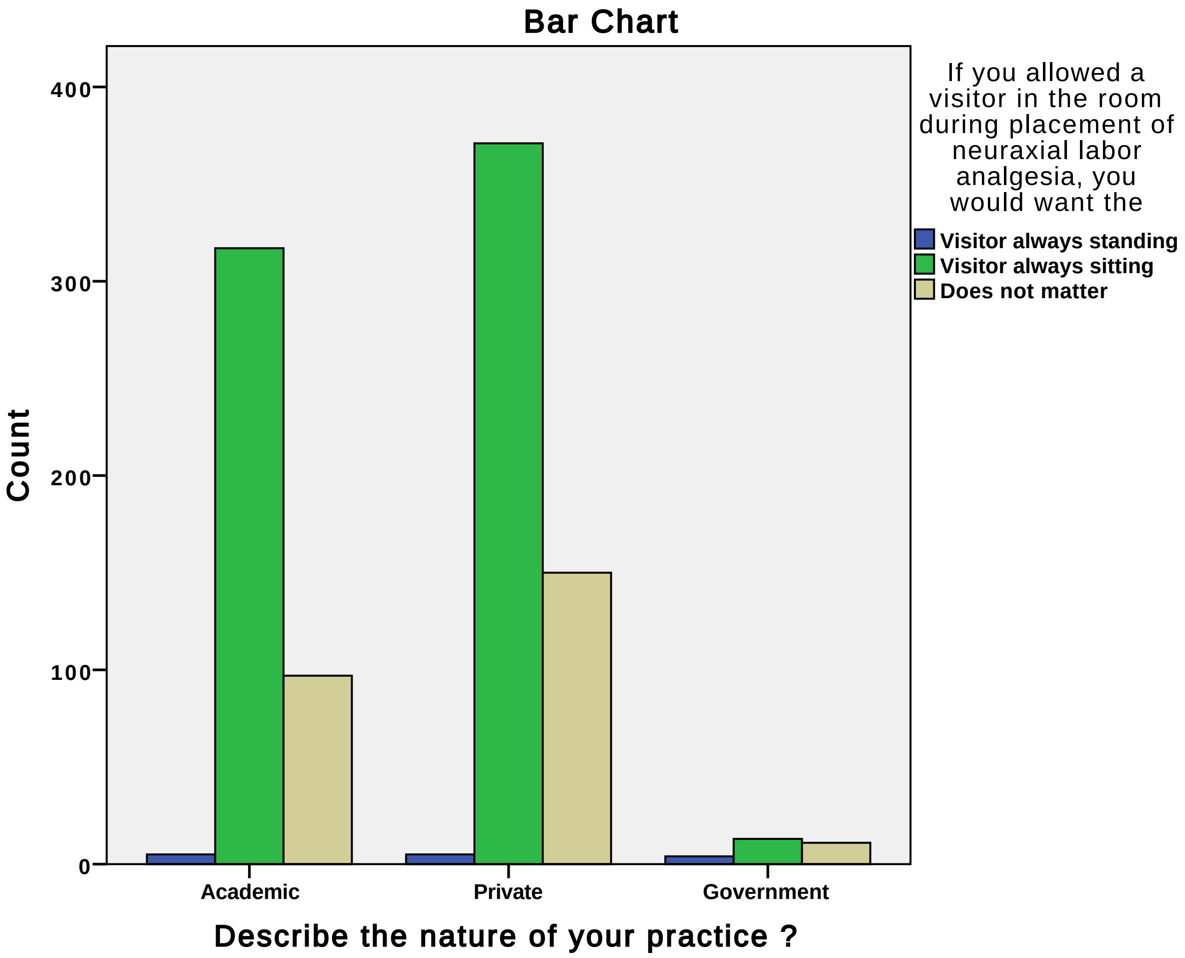

Describe the nature of your practice ? * If you allowed a visitor in the room during placement of neuraxial labor analgesia, you wou ld want the visitor to be positioned such that,

Crosstab

			If you allowed a visitor in the room during placement of neuraxial labor analgesia, you would want the visitor to be positioned such that,		
			Visitor has no view of procedure (patient is in between anes	Visitor has partial view of workspace (but cannot see patien	Does not matter
Describe the nature of your practice ?	Academic	Count	275	79	65
		% within Describe the nature of your practice ?	65.6%	18.9%	15.5%
		Adjusted Residual	2.6	-.1	-3.0
	Private	Count	305	101	120
		% within Describe the nature of your practice ?	58.0%	19.2%	22.8%
		Adjusted Residual	-2.1	.2	2.4
	Government	Count	14	5	9
		% within Describe the nature of your practice ?	50.0%	17.9%	32.1%
		Adjusted Residual	-1.2	-.2	1.6
Total		Count	594	185	194
		% within Describe the nature of your practice ?	61.0%	19.0%	19.9%

Crosstab

			Total
Describe the nature of your practice ?	Academic	Count	419
		% within Describe the nature of your practice ?	100.0%
		Adjusted Residual	
	Private	Count	526
		% within Describe the nature of your practice ?	100.0%
		Adjusted Residual	
	Government	Count	28
		% within Describe the nature of your practice ?	100.0%
		Adjusted Residual	
Total		Count	973
		% within Describe the nature of your practice ?	100.0%

Chi-Square Tests

	Value	df	Asymp. Sig. (2-sided)
Pearson Chi-Square	11.234[a]	4	.024
Likelihood Ratio	11.166	4	.025
Linear-by-Linear Association	10.534	1	.001
N of Valid Cases	973		

a. 0 cells (0.0%) have expected count less than 5. The minimum expected count is 5.32.

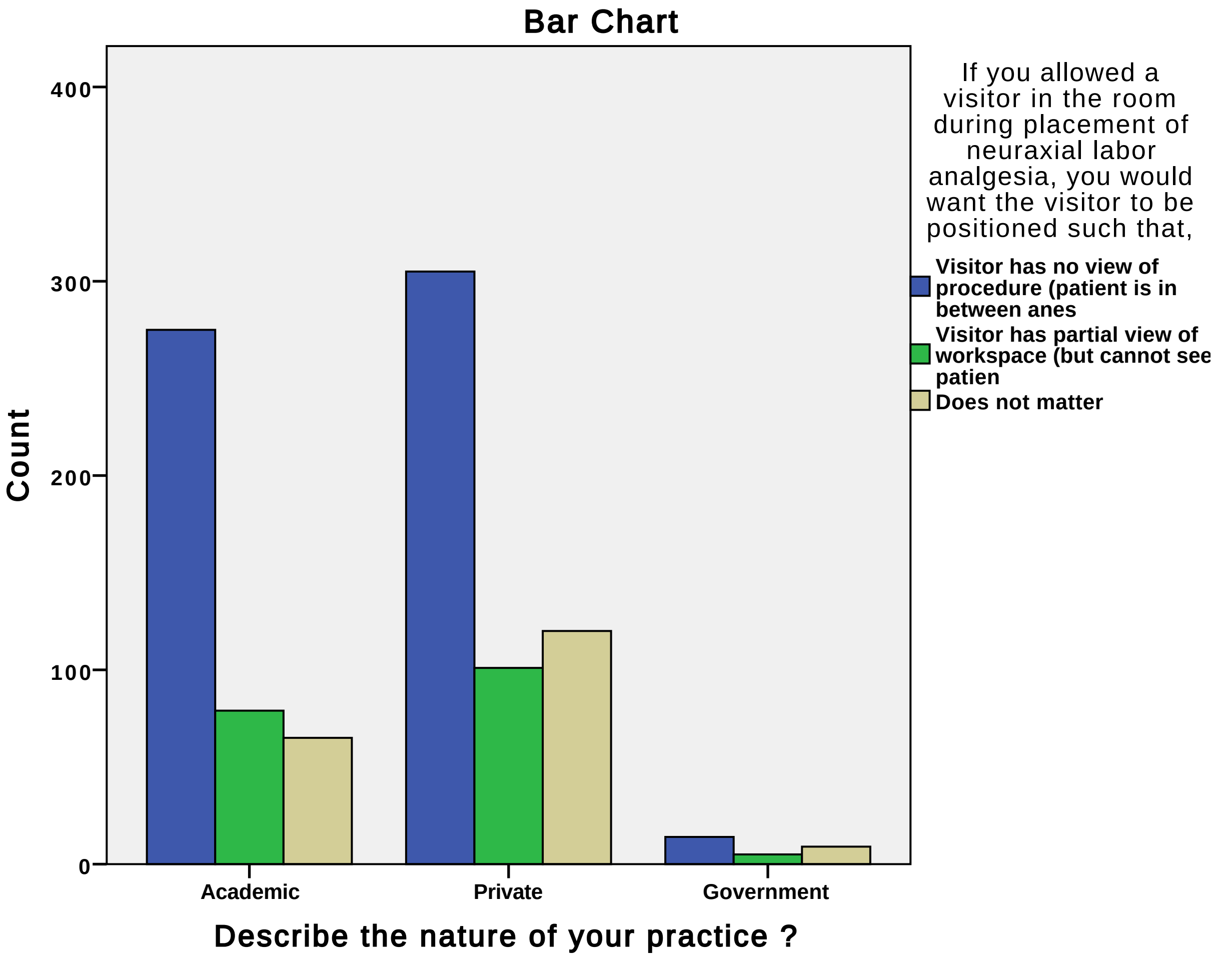

Describe the nature of your practice ? * What would be your singl e most important reason for allowing a patient's visitor in the ro om during placement of neuraxial labor analgesia?

Crosstab

			What would be your single most important reason for allowing a patient's visitor in the room during placement of neuraxial labor ...		
			It would likely reduce patient's anxiety	It would likely reduce visitor's anxiety (for e. g. if visito	Visitor's assistance needed (for e. g. if visitor were a doul
Describe the nature of your practice ?	Academic	Count	263	18	17
		% within Describe the nature of your practice ?	62.8%	4.3%	4.1%
		Adjusted Residual	-1.1	-.9	.4
	Private	Count	343	31	19
		% within Describe the nature of your practice ?	65.2%	5.9%	3.6%
		Adjusted Residual	.4	1.3	-.3
	Government	Count	23	0	1
		% within Describe the nature of your practice ?	82.1%	0.0%	3.6%
		Adjusted Residual	2.0	-1.2	-.1
Total		Count	629	49	37
		% within Describe the nature of your practice ?	64.6%	5.0%	3.8%

Crosstab

			What would be your single most important ...	
			To fulfill patient's request	Total
Describe the nature of your practice ?	Academic	Count	121	419
		% within Describe the nature of your practice ?	28.9%	100.0%
		Adjusted Residual	1.5	
	Private	Count	133	526
		% within Describe the nature of your practice ?	25.3%	100.0%
		Adjusted Residual	-.9	
	Government	Count	4	28
		% within Describe the nature of your practice ?	14.3%	100.0%
		Adjusted Residual	-1.5	
Total		Count	258	973
		% within Describe the nature of your practice ?	26.5%	100.0%

Chi-Square Tests

	Value	df	Asymp. Sig. (2-sided)
Pearson Chi-Square	7.102[a]	6	.312
Likelihood Ratio	8.693	6	.192
Linear-by-Linear Association	3.257	1	.071
N of Valid Cases	973		

a. 2 cells (16.7%) have expected count less than 5. The minimum expected count is 1.06.

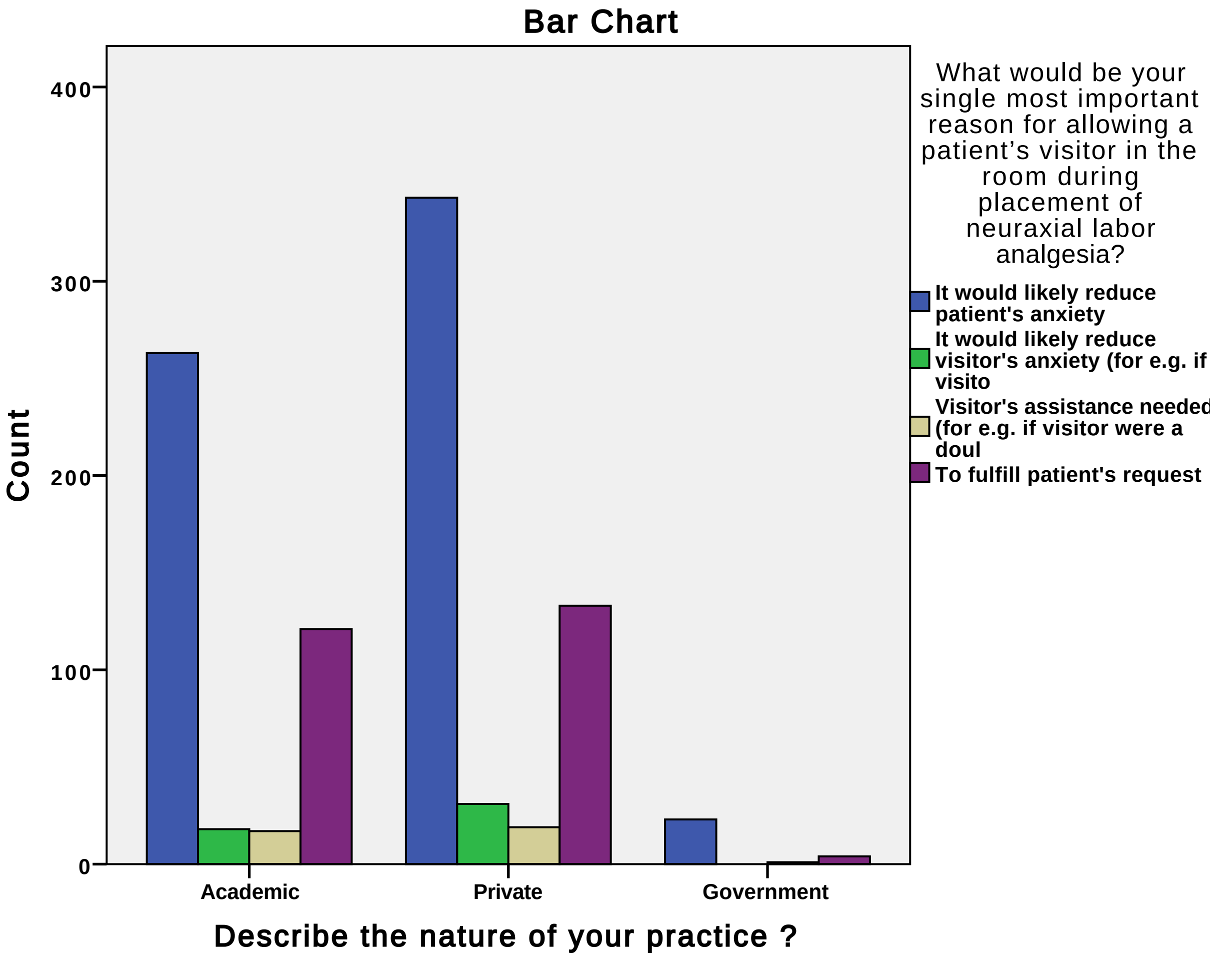

Describe the nature of your practice ? * How often has another non anesthesia member of the labor and delivery team (for example obstetrician or nurse) attempted to influence your decision to have a visitor present during placement of neuraxial labor analgesia?

Crosstab

			How often has another non anesthesia member of the labor and delivery team (for example obstetrician or nurse) attempted to influence your decision to have a visitor present during placement of neuraxial labor analgesia?		
			Rarely (< 5 %)	Occasionally (5 % - 40 %)	Often > 40 %
Describe the nature of your practice ?	Academic	Count	303	77	39
		% within Describe the nature of your practice ?	72.3%	18.4%	9.3%
		Adjusted Residual	-.8	.2	1.1
	Private	Count	393	95	38
		% within Describe the nature of your practice ?	74.7%	18.1%	7.2%
		Adjusted Residual	.8	.0	-1.2
	Government	Count	21	4	3
		% within Describe the nature of your practice ?	75.0%	14.3%	10.7%
		Adjusted Residual	.2	-.5	.5
Total		Count	717	176	80
		% within Describe the nature of your practice ?	73.7%	18.1%	8.2%

Crosstab

			Total
Describe the nature of your practice ?	Academic	Count	419
		% within Describe the nature of your practice ?	100.0%
		Adjusted Residual	
	Private	Count	526
		% within Describe the nature of your practice ?	100.0%
		Adjusted Residual	
	Government	Count	28
		% within Describe the nature of your practice ?	100.0%
		Adjusted Residual	
Total		Count	973
		% within Describe the nature of your practice ?	100.0%

Chi-Square Tests

	Value	df	Asymp. Sig. (2-sided)
Pearson Chi-Square	1.882[a]	4	.758
Likelihood Ratio	1.883	4	.757
Linear-by-Linear Association	.879	1	.349
N of Valid Cases	973		

a. 1 cells (11.1%) have expected count less than 5. The minimum expected count is 2.30.

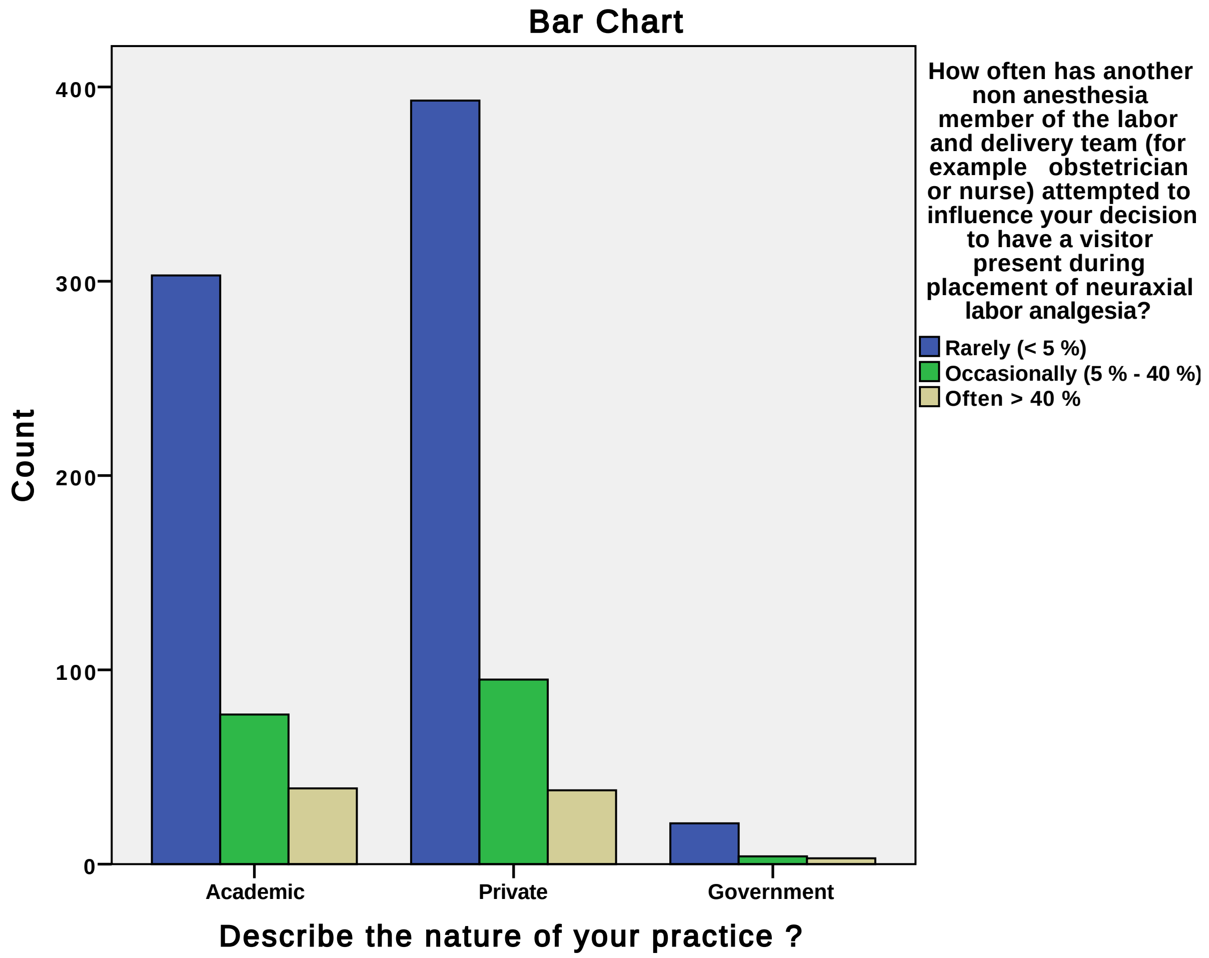

Describe the nature of your practice ? * How often has another non anesthesia member of the labor and delivery team (for example obstetrician or nurse) attempted to influence your decision to NOT have a visitor present during placement of neuraxial labor analgesia?

Crosstab

			How often has another non anesthesia member of the labor and delivery team (for example obstetrician or nurse) attempted to influence your decision to NOT have a visitor present during placement of neuraxial labor analgesia?		
			Rarely < 5 %	Occasionally 5 - 40 %	Often > 40 %
Describe the nature of your practice ?	Academic	Count	128	18	11
		% within Describe the nature of your practice ?	81.5%	11.5%	7.0%
		Adjusted Residual	1.3	-2.1	1.1
	Private	Count	116	32	7
		% within Describe the nature of your practice ?	74.8%	20.6%	4.5%
		Adjusted Residual	-1.6	2.3	-.8
	Government	Count	9	1	0
		% within Describe the nature of your practice ?	90.0%	10.0%	0.0%
		Adjusted Residual	.9	-.5	-.8
Total		Count	253	51	18
		% within Describe the nature of your practice ?	78.6%	15.8%	5.6%

Crosstab

			Total
Describe the nature of your practice ?	Academic	Count	157
		% within Describe the nature of your practice ?	100.0%
		Adjusted Residual	
	Private	Count	155
		% within Describe the nature of your practice ?	100.0%
		Adjusted Residual	
	Government	Count	10
		% within Describe the nature of your practice ?	100.0%
		Adjusted Residual	
Total		Count	322
		% within Describe the nature of your practice ?	100.0%

Chi-Square Tests

	Value	df	Asymp. Sig. (2-sided)
Pearson Chi-Square	6.430[a]	4	.169
Likelihood Ratio	7.001	4	.136
Linear-by-Linear Association	.017	1	.897
N of Valid Cases	322		

a. 2 cells (22.2%) have expected count less than 5. The minimum expected count is .56.

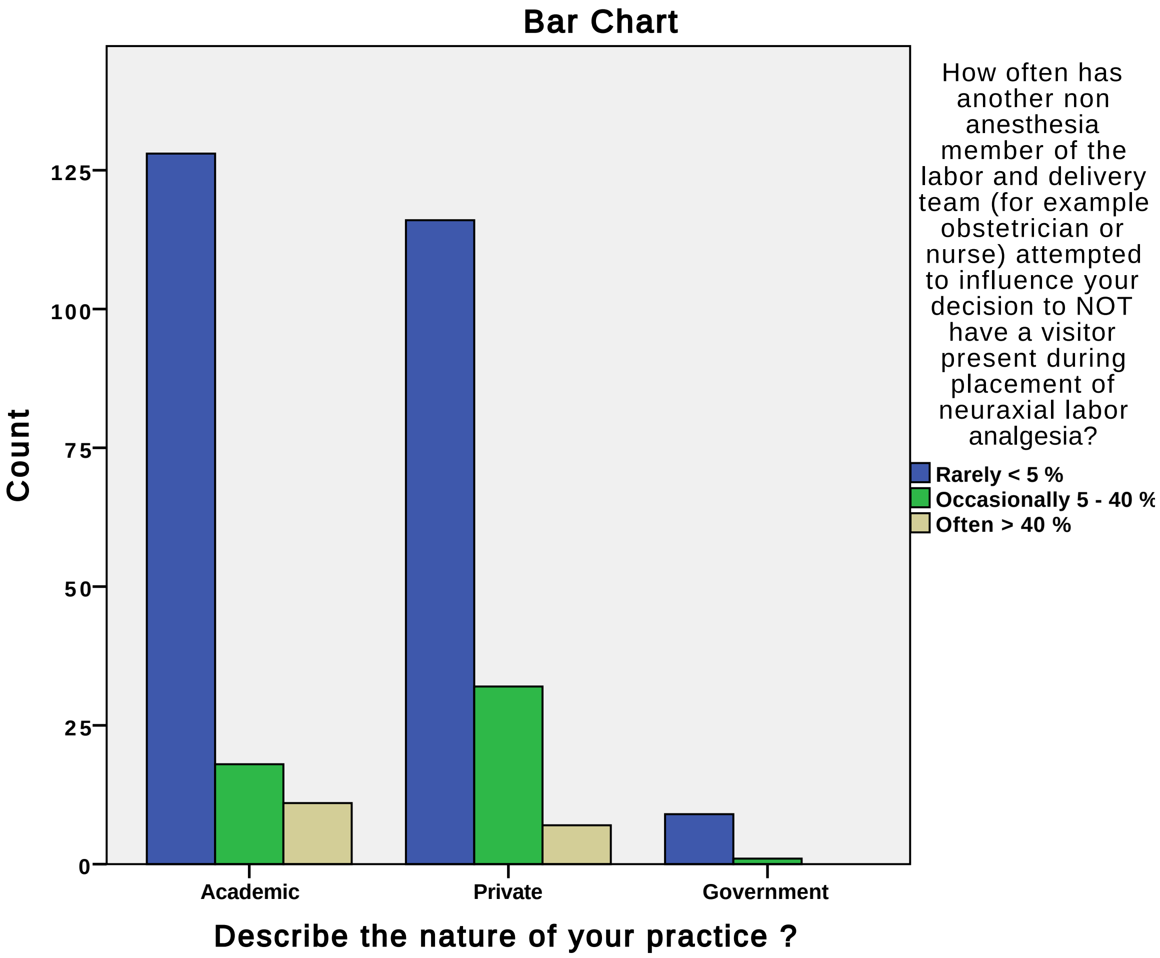

Describe the nature of your practice ? * What would be your single most important reason for NOT allowing a patient's visitor in the room during placement of neuraxial labor analgesia?

Crosstab

			What would be your single most important reason for NOT allowing a patient's visitor in the room during placement of neuraxial labor ..		
			Increase in anesthesiol ogist's stress	Possible interference by visitor(for e.g. comments made if d	Concern about visitor (for e.g. passing out or nc being ab
Describe the nature of your practice ?	Academic	Count	12	73	55
		% within Describe the nature of your practice ?	7.6%	46.5%	35.0%
		Adjusted Residual	.3	-.7	.8
	Private	Count	11	75	51
		% within Describe the nature of your practice ?	7.1%	48.4%	32.9%
		Adjusted Residual	.0	.0	.0
	Government	Count	0	8	0
		% within Describe the nature of your practice ?	0.0%	80.0%	0.0%
		Adjusted Residual	-.9	2.0	-2.3
Total		Count	23	156	106
		% within Describe the nature of your practice ?	7.1%	48.4%	32.9%

Crosstab

			What would be your single most important ...	
			Medico-legal concerns	Total
Describe the nature of your practice ?	Academic	Count	17	157
		% within Describe the nature of your practice ?	10.8%	100.0%
		Adjusted Residual	-.4	
	Private	Count	18	155
		% within Describe the nature of your practice ?	11.6%	100.0%
		Adjusted Residual	.1	
	Government	Count	2	10
		% within Describe the nature of your practice ?	20.0%	100.0%
		Adjusted Residual	.9	
Total		Count	37	322
		% within Describe the nature of your practice ?	11.5%	100.0%

Chi-Square Tests

	Value	df	Asymp. Sig. (2-sided)
Pearson Chi-Square	7.145[a]	6	.308
Likelihood Ratio	10.693	6	.098
Linear-by-Linear Association	.028	1	.867
N of Valid Cases	322		

a. 4 cells (33.3%) have expected count less than 5. The minimum expected count is .71.

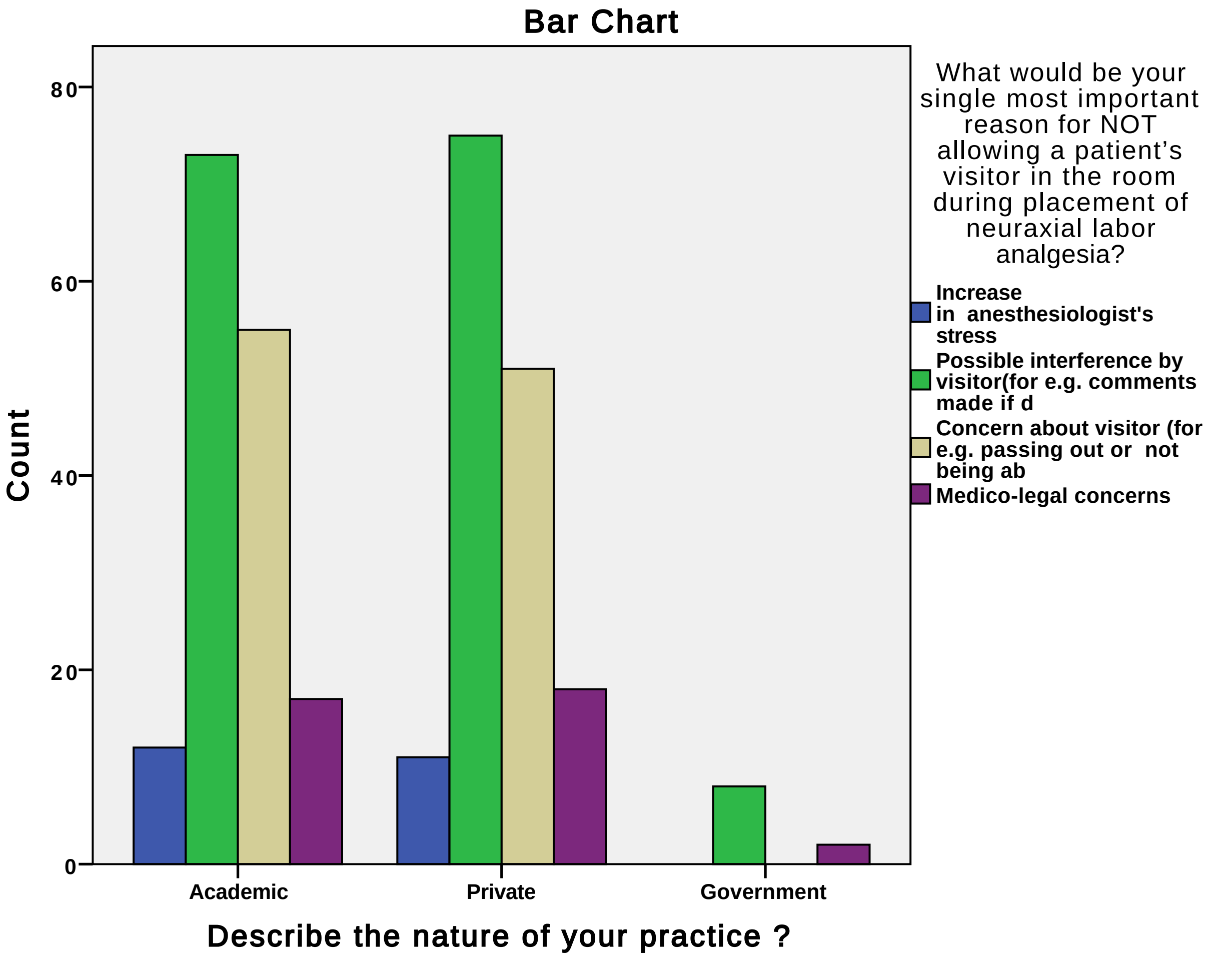
Bar Chart
Count
80
60
40
20
0
Academic
Private
Government
Describe the nature of your practice ?
What would be your single most important reason for NOT allowing a patient's visitor in the room during placement of neuraxial labor analgesia?
Increase in anesthesiologist's stress
Possible interference by visitor(for e.g. comments made if d
Concern about visitor (for e.g. passing out or not being ab
Medico-legal concerns
